# Genome-wide analysis of 102,084 migraine cases identifies 123 risk loci and subtype-specific risk alleles

**DOI:** 10.1038/s41588-021-00990-0

**Published:** 2022-02-03

**Authors:** Heidi Hautakangas, Bendik S. Winsvold, Sanni E. Ruotsalainen, Gyda Bjornsdottir, Aster V. E. Harder, Lisette J. A. Kogelman, Laurent F. Thomas, Raymond Noordam, Christian Benner, Padhraig Gormley, Ville Artto, Karina Banasik, Anna Bjornsdottir, Dorret I. Boomsma, Ben M. Brumpton, Kristoffer Sølvsten Burgdorf, Julie E. Buring, Mona Ameri Chalmer, Irene de Boer, Martin Dichgans, Christian Erikstrup, Markus Färkkilä, Maiken Elvestad Garbrielsen, Mohsen Ghanbari, Knut Hagen, Paavo Häppölä, Jouke-Jan Hottenga, Maria G. Hrafnsdottir, Kristian Hveem, Marianne Bakke Johnsen, Mika Kähönen, Espen S. Kristoffersen, Tobias Kurth, Terho Lehtimäki, Lannie Lighart, Sigurdur H. Magnusson, Rainer Malik, Ole Birger Pedersen, Nadine Pelzer, Brenda W. J. H. Penninx, Caroline Ran, Paul M. Ridker, Frits R. Rosendaal, Gudrun R. Sigurdardottir, Anne Heidi Skogholt, Olafur A. Sveinsson, Thorgeir E. Thorgeirsson, Henrik Ullum, Lisanne S. Vijfhuizen, Elisabeth Widén, Ko Willems van Dijk, International Headache Genetics Consortium, Irene de Boer, Arn M. J. M. van den Maagdenberg, HUNT All-in Headache, Danish Blood Donor Study Genomic Cohort, Arpo Aromaa, Andrea Carmine Belin, Tobias Freilinger, M. Arfan Ikram, Marjo-Riitta Järvelin, Olli T. Raitakari, Gisela M. Terwindt, Mikko Kallela, Maija Wessman, Jes Olesen, Daniel I. Chasman, Dale R. Nyholt, Hreinn Stefánsson, Kari Stefansson, Arn M. J. M. van den Maagdenberg, Thomas Folkmann Hansen, Samuli Ripatti, John-Anker Zwart, Aarno Palotie, Matti Pirinen

**Affiliations:** 1 Institute for Molecular Medicine Finland (FIMM), Helsinki Institute of Life Science (HiLIFE), University of Helsinki, Helsinki, Finland; 2 Department of Research, Innovation and Education, Division of Clinical Neuroscience, Oslo University Hospital, Oslo, Norway; 3 K. G. Jebsen Center for Genetic Epidemiology, Department of Public Health and Nursing, Faculty of Medicine and Health Sciences, Norwegian University of Science and Technology, Trondheim, Norway; 4 Department of Neurology, Oslo University Hospital, Oslo, Norway; 5 deCODE genetics/Amgen Inc., Reykjavik, Iceland; 6 Department of Neurology, Leiden University Medical Center, Leiden, the Netherlands; 7 Department of Human Genetics, Leiden University Medical Center, Leiden, the Netherlands; 8 Danish Headache Center, Department of Neurology, Copenhagen University Hospital, Copenhagen, Denmark; 9 Department of Clinical and Molecular Medicine, Norwegian University of Science and Technology, Trondheim, Norway; 10 BioCore - Bioinformatics Core Facility, Norwegian University of Science and Technology, Trondheim, Norway; 11 Clinic of Laboratory Medicine, St. Olavs Hospital, Trondheim University Hospital, Trondheim, Norway; 12 Department of Internal Medicine, Section of Gerontology and Geriatrics, Leiden University Medical Center, Leiden, the Netherlands; 13 GlaxoSmithKline, Cambridge, MA USA; 14 Department of Neurology, Helsinki University Central Hospital, Helsinki, Finland; 15 Novo Nordic Foundation Center for Protein Research, Copenhagen University, Copenhagen, Denmark; 16 Neurology Private Practice, Laeknasetrid, Reykjavik, Iceland; 17 Netherlands Twin Register, Department of Biological Psychology, Vrije Universiteit, Amsterdam, the Netherlands; 18 Department of Clinical Immunology, Copenhagen University Hospital, Rigshospitalet Copenhagen, Denmark; 19 Division of Preventive Medicine, Brigham and Women’s Hospital, Boston, MA USA; 20 Harvard Medical School, Boston, MA USA; 21 Institute for Stroke and Dementia Research, University Hospital, LMU Munich, Munich, Germany; 22 Munich Cluster for Systems Neurology (Synergy), Munich, Germany; 23 Department of Clinical Immunology, Aarhus University Hospital, Aarhus, Denmark; 24 Department of Epidemiology, Erasmus University Medical Center, Rotterdam, the Netherlands; 25 Department of Neuromedicine and Movement Science, Faculty of Medicine and Health Sciences, Norwegian University of Science and Technology (NTNU), Trondheim, Norway; 26 Clinical Research Unit Central Norway, St. Olavs University Hospital, Trondheim, Norway; 27 Landspitali University Hospital, Reykjavik, Iceland; 28 HUNT Research Center, Department of Public Health and Nursing, Faculty of Medicine and Health Sciences, Norwegian University of Science and Technology, Trondheim, Norway; 29 Institute of Clinical Medicine, Faculty of Medicine, University of Oslo, Oslo, Norway; 30 Research and Communication Unit for Musculoskeletal Health (FORMI), Department of Research, Innovation and Education, Division of Clinical Neuroscience, Oslo University Hospital, Oslo, Norway; 31 Department of Clinical Physiology, Tampere University Hospital, and Finnish Cardiovascular Research Center - Tampere, Faculty of Medicine and Health Technology, Tampere University, Tampere, Finland; 32 Department of General Practice, Institute of Health and Society, University of Oslo, Oslo, Norway; 33 Department of Neurology, Akershus University Hospital, Lørenskog, Norway; 34 Institute of Public Health, Charité – Universitätsmedizin Berlin, Berlin, Germany; 35 Department of Clinical Chemistry, Fimlab Laboratories, and Finnish Cardiovascular Research Center - Tampere, Faculty of Medicine and Health Technology, Tampere University, Tampere, Finland; 36 Department of Clinical Immunology, Zealand University Hospital, Køge, Denmark; 37 Department of Psychiatry, Amsterdam UMC, Vrije Universiteit, Amsterdam Public Health Research Institute, Amsterdam, the Netherlands; 38 GGZ inGeest Specialized Mental Health Care, Amsterdam, the Netherlands; 39 Department of Neuroscience, Karolinska Institutet, Stockholm, Sweden; 40 Department of Clinical Epidemiology, Leiden University Medical Center, Leiden, the Netherlands; 41 Department of Internal Medicine, Division of Endocrinology, Leiden University Medical Center, Leiden, the Netherlands; 42 National Public Health Institute (Finnish Institute for Health and Welfare - THL), Helsinki, Finland; 43 Klinikum Passau, Department of Neurology, Passau, Germany; 44 Centre of Neurology, Hertie Institute for Clinical Brain Research, Tuebingen, Germany; 45 Department of Epidemiology and Biostatistics, MRC-PHE Centre for Environment and Health, School of Public Health, Imperial College London, London, UK; 46 Center for Life Course Health Research, Faculty of Medicine, University of Oulu, Oulu, Finland; 47 Unit of Primary Health Care, Oulu University Hospital, Oulu, Finland; 48 Department of Life Sciences, College of Health and Life Sciences, Brunel University London, London, UK; 49 Centre for Population Health Research, University of Turku and Turku University Hospital, Turku, Finland; 50 Research Centre of Applied and Preventive Cardiovascular Medicine, University of Turku, Turku, Finland; 51 Department of Clinical Physiology and Nuclear Medicine, Turku University Hospital, Turku, Finland; 52 Folkhälsan Research Center, Helsinki, Finland; 53 School of Biomedical Sciences and Centre for Genomics and Personalised Health, Faculty of Health, Queensland University of Technology, Brisbane, QLD Australia; 54 Broad Institute of MIT and Harvard, Cambridge, MA USA; 55 Department of Public Health, University of Helsinki, Helsinki, Finland; 56 Analytic and Translational Genetics Unit, Department of Medicine, Department of Neurology and Department of Psychiatry, Massachusetts General Hospital, Boston, MA USA; 57 The Stanley Center for Psychiatric Research and Program in Medical and Population Genetics, The Broad Institute of MIT and Harvard, Cambridge, MA USA; 58 Department of Mathematics and Statistics, University of Helsinki, Helsinki, Finland

**Keywords:** Migraine, Genome-wide association studies

## Abstract

Migraine affects over a billion individuals worldwide but its genetic underpinning remains largely unknown. Here, we performed a genome-wide association study of 102,084 migraine cases and 771,257 controls and identified 123 loci, of which 86 are previously unknown. These loci provide an opportunity to evaluate shared and distinct genetic components in the two main migraine subtypes: migraine with aura and migraine without aura. Stratification of the risk loci using 29,679 cases with subtype information indicated three risk variants that seem specific for migraine with aura (in *HMOX2*, *CACNA1A* and *MPPED2*), two that seem specific for migraine without aura (near *SPINK2* and near *FECH*) and nine that increase susceptibility for migraine regardless of subtype. The new risk loci include genes encoding recent migraine-specific drug targets, namely calcitonin gene-related peptide (*CALCA/CALCB*) and serotonin 1F receptor (*HTR1F*). Overall, genomic annotations among migraine-associated variants were enriched in both vascular and central nervous system tissue/cell types, supporting unequivocally that neurovascular mechanisms underlie migraine pathophysiology.

## Abstract (web summary)

Genome-wide association analyses identify 123 susceptibility loci for migraine and implicate neurovascular mechanisms in its pathophysiology. Subtype analyses highlight risk loci specific for migraine with or without aura in addition to shared risk variants.

## Main

Migraine is a highly prevalent brain disorder characterized by disabling attacks of moderate-to-severe pulsating and usually one-sided headache that may be aggravated by physical activity, and can be associated with symptoms such as a hypersensitivity to light and sound, nausea and vomiting^1^. Migraine has a lifetime prevalence of 15–20% and is ranked as the second most disabling condition in terms of years lived with disability^2,3^. Migraine is three times more prevalent in females than in males. For about one-third of patients, migraine attacks often include an aura phase^4^ characterized by transient neurological symptoms such as scintillations. Hence, the two main migraine subtypes are defined as migraine with aura (MA) and migraine without aura (MO).

It has been debated for decades whether or not the migraine subtypes are in fact two separate disorders^5–7^, and, if so, what the underlying causes are. Prevailing theories about migraine pathophysiology emphasize neuronal and/or vascular dysfunction^8,9^. Current knowledge of disease mechanisms comes largely from studies of a rare monogenic subform of MA—familial hemiplegic migraine—for which three ion transporter genes (*CACNA1A*, *ATP1A2* and *SCN1A*) have been identified^10^. The common forms of migraine (MA and MO) instead have a complex polygenic architecture with an increased familial relative risk^5^, increased concordance in monozygotic twins^11^ and a heritability of 40–60%^12^. The largest genome-wide association study (GWAS) thus far, with 59,674 cases and 316,078 controls, reported 38 genomic loci that confer migraine risk^13^. Subsequent analyses of these GWAS data showed enrichment of migraine signals near activating histone marks specific to cardiovascular and central nervous system (CNS) tissues^14^, as well as for genes expressed in vascular and smooth muscle tissues^13^. Other smaller GWAS^15–21^ have suggested ten additional loci. Of note, the previous datasets were too small to perform a meaningful comparison of the genetic background between migraine subtypes.

As migraine is globally the second largest contributor to years lived with disability^2,3^, there is clearly a large need for new treatments. Triptans, that is, serotonin 5-HT_1B/1D_ receptor agonists, are migraine-specific acute treatments for the headache phase but are not effective in every patient, whereas preventive medication is far from satisfactory^22^. Recent promising alternatives for acute treatment are serotonin 5-HT_1F_ receptor agonists (‘ditans’)^23^ and small-molecule calcitonin gene-related peptide (CGRP) receptor antagonists (‘gepants’)^24,25^. For preventive treatment, monoclonal antibodies (mAbs) targeting CGRP or its receptor have recently proven effective^26^, and new gepants for migraine prevention are under development^27^. Still, there remains an urgent need for treatment options for patients who do not respond to existing treatments. Genetics has proven promising in developing new therapeutic hypotheses in other prevalent complex diseases, such as cardiovascular disease^28^ and type 2 diabetes^29^, and we anticipate that large genetic studies of migraine could also yield similar insights.

We conducted a GWAS meta-analysis of migraine by adding to the previous meta-analysis^13^ 42,410 new migraine cases from four study collections (Table 1). This increased the number of migraine cases by 71% for a total sample of 102,084 cases and 771,257 controls. Furthermore, we assessed the subtype specificity of the risk loci in 8,292 new MA and 6,707 new MO cases in addition to the 6,332 MA and 8,348 MO cases used previously^13^ (Table 2). Here, we report 123 genomic loci, of which 86 are previously unknown, and include the first four loci that reach genome-wide significance (*P* < 5 × 10^−8^) in MA. Our subtype data show compellingly that migraine risk is conferred both by risk loci that seem specific for only one subtype as well as by loci that are shared by both subtypes. Our findings also include new risk loci containing target genes of recent migraine drugs acting on the CGRP pathway and the serotonin 5-HT_1F_ receptor. Finally, our data support the concept that migraine is brought about by both neuronal and vascular genetic factors, strengthening the view that migraine is truly a neurovascular disorder.

**Table 1:** Five migraine study collections included in the meta-analysis

Abbreviation	Full name	Ancestry	Cases	Controls	Case %	Migraine definition
IHGC2016^a^	Gormley et al. 2016 (ref. ^13^) (no 23andMe)	European descent	29,209	172,931	14.4	Self-reported and ICHD-II
23andMe^b^	23andMe, Inc. (23andMe.com)	European descent	53,109	230,876	18.7	Self-reported
UKBB	UK Biobank (ukbiobank.ac.uk)	European, British	10,881	330,170	3.2	Self-reported
GeneRISK	GeneRISK (generisk.fi)	European, Finnish	1,084	4,857	18.2	Self-reported
HUNT	Nord-Trøndelag Health Study (ntnu.edu/hunt)	European, Norwegian	7,801	32,423	19.4	Self-reported migraine or fulfilling modified ICHD-II criteria

^a^IHGC2016 is a meta-analysis of 21 studies listed in Supplementary Table 1 and does not include data from 23andMe. Some studies of IHGC2016 determined migraine status through clinical phenotyping, while migraine status in other studies is based on self-reported information.

^b^23andMe includes 30,465 cases from the meta-analysis of Gormley et al.^13^ and 22,644 new cases. ICHD-II, International Classification of Headache Disorders second edition.

**Table 2:** Study collections included in MO and MA subtype analyses

Abbreviation	Full name	Ancestry	Subtype	Cases	Controls
IHGC2016^a^	Gormley et al. 2016 (ref. ^13^)	European descent	MO	8,348	139,622
			MA	6,332	144,883
UKBB	UK Biobank (ukbiobank.ac.uk)	European, British	MO	187	320,139
			MA	1,333	320,139
deCODE	deCODE Genetics Inc.	European, Icelandic	MO	1,648	193,050
			MA	2,297	209,338
DBDS	Danish Blood Donor Study	European, Danish	MO	3,756	28,045
			MA	3,938	28,045
LUMINA	LUMINA migraine without aura or with aura	European, Dutch	MO	1,116	1,445
			MA	724	1,447

^a^IHGC2016 MO is a meta-analysis of 11 studies and IHGC2016 MA is a meta-analysis of 12 studies listed in Gormley et al.^13^.

## Results

### Genome-wide meta-analysis

We combined data on 873,341 individuals of European ancestry (102,084 cases and 771,257 controls) from five study collections (Table 1 and Supplementary Table 1) and analyzed 10,843,197 common variants (Methods). Despite different approaches to the ascertainment of migraine cases across studies, pairwise genetic correlations were all near 1 (Supplementary Table 2), as determined by linkage disequilibrium (LD) score (LDSC) regression^30^, showing high genetic and phenotypic similarity across the studies, justifying their meta-analysis. Pairwise LDSC intercepts were all near 0, indicating little or no sample overlap (Supplementary Table 2).

The genomic inflation factor (λ_GC_) of the fixed-effect meta-analysis results was 1.33 (Supplementary Fig. 1), which is in line with other large meta-analyses^31–33^ and is as expected for a polygenic trait^34^. The univariate LDSC^35^ intercept was 1.05 (s.e. 0.01), which, being close to 1.0, suggests that most of the genome-wide elevation of the association statistics comes from true additive polygenic effects rather than from a confounding bias such as population stratification. The LDSC analysis showed a linear trend between the variant’s LD score and its association with migraine, as expected from a highly polygenic phenotype such as migraine (Supplementary Fig. 2). The SNP heritability estimate from LDSC was 11.2% (95% confidence interval (CI) 10.8–11.6%) on a liability scale when assuming a population prevalence of 16%.

**Fig. 1:**
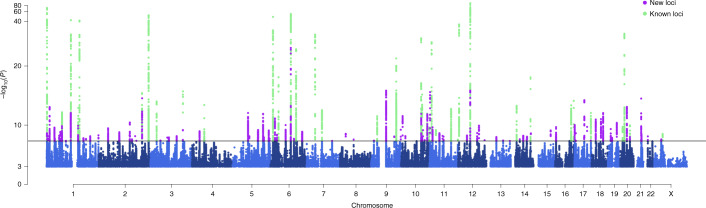
Manhattan plot of migraine GWAS meta-analysis (*n* = 873,341; 102,084 cases and 771,257 controls). On the *x* axis, variants are plotted along the 22 autosomes and the X chromosome. The *y* axis shows the statistical strength of the association from the inverse–variance weighted fixed-effect meta-analysis as the negative log_10_ of the uncorrected two-sided *P* value (*P*). The horizontal line is the genome-wide significance threshold (*P* = 5 × 10^−8^). The 123 risk loci passing the threshold are divided into 86 new loci (purple) and 37 previously known loci (green). Adjacent chromosomes are colored in different shades of blue. Plotted are variants with *P* < 0.001.

We identified 8,117 genome-wide significant (GWS; *P* < 5 × 10^−8^) variants represented by 170 LD-independent index variants (*r*^2^ < 0.1). We defined the risk loci by including all variants in high LD (*r*^2^ > 0.6) with the index variants and merged loci that were closer than 250 kb (Methods). This resulted in 123 independent risk loci (Fig. 1, Supplementary Table 3a and Supplementary Data 1 and 2). Of the 123 loci, 86 are previously unknown, whereas 36 overlap with the previously reported 47 autosomal risk loci (Supplementary Table 4) and one with the previously reported X chromosome risk locus. Of the 11 previously reported migraine risk loci that were not GWS in our study, 6 were GWS in Gormley et al.^13^ and had *P* < 3.50 × 10^−5^ in our data, 1 had *P* = 2.37 × 10^−3^, 3 had *P* > 0.14 and 1 was not available in our data (Supplementary Data 3). When we represented each risk locus by its lead variant, that is, the variant with the smallest *P* value, 47 GWS variants were LD-independent (*r*^2^ < 0.1) of the 123 lead variants, and, with a more stringent threshold (*r*^2^ < 0.01), 15 GWS variants remained LD independent of the 123 lead variants (Supplementary Table 5).

In addition, we conducted an approximate stepwise conditional analysis for the 123 risk loci (Methods). Since sample sizes per variant varied considerably, we restricted the conditional analysis to variants with similar effective sample sizes to the lead variant. The conditional analysis returned six single nucleotide polymorphisms (SNPs) within the 123 risk loci that remained GWS after conditioning on the lead variants (Supplementary Table 6a,b).

### Characterization of migraine risk loci

We mapped the 123 risk loci to genes by their physical location using the Ensembl Variant Effect Predictor (VEP)^36^. Of the lead variants, 59% (72/123) were within a transcript of a protein-coding gene, and 80% (99/123) of the loci contained at least one protein-coding gene within 20 kb, and 93% (114/123) within 250 kb (Supplementary Table 3). Of the 123 lead variants, 5 were missense variants (in genes *PLCE1*, *MRGPRE*, *SERPINA1*, *ZBTB4* and *ZNF462*), and 40 more missense variants were in high LD (*r*^2^ > 0.6) with the lead variants (Supplementary Table 7a). Of note, three variants with a predicted high impact consequence on protein function were in high LD with the lead variants: (1) a stop gained variant (rs34358) with lead variant rs42854 (*r*^2^ = 0.85) in gene *ANKDD1B*, (2) a splice donor variant (rs66880209) with lead variant rs1472662 (*r*^2^ = 0.71) in *RP11-420K8.1* and (3) a splice acceptor variant (rs11042902) with lead variant rs4910165 (*r*^2^ = 0.69) in *MRVI1* (Supplementary Table 7b).

We used stratified LDSC (S-LDSC) to partition migraine heritability by 24 functional genomic annotations^37,38^. We observed enrichment for ten categories (Supplementary Fig. 3 and Supplementary Table 8), with conserved regions showing the highest enrichment (11.2-fold; *P* = 1.95 × 10^−10^), followed by coding regions (8.1-fold; *P* = 1.36 × 10^−3^) and enhancers (4.2-fold; *P* = 3.64 × 10^−4^).

### Prioritization of candidate genes

We mapped the 123 lead variants to genes via expression quantitative trait locus (eQTL) association using GTEx v.8 (ref. ^39^) and data repositories included in FUMA^40^ at a false discovery rate (FDR) of 5% (Methods). The lead variants were cis-eQTLs for 589 genes (Supplementary Table 9), and variants in high LD with the lead variants were cis-eQTLs for an additional 624 genes (Supplementary Table 10). In total, 84% (103/123) of lead variants were cis-eQTLs for at least one gene. Tibial artery had the highest number (47/123) of lead variants as cis-eQTLs in GTEx v.8, and it was the only tissue type where the enrichment was statistically higher (*P* = 6.37 × 10^−6^) than expected based on the overall number of cis-eQTLs per tissue reported by GTEx (Supplementary Fig. 4 and Supplementary Note).

To prioritize candidate genes for the risk loci, we applied two approaches based on GTEx v.8 expression data: fine-mapping of causal gene-sets by FOCUS^41^ (Supplementary Table 11a) and a transcriptome-wide association study (TWAS) by S-PrediXcan^42^ combined with colocalization analysis using COLOC^43^ (Supplementary Table 11b).

With posterior probability (PP) > 0.5, FOCUS found candidate genes for 82 loci and S-PrediXcan + COLOC supported colocalization for 52 loci (Supplementary Table 11c). In total, 73 genes in 46 loci were prioritized by both methods. *MRC2* and *PHACTR1* were the only genes that both methods prioritized with strong evidence (PP > 0.99 for same tissue) and without any other gene prioritized within their loci.

**Fig. 2:**
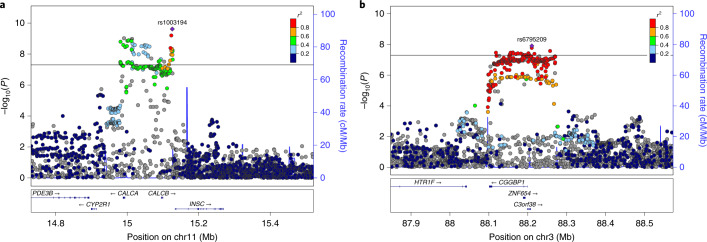
LocusZoom plots of two previously unknown migraine loci with genes that are targets of recent migraine-specific drugs. **a**, Locus containing *CALCA* and *CALCB* genes, encoding CGRP, which is the target of preventive and acute therapies via monoclonal antibodies and gepants. **b**, Locus containing the *HTR1F* gene, which encodes a serotonin 5-HT_1F_ receptor that is the target of acute therapies via ditans. The *x* axis shows the chromosomal location, and the *y* axis shows the uncorrected two-sided negative log_10_ (*P*) from the inverse–variance weighted fixed-effects meta-analysis with 102,084 cases and 771,257 controls. The squared correlation to the lead variant is shown by colors based on the UK Biobank data for variants that have an effective sample size ±20% of the lead variant’s effective sample size. Horizontal line corresponds to *P* = 5 × 10^−8^. Blue graph shows the recombination rate.

Two of the new risk loci contain genes (*CALCA*/*CALCB* and *HTR1F*) whose protein products are closely related to targets of two migraine-specific drug therapies^44^. We observe a convincing association at the chromosome 11 locus that contains the *CALCA* and *CALCB* genes encoding CGRP itself (lead SNP rs1003194, *P* = 2.43 × 10^−10^; Fig. 2a), while none of the genes encoding CGRP receptor proteins (*CALCRL*, *RAMP1* or *RCP*) show a statistically comparable association (all *P* > 10^−4^; Supplementary Fig. 5). Variant rs1003194 is a cis-eQTL for *CALCB*, but also for *COPB1*, *PDE3B* and *INSC* (Supplementary Table 9) and FOCUS prioritizes *CALCA*, *CALCB* and *INSC* (Supplementary Table 11c). In addition, a new locus on chromosome 3 contains *HTR1F* (lead SNP rs6795209, *P* = 1.23 × 10^−8^; Fig. 2b), which encodes the serotonin 5-HT_1F_ receptor. Variant rs6795209 is a significant cis-eQTL for *HTR1F*, as well as for three other genes (*CGGBP1*, *ZNF654*, *C3orf38*) in the same locus (Supplementary Table 9). FOCUS or S-PrediXcan + COLOC did not prioritize *HTR1F* based on gene expression data (Supplementary Table 11c).

### Migraine subtypes with aura and without aura

**Fig. 3:**
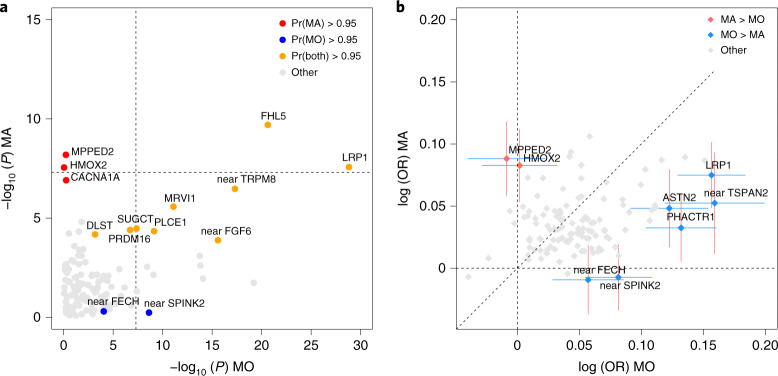
Lead variants stratified by migraine subtype for risk loci with MAF >5%. **a**, Axes show the negative log_10_ (*P*) of MO (*x* axis; *n* = 697,356, 15,055 MO cases and 682,301 controls) and MA (*y* axis; *n* = 718,476, 14,624 MA cases and 703,852 controls) analyses. Two-sided *P* values are derived from inverse–variance weighted fixed-effect meta-analyses and are uncorrected. Symbols that are colored and annotated indicate >95% posterior probability that a nonzero effect is present in both MO and MA (model BOTH), or that the effect is present only in MO or only in MA but not both (models MO and MA, respectively). Variants with a probability <95% for each of the three models are shown as gray. Dashed lines show the genome-wide significance threshold (*P* = 5 × 10^−8^). **b**, Axes show logarithm of odds ratios (OR) for MO (*x* axis; *n* = 697,356, 15,055 MO cases and 682,301 controls) and MA (*y* axis; *n* = 718,476, 14,624 MA cases and 703,852 controls) calculated for the migraine risk allele. The effects at variants that have been colored and annotated differ between the subtypes at significance level of 0.0004 = 0.05/123. The 95% CIs for the logarithm of odds ratios are shown for the annotated variants. Dashed lines show the coordinate axes and the diagonal.

Previously, Gormley et al.^13^ conducted subtype-specific GWAS with 6,332 MA cases against 144,883 controls and 8,348 MO cases against 139,622 controls, and reported that seven loci were GWS in MO but none were GWS in MA. Here, we added to the previous data 8,292 new MA and 6,707 new MO cases from headache specialist centers in Denmark and the Netherlands as well as from study collections in Iceland and UK Biobank (Table 2), for total sample sizes of 14,624 MA cases and 703,852 controls, and 15,055 MO cases and 682,301 controls. We estimated the effect size for each subtype at the 123 lead variants of the migraine GWAS (Supplementary Table 3b,c and Supplementary Data 4 and 5) and detected four GWS variants in the MA meta-analysis and 15 GWS variants in the MO meta-analysis. We also estimated a probability that the lead variant is either subtype-specific (that is, associated only with MO or with MA but not with both), shared by both subtypes, or not associated with either subtype (Methods; Supplementary Table 12a and Supplementary Data 6). With a probability above 95%, three lead variants (that is, rs12598836 in the *HMOX2* locus, rs10405121 in the *CACNA1A* locus and rs11031122 in the *MPPED2* locus) are MA-specific, while two lead variants (that is, rs7684253 in the locus near *SPINK2* and rs8087942 in the locus near *FECH*) are MO-specific at a similar threshold. Nine lead variants were shared by MA and MO with >95% probability (Fig. 3a). In addition to the five subtype-specific lead variants, four other lead variants also showed differences in effect size between the subtypes (*P* < 0.05/123) (Fig. 3b).

### Phenome-wide association scans with National Human Genome Research Institute GWAS Catalog and FinnGen R4

Next, we conducted phenome-wide association scans (PheWAS) for the lead variants for 4,314 traits with reported associations in the National Human Genome Research Institute (NHGRI) GWAS catalog (https://www.ebi.ac.uk/gwas/) and for the GWAS summary statistics of 2,263 disease traits in the FinnGen release 4 data. We identified 25 lead variants that were reported to be associated with 23 different phenotype categories (Methods) in the GWAS Catalog, and 17 lead variants with 26 defined disease categories in FinnGen at *P* < 1 × 10^−5^. The categories with the highest number of reported associations were cardiovascular disease (7 lead variants) and blood pressure (6 lead variants) in the GWAS catalog, and diseases of the circulatory system (11 lead variants) in FinnGen. When we performed PheWAS for all variants in high LD (*r*^2^ > 0.6) with the lead variants, we observed associations for 79 loci with 54 different phenotype categories in the GWAS Catalog, and for 41 loci with 26 disease categories in FinnGen (Supplementary Table 13a and Supplementary Fig. 6).

These findings are consistent with previous results that migraine is a risk factor for several cardiovascular traits^45–47^, and genetically correlated with blood pressure^48,49^. However, we did not observe a trend in the direction of the allelic effects between migraine and coronary artery disease (CAD) or migraine and blood pressure traits (Supplementary Table 13d) using the latest meta-analysis of the CARDIoGRAMplusCD4 Consortium^50^ (*n* = 336,924) and blood pressure GWAS from UK Biobank^51^ (*n* = 422,771).

### Enrichment in tissue or cell types and gene sets

We used LDSC applied to specifically expressed genes (LDSC-SEG)^14^ (Methods) to evaluate whether the polygenic migraine signal was enriched near genes that were particularly active in certain tissue or cell types as determined by gene expression or activating histone marks. Using multitissue gene expression data, we found enrichment at FDR 5% in three cardiovascular tissue/cell types, that is, aorta artery (*P* = 1.78 × 10^−4^), tibial artery (*P* = 3.60 × 10^−4^) and coronary artery (*P* = 4.29 × 10^−4^) (Table 3 and Supplementary Table 14a), all of which have previously been reported enriched in migraine without aura^14^. The fine-scale brain expression data from GTEx, since recently including 13 brain regions, showed enrichment in the caudate nucleus of striatum—a component of basal ganglia (*P* = 6.02 × 10^−4^; Table 3 and Supplementary Table 14b). With chromatin-based annotations, we found enrichment in five CNS cell types, three cardiovascular cell types, one cell type of the digestive system, one musculoskeletal/connective cell type and ovary tissue (Table 3 and Supplementary Table 14c). In addition to replicating previous findings^13,14^, the signal linking to ovary tissue has not been reported before.

**Table 3:** LDSC-SEG results that are significant at FDR 5%

Tissue/cell type and histone mark	Tissue category	*P* value	FDR adjusted *P* value
**Multitissue gene expression data**			
Aorta	Cardiovascular	1.78 × 10^–4^	0.029
Tibial artery	Cardiovascular	3.60 × 10^–4^	0.029
Coronary artery	Cardiovascular	4.29 × 10^–4^	0.029
**Gene expression data of 13 brain regions from GTEx**			
Caudate (basal ganglia)	CNS	6.00 × 10^–4^	0.008
**Multitissue chromatin annotation data**			
Fetal brain female, H3K4me3	CNS	2.49 × 10^–5^	0.012
Brain dorsolateral prefrontal cortex, H3K27ac	CNS	8.43 × 10^–5^	0.018
Brain dorsolateral prefrontal cortex, H3K4me3	CNS	1.11 × 10^–4^	0.018
Aorta, H3K4me1	Cardiovascular	2.57 × 10^–4^	0.031
Stomach mucosa, H3K36me3	Digestive	3.36 × 10^–4^	0.032
Aorta, H3K27ac	Cardiovascular	4.40 × 10^–4^	0.032
Artery-tibial ENTEX, H3K4me1	Cardiovascular	4.53 × 10^–4^	0.032
Ganglion eminence derived primary cultured neurospheres, H3K4me3	CNS	6.53 × 10^–4^	0.04
Brain germinal matrix, H3K4me3	CNS	8.42 × 10^–4^	0.043
Aorta ENTEX, H3K27ac	Cardiovascular	1.11 × 10^–3^	0.043
Artery-coronary ENTEX, H3K4me3	Cardiovascular	1.13 × 10^–3^	0.043
Cortex derived primary cultured neurospheres, H3K36me3	CNS	1.14 × 10^–3^	0.043
Ovary, H3K27ac	Other	1.15 × 10^–3^	0.043
Cortex derived primary cultured neurospheres, H3K4me3	CNS	1.29 × 10^–3^	0.045
Aorta ENTEX, H3K4me1	Cardiovascular	1.39 × 10^–3^	0.045
Stomach smooth muscle, H3K4me3	Musculoskeletal/connective	1.55 × 10^–3^	0.047

One-sided *P* value from testing whether the regression coefficient is positive. FDR, false discovery rate based on Benjamini–Hochberg method. Full results are in Supplementary Table 14a–f.

Finally, we used DEPICT^52^ to identify tissues whose eQTLs were enriched for migraine-associated variants. The tissue enrichment analysis replicated three previously reported tissues^13^: arteries (nominal *P* = 1.03 × 10^−3^), stomach (nominal *P* = 1.04 × 10^−3^) and upper gastrointestinal tract (nominal *P* = 1.29 × 10^−3^) (Supplementary Table 14a). Results of gene-set analyses using DEPICT^52^ and MAGMA^53^ are presented in Supplementary Tables 15 and 16.

## Discussion

We conducted the largest GWAS meta-analysis on migraine thus far by combining genetic data on 102,084 cases and 771,257 controls. We identified 123 migraine risk loci, of which 86 are previously unknown since the previous migraine meta-analysis, which yielded 38 loci^13^. This shows that we have now reached the statistical power for rapid accumulation of new risk loci for migraine, in line with the progress of GWAS seen with other common diseases^54^, and as expected for a highly polygenic disorder like migraine^55^.

Migraine subtypes MO and MA were defined as separate disease entities some 30 years ago, and, since then, the debate has continued as to what extent they are biologically similar. Over the years, arguments in favor^6^ and against^5^ have been presented, but convincing genetic evidence to support subtype-specific risk alleles has been lacking in genetic studies with smaller sample sizes^18,56,57^. Here, we increased considerably the evidence for subtype specificity of some risk alleles by including new migraine subtype data at the 123 migraine risk variants. We observed that, with a probability of >95%, three lead variants (in *HMOX2*, in *CACNA1A* and in *MPPED2*) are associated with MA but not MO. Of these variants, *CACNA1A* is a well-known gene linked to familial hemiplegic migraine, a rare subform of MA^58,59^. The observation that *CACNA1A* seems involved in both monogenic and polygenic forms of migraine provides a gene-based support for the increased sharing of common variants between the two disorders^55^. We find no evidence that any of the seven loci, previously reported as GWS in MO but not in MA^13^, would be specific for MO, while four of them (*LRP1*, *FHL5*, near *FGF6* and near *TRPM8*) are among the nine loci shared by both subtypes with a probability over 95%. Loci (for example, *LRP1* and *FHL5*) that are strongly associated with both subtypes provide convincing evidence for a previous hypothesis that the subtypes partly share a genetic background^13,60^. In accordance with our analysis, effects in both subtypes were suggested before at the *TRPM8* and *TSPAN2* loci, whereas, in contrast to our results, the *LRP1* locus was previously reported to be specific for MO^56^. Finally, we also detected four lead variants (including *LRP1*) that do not seem specific for MO but do confer a higher risk for MO than for MA.

It has been long debated whether migraine has a vascular or a neuronal origin, or whether it is a combination of both^8,9,61,62^. Here, we found genetic evidence for the role of both vascular and central nervous tissue types in migraine from several tissue enrichment analyses, which refined earlier analyses based on smaller sample sizes^13,14^.

With respect to a vascular involvement in the pathophysiology of migraine, both gene expression and chromatin annotation data from LDSC-SEG showed that migraine signals are enriched for genes and cell-type-specific annotations that are highly expressed in aorta and tibial and coronary arteries. The involvement of arteries was also proposed by our DEPICT tissue enrichment analysis. In addition, cardiovascular disease and blood pressure phenotypes were among the top categories in the PheWAS analyses. These results are consistent with previous reports of a shared etiology and some genetic correlation between migraine and cardiovascular and cerebrovascular endpoints^47–49,63–67^. However, in our analysis, the migraine risk alleles neither consistently increased nor consistently decreased the risk of CAD or the risk of hypertension.

A key role of the CNS in migraine pathophysiology has emerged from animal models, human imaging and neurophysiological studies^10,68^, while support for CNS involvement from genetic studies has been more difficult to obtain. A likely reason is the paucity of gene expression data from CNS tissue types, but recently more data have become available, making such studies feasible. Our LDSC-SEG analysis using gene expression data from 13 brain regions showed an enrichment for caudate nucleus in the basal ganglia, and with chromatin-based annotations for five CNS tissue types: dorsolateral prefrontal cortex, neurospheres derived from cortex, fetal brain, germinal matrix and neurospheres derived from ganglion eminence. Alterations in the structure and/or function of several brain regions^68–70^, including basal ganglia, cortex, hypothalamus, thalamus, brainstem, amygdala and cerebellum, have been reported for individuals who suffer from migraine, but the cause of these changes is not known.

In addition to the support for the hypothesis that both vascular system and CNS are important in migraine pathogenesis^8,68,71^, the tissue enrichment analyses also reported some tissue types of the digestive system as well as ovary at FDR 5%. Given the female preponderance and suggested influence of sex hormones (for example, menstrual-related migraine) in migraine^72–74^, the involvement of the ovary is an interesting finding, although the statistical evidence for it remains weaker at present compared with that for the vascular system and CNS.

A particularly interesting finding in our GWAS was the identification of risk loci containing genes that encode targets for migraine-specific therapeutics. One new locus contains the *CALCA* and *CALCB* genes on chromosome 11 that encode calcitonin gene-related peptide (CGRP). CGRP-related monoclonal antibodies have been successful for the preventive treatment of migraine^75^, and they are considered as a major breakthrough in migraine-specific treatments since the development of the triptans for acute migraine over two decades ago. Another new locus contains the *HTR1F* gene that encodes serotonin 5-HT_1F_ receptor, which is the target of another recent migraine drug class called ditans^76^. Ditans provide a promising acute treatment, especially for those migraine patients that cannot use triptans because of cardiovascular risk factors^23^. These two new GWAS associations near genes that are already targeted by effective migraine drugs suggest that there could be other potential drug targets among the new loci, and provide a clear rationale for future GWAS efforts to increase the number of loci by increasing sample sizes further. In addition, GWAS data with migraine subtype information can help prioritize treatment targets for particular migraine symptomatology, such as aura symptoms, that lack treatment options at present. More generally, using genetic evidence when selecting new drug targets is estimated to double the success rate in clinical development^77,78^.

Even though we observed links between our new risk loci and known target genes of effective migraine drugs, the accurate gene prioritization at risk loci remains challenging. First, robust fine-mapping would require accurate LD information^79^, which is typically lacking in meta-analyses and further distorted from reference panels by variation in effective sample size across variants. Second, computational approaches to gene prioritization require further methodological work^80^ and extension to additional sources of functional data to provide more robust and comprehensive gene prioritization results. Another limitation of our study is that a large proportion of migraine diagnoses are self-reported. Therefore, we cannot rule out misdiagnosis, such as, for example, tension headache being reported as migraine, which could overemphasize genetic factors related to general pain mechanisms and not migraine per se. Regardless, the high genetic correlation that we observed supports a strong phenotypic concordance between the study collections that also included deeply phenotyped clinical cohorts from headache specialist centers, which were instrumental for the migraine subtype analyses. While the subtype data provided convincing evidence of both loci with genetic differences and other loci with genetic overlap between subtypes, larger samples are still needed to achieve a more accurate picture of the similarities and differences in genetic architecture behind the subtypes.

To conclude, we report the largest GWAS meta-analysis of migraine so far, detecting 123 risk loci. We demonstrated that both the vascular system and CNS are involved in migraine pathophysiology, supporting the notion that migraine is a neurovascular disease. Our subtype analysis of migraine with aura and migraine without aura shows that these migraine subtypes have both shared risk alleles and risk alleles that seem specific to one subtype. In addition, new loci include two targets of recently developed and effective migraine treatments. Therefore, we expect that these and future GWAS data will reveal more of the heterogeneous biology of migraine and potentially point to new therapies against migraine—a leading burden for population health throughout the world.

## Methods

### Cohorts and phenotyping

All participating studies were approved by local research ethics committees, and written informed consent was obtained from all study participants. For all the participating studies, an approval was received to use the data in the present work. Study-specific ethics statements are provided in the Supplementary Note.

First, we performed a genome-wide meta-analysis on migraine including five study collections, as listed in Table 1 and Supplementary Table 1. Second, we performed subtype-specific meta-analyses on MA and on MO, both including five study collections listed in Table 2, for the 123 independent risk variants identified in the migraine analysis. A description of the study collections is given in the Supplementary Note. In particular, the migraine phenotype has been self-reported in other cohorts except in IHGC2016, where a subset of patients were phenotyped in specialized headache centers, as previously explained^13^.

### Quality control

Before the meta-analysis, a standard quality control (QC) protocol was applied to each individual GWAS. Related individuals were removed from all other cohorts except HUNT (which modeled relatedness via a logistic mixed model) by using an identity by descent cut-off of 0.185 or smaller. Multiallelic variants were excluded from all studies, and only variants that satisfied the following thresholds were kept for further analysis: minor allele frequency (MAF) > 0.01, IMPUTE2 info or MACH *r*^2^ > 0.6 and, when available, Hardy-Weinberg equilibrium (HWE) *P* value >1 × 10^−6^ and missingness <0.05. Variants were matched by chromosome, position and alleles to the UK Biobank data. Indels were recoded as insertions (I) and deletions (D). For each study, SNPs with an effect allele frequency (EAF) discrepancy of >0.30 and indels with EAF discrepancy of >0.20 to UK Biobank were excluded. MAF and EAF plots of cohorts against the reference cohort are shown in Supplementary Data 7. We conducted a sensitivity analysis on strand-ambiguous SNPs (with alleles A/T or G/C), by counting, for each pair of studies, how often the same allele of A/T or G/C SNP was coded as the minor allele in both cohorts, as a function of MAF threshold (Supplementary Table 17). Minor alleles were same at least in 97.39% of the SNPs without MAF threshold and the corresponding proportions were 99.96% and 79.58% when MAF < 0.25 and when MAF > 0.4, respectively. The very high concordance for SNPs with MAF < 0.25 suggests that the strand-ambiguous SNPs were labeled consistently for almost every SNP. Therefore, we did not exclude any SNPs based on possible labeling mismatches due to strand ambiguity.

### Statistical analysis

All statistical tests conducted were two-sided unless otherwise indicated. The GWAS for the individual study cohorts were performed by logistic regression with an additive model of imputed dosage of the effect allele on the log-odds of migraine. The analyses for IHGC2016 (ref. ^13^) and 23andMe^19^ have been described before. For UKBB data and GeneRISK data, we used PLINK v.2.0 (ref. ^81^). For HUNT data, we used a logistic mixed model with the saddlepoint approximation as implemented in SAIGE v.0.20 (ref. ^82^) that accounts for the genetic relatedness. All models were adjusted for sex and at least for the four leading principal components of the genetic population structure (Supplementary Table 18). Age was used as a covariate when available. A detailed description is provided in Supplementary Note. For the chromosome X meta-analysis, male genotypes were coded as {0,2} in all cohorts, and the GWAS were conducted with an X chromosome inactivation model that treats hemizygous males as equivalent to homozygous females^83^.

We performed an inverse–variance weighted fixed-effect meta-analysis on the five study collections by using GWAMA^84^. After the meta-analysis, we excluded the variants with effective sample size *N*_eff_ < 5,000 to remove results with very low precision compared with most variants and were left with 10,843,197 variants surpassing the QC thresholds. We estimated the effective sample size for variant *i* as

$$N_{{{{\mathrm{eff}}}}({\mathrm{i}})} = \frac{1}{{f_{\mathrm{i}}\left( {1 - f_{\mathrm{i}}} \right){\mathrm{s.e.}}_{\mathrm{i}}^2}},$$

where *f*_i_ is the effect allele frequency for variant i and s.e._i_ is the s.e. for variant i estimated by the GWAS software. This quantity approximates the value 2 *N* t(1 – t)I, where *N* is the total sample size (cases + controls), t is the proportion of cases and I is the imputation info (derivation in Supplementary Note).

### Risk loci

There were 8,117 GWS variants with the meta-analysis *P* value <5 × 10^−8^. For 8,067 of them that were available in UK Biobank, an LD matrix was obtained from UK Biobank using a random sample of 10,000 individuals included in the UKBB GWAS. We defined the index variants as the LD-independent GWS variants at LD threshold of *r*^2^ < 0.1 in the following way. First, the GWS variant with the lowest *P* value was chosen and, subsequently, all GWS variants that were in LD with the chosen variant (*r*^2^ > 0.1) were excluded. Next, out of the remaining GWS variants, the variant with the lowest *P* value was chosen and the GWS variants in LD with that variant were excluded. This procedure was repeated until there were no GWS variants left. Out of the 8,067 variants with LD information, 170 were LD-independent (at *r*^2^ < 0.1). For 18/50 variants that were not found in UK Biobank, LD information was available from the 23andMe data, and all 18 variants were in LD (*r*^2^ > 0.1) with some index variant. Of the 18 variants, 2 (rs111404218 and rs12149936) had lower *P* value than the original index variant they were in LD with and, hence, they replaced the original index variants. For 32 GWS variants, LD remained unknown. Thus, at this stage, the GWS associations were represented by 202 = 168 + 2 + 32 index variants.

Next, to define the risk loci and their lead variants, an LD block around each index variant was formed by the interval spanning all GWS variants that were in high LD (*r*^2^ > 0.6) with the index variant. Sizes of these regions ranged from 1 bp (only the variant itself, for example, the variants with unknown LD) to 1,089 kb. Sets of regions that were less than 250 kb away from each other were merged (distance from the end of the first region to the beginning of the second region). This definition resulted in 126 loci. All other GWS variants were included in their nearest locus based on their position and the locus boundaries were updated and, finally, loci within 250 kb from each other were merged. This resulted in our final list of 123 risk loci. Each risk locus was represented by its lead variant defined as the variant with the lowest *P* value and named by the nearest protein-coding gene to the lead variant or by the nearest noncoding gene if there was no protein-coding gene within 250 kb. The term ‘Near’ was added to the locus name if the lead variant did not overlap with a gene transcript. We note that the nearest gene to the lead variant need not be a causal gene. None of the 32 variants without LD information became a lead variant of a risk locus because all had a variant in the vicinity with a smaller *P* value.

We annotated and mapped these loci by their physical position to genes by using the Ensembl Variant Effect Predictor (VEP, GRCh37)^36^. We used two different thresholds for annotating the nearest genes: a distance of 20 kb and 250 kb to the nearest transcript of a gene. The filtered results including all variants within a gene or a regulatory element are presented in Supplementary Table 7b.

### Stepwise conditional analysis

We performed a stepwise conditional analysis (CA) on each risk locus by using FINEMAP v.1.4 (ref. ^85^). FINEMAP uses GWAS summary statistics together with an LD reference panel and does not require individual-level data. When the reference LD does not accurately match the GWAS data, full fine-mapping is prone to false positives^79^. A simpler stepwise CA is more robust to inaccuracy in reference LD because CA has a much smaller search space than full fine-mapping, and therefore CA is less likely to run into most problematic variant combinations where LD is very inaccurate. Since we did not have the full in-sample LD from our GWAS data, we carried out only the CA and not the full fine-mapping. For the CA, we included only the SNPs, but no indels, and we used the same reference LD from the UK Biobank data as we used to define the risk loci. We restricted the CA only to the variants with a similar effective sample size (*N*_eff_) by using a threshold of ±10% of the *N*_eff_ of the lead SNP of the risk locus, because our summary statistics came from the meta-analysis where sample sizes per variant vary greatly. This filter excluded approximately 17% of all GWS variants and was necessary since otherwise CA led to spurious conditional *P* values, such as *P* < 10^−250^, for some loci. Consequently, for two of the loci where the lead variant was an indel, the lead variant was not included in the CA. For such regions, we checked that the new lead variant from the CA output was in LD (*r*^2^ > 0.3) with the original lead variant. For one locus (rs111404218) where the lead variant does not have LD information in the UK Biobank data, there were no GWS variants left in the CA after filtering by *N*_eff_. We used the standard GWS (*P* < 5 × 10^−8^) threshold to define the secondary variants that were conditionally independent from the lead variant. The CA results are in Supplementary Tables 6a,b.

### eQTL mapping to genes and tissues

We used two data sources to map the risk variants to genes via eQTL associations. From the GTEx v.8 database (https://gtexportal.org), we downloaded the data of 49 tissues. We first mapped all 123 lead variants to all significant cis-eQTLs across tissues using the FDR cut-off of 5% as provided by the GTEx project^39^. Next, we also mapped the variants in high LD (*r*^2^ > 0.6) with the lead variants to all significant cis-eQTLs. Finally, we filtered the results to include only the new significant gene-tissue pairs that were not implicated by the lead variants. Results are shown in Supplementary Tables 9 and 10.

With FUMA v.1.3.6 (ref. ^40^), we mapped the 123 lead variants, and the variants in high LD (*r*^2^ > 0.6) with the lead variants, to the other eQTL data repositories provided by FUMA except GTEx, that is, Blood eQTL Browser^86^, BIOS QTL browser^87^, BRAINEAC^88^, MuTHER^89^, xQTLServer^90^, CommonMind Consortium^91^, eQTLGen^92^, eQTL Cataloque^93^, DICE^94^, scRNA eQTLs^95^ and PsychENCODE^96^. Results are shown in Supplementary Tables 9 and 10.

To study whether the lead variants were enriched in any of the 49 tissues from GTEx v.8, we fitted a linear regression model where the number of lead variants that are significant cis-eQTLs for a specific tissue was used as the outcome, and the overall number of genes with at least one significant cis-eQTL reported by GTEx for the tissue was the predictor^39^. We did a separate regression model for each tissue type by leaving the tissue of interest out from the model, and we used the model fitted on the other tissues for predicting the outcome variable for the tissue type of interest. Finally, we checked in which tissues the true observed number of migraine lead variants was outside of the 95% prediction intervals as given by the function ‘predict.lm(‘interval=’prediction’)’ in R software. Details of the procedure are in the Supplementary Note.

### LD-score regression

We estimated both the SNP heritability ($h_{\mathrm{SNP}}^2$) of migraine and pairwise genetic correlations (*r*_G_) between each pair of study collections using LDSC v.1.0.0 (refs. ^30,35^). SNP heritability and genetic correlations were estimated using European LD scores from the 1000 Genomes Project Phase 3 data for the HapMap3 SNPs, downloaded from https://data.broadinstitute.org/alkesgroup/LDSCORE/. We reformatted the meta-analysis association statistics to LDSC format with munge-tool, which excluded variants that did not match with the HapMap3 SNPs, had strand ambiguity (that is, A/T or G/C SNPs), MAF <0.01 or missingness more than two-thirds of the 90th percentile of the total sample size, or resided in long-range LD regions^97^, in centromere regions or in the major histocompatibility locus (MHC) of chromosome 6, leaving 1,165,201 SNPs for the LDSC analyses. We used a migraine population prevalence of 16% and a sample proportion of cases of 11.7% = 102,084/(102,084 + 771,257) to turn the LDSC slope into the estimate of $h_{\mathrm{SNP}}^2$ on the liability scale^98^. Pairwise genetic correlation results are listed in Supplementary Table 2. We note that in the previous migraine meta-analysis^13^, LDSC reported $h_{\mathrm{SNP}}^2$ value of 14.6% (13.8–15.5%), which was considerably larger than the value 11.2% (10.8–11.6%) that we report in our analysis. When we ran our LDSC pipeline on the data of Gormley et al.^13^, we estimated $h_{\mathrm{SNP}}^2$ value of 10.6% (10.1–11.1%). Thus, it seems that our liability transformation estimates lower values of heritability than the transformation used by Gormley et al.^13^.

### Stratified LD-score regression

We used S-LDSC to partition the SNP heritability by functional genomic annotations^37^. We used the baseline-LD model^38^ that contains 75 annotations, including conserved, coding and regulatory regions of the genome and different histone modifications. The baseline-LD model adjusts for MAF- and LD-related annotations, such as recombination rate and predicted allele age, which decreases the risk of model misspecification^37,38,99^. We used the same QC as with the univariate LDSC, and the baseline LDv.1.1 European LD scores estimated from the 1000 Genomes Project Phase 3, downloaded from https://data.broadinstitute.org/alkesgroup/LDSCORE/. We set the significance threshold for enrichment of individual binary functional annotations to *α* = 0.05/24, as we considered only 24 unique functional annotations without the flanking regions. Results are listed in Supplementary Table 8.

### Subtype analyses of MA and MO

First, we combined new MA and MO data (Table 2) with the previously used migraine subtype-specific meta-analysis data^13^, and estimated migraine subtype-specific effect sizes for the 123 lead variants from the migraine meta-analysis. We tested how often the direction of allelic effects was similar between the IHGC MA/MO and the new cohorts using a binomial test (Supplementary Table 12b). Next, we stratified the lead variants by using the information from the migraine subtype-specific analyses. For each of the variants, we estimated probabilities between four possible explanations of the observed data that we call ‘NULL’, ‘MO’, ‘MA’ and ‘BOTH’. Under model NULL, the effect is not present in either of the migraine subtypes (that is, the effect is zero); under model MO or MA, the effect is present only in MO or only in MA but not in both; and under model BOTH, a nonzero effect is shared by both MO and MA. We used a Bayesian approach for model comparison that combines a bivariate Gaussian prior distribution on the two effect sizes with a bivariate Gaussian approximation to the likelihood using GWAS summary statistics^100^. Across all models, the prior s.d. for the effect is 0.2 on the log-odds scale for nonzero effects and 0 for a zero effect. The bivariate priors for the four models are as follows: NULL assumes a zero effect in both migraine subtypes, MO and MA assume a nonzero effect for one subtype and a zero effect for the other subtype, and BOTH combines the fixed-effect model (exactly the same effect in both subtypes) with the independent-effects model (the two effect sizes are nonzero but uncorrelated with each other) with equal weights. Finally, we assumed that each of the four models (NULL, MO, MA and BOTH) is equally probable a priori, which we considered an appropriate assumption since all these variants show a convincing association to overall migraine (*P* < 5 × 10^−8^). Then we used the Bayes formula to work out the posterior probability on each model. The results are shown in Fig. 3a, thresholded by a probability cut-off of 95%, and in Supplementary Table 12a. The correlation parameter between MO and MA GWAS statistics needed in the bivariate likelihood approximation was estimated to be 0.148 using the empirical Pearson correlation of the effect size estimates of the common variants (MAF > 0.05), which did not show a strong association to either of the migraine subtypes (*P* > 1 × 10^−4^)^101^. We tested whether the effect sizes between MA and MO were equal at a Bonferroni corrected significance threshold of *α* = 0.05/123 by using a normal approximation and accounting for the correlation in effect size estimators.

We note that the amount of information in the data (‘statistical power’) is taken into account automatically in this model comparison, which we consider an advantage compared with a comparison of the raw *P* values between the subtype analyses that does not automatically account for statistical power. In particular, observing a GWS *P* value (*P* < 5 × 10^−8^) in one subtype but not in the other subtype is not yet evidence for a subtype-specific locus, because the effect could still be nonzero also for the other subtype but simply lack power to reach the stringent GWS threshold. Finally, we point out that the inference in the model comparison approach is conditional on the particular set of models being included in the comparison, as well as on the particular choice of the prior distributions.

### PheWAS with NHGRI GWAS catalog and FinnGen R4

We performed PheWAS for the 123 lead variants using the NHGRI GWAS catalog and the FinnGen R4 GWAS summary statistics. In addition, we performed the same lookups for the 123 risk loci including all variants in high LD (*r*^2^ > 0.6) with the lead variants. With the GWAS catalog, we first downloaded all the available results (4,314 traits) from the GWAS catalog webpage (accessed April 6, 2020). Next, we obtained all the associations for the 123 risk loci with all the high LD variants included using *P* value thresholds of *P* < 1 × 10^−5^, *P* < 1 × 10^−6^ and *P* < 1 × 10^−4^ (Supplementary Table 13a–c). Because the GWAS catalog includes results from several different GWAS for the same phenotype or for a very similar phenotype with a different name, we divided the phenotype associations into broader categories. The new categories are listed in Supplementary Table 19. The same approach was used for the PheWAS of FinnGen R4. We first downloaded all the available summary statistics (2,263 endpoints) and, next, obtained all the associations for the 123 risk loci using the same three *P* value thresholds as with the GWAS catalog (Supplementary Table 13a–c). We also divided similar endpoints into broader categories, which are listed in Supplementary Table 20.

We tested the direction of allelic effects between migraine and the following three traits that shared multiple associated variants with migraine: CAD^50^, diastolic blood pressure^51^ and systolic blood pressure^51^. We first took all migraine lead variants that were available also in the summary statistics of the other trait without any *P* value threshold and used a binomial test to test whether the proportion of variants with same direction of effects was 0.5. Next, we used a *P* value threshold of 1 × 10^−5^ for the association with the other trait. Results are in Supplementary Table 13d.

### LD-score regression applied to specifically expressed genes

We used LDSC-SEG^14^ to identify tissues and cell types implicated by the migraine GWAS results. LDSC-SEG uses gene expression data and GWAS results from all variants together with an LD reference panel. For our analyses, we used the same QC as for the other LDSC analyses and six different sets of readily constructed annotation-specific LD scores downloaded from https://data.broadinstitute.org/alkesgroup/LDSCORE/LDSC_SEG_ldscores/: multitissue gene expression, multitissue chromatin, GTEx brain, Cahoy, Corces ATAC and ImmGen LD Scores. FDR was controlled by the Benjamini–Hochberg method. The results are in Supplementary Table 14a–f. There were no significant results with the Cahoy, Corces ATAC and ImmGen data at FDR 5%.

### Multimarker analysis of genomic annotation

We applied multimarker analysis of genomic annotation (MAGMA) v.1.09 (ref. ^53^) to identify genes and gene sets associated with the migraine meta-analysis results. First, we mapped the meta-analysis SNPs to 18,985 protein-coding genes based on their physical position in the National Center for Biotechnology Information 37 build by using default settings of MAGMA. Next, we performed a gene-based analysis using the default SNPwise-mean model and the same UK Biobank LD reference as for the other analyses. We applied a Bonferroni correction (*α* = 0.05/18,985) to identify significantly associated genes for migraine with the results listed in Supplementary Table 16a. Finally, we used the results from the gene-based analysis to perform a gene-set analysis by using two different gene-set collections from the Molecular Signature Database v.7.0 (refs. ^102,103^): the curated gene sets containing 5,500 gene sets and the GO gene sets containing 9,988 gene sets. We performed the gene-set analysis using the competitive gene-set model and one-sided test that tests whether the genes in the gene-set are associated more strongly with the phenotype compared to the other genes. To correct for multiple testing, we used a Bonferroni correction (*α* = 0.05/(5,500 + 9,988)). Results are in Supplementary Table 16b,c and in Supplementary Fig. 7.

### DEPICT

DEPICT^52^ is an integrative tool to identify the most likely causal genes at associated loci, and enriched pathways and tissues or cell types in which the genes from the associated loci are highly expressed. As an input, DEPICT takes a set of trait-associated SNPs. First, DEPICT uses coregulation data from 77,840 microarrays to predict biological functions of genes and to construct 14,461 reconstituted gene sets. Next, information of similar predicted gene functions is used to identify and prioritize gene sets that are enriched for genes in the associated loci. For the tissue- and cell-type-enrichment analysis, DEPICT uses a set of 37,427 human gene expression microarrays. We used DEPICT v.1.194 and ran the analyses twice for each of the *P* value thresholds for clumping, as recommended^52^, and using the default settings of 500 permutations for bias adjustment and 50 replications for the FDR estimation and for the *P* value calculation. As an input, we used only the autosomal SNPs and the same UK Biobank LD reference data as for the other analyses. First, we ran the analysis using a clumping *P* value threshold of 5 × 10^−8^ that resulted in 165 clumps formed from 7,672 variants (Supplementary Table 15d–f). Second, we used a *P* value threshold of 1 × 10^−5^ leading to 612 clumps formed from 22,480 variants (Supplementary Table 15a–c).

### Transcriptome-wide association study and colocalization

We performed a transcriptome-wide association study (TWAS) by S-PrediXcan^42^ v.0.7.5 using GTEx v.8 multivariate adaptive shrinkage models (MASHR-M) for 49 tissues downloaded from predictdb.org and the European 1000 Genomes v.3 LD reference panel (hg38; https://zenodo.org/record/3657902/). We followed the recommended QC protocol, and first harmonized and imputed the migraine summary statistics to ensure an optimal overlap with the GTEx v.8 expression weights. After harmonization and summary statistic imputation, 8,909,736 variants were available for the TWAS. We performed the analysis with default settings to identify significant gene-tissue pairs. We applied a Bonferroni corrected significance level of *α* = 0.05/662,726, corresponding to the number of unique gene-tissue pairs tested.

Next, we performed colocalization analysis with COLOCv.4.0.4(ref. ^43^) R package for the 1,844 significant gene-tissue pairs to indicate pairs that could be due to LD contamination. COLOC compares five hypotheses where the null hypothesis (H0) corresponds to no association to either eQTL or GWAS, H1 and H2 correspond to associations with only one of the traits, H3 corresponds to association with both eQTL and GWAS but at distinct causal variants, and H4 corresponds to association with both eQTL and GWAS at a shared causal variant. We set a prior probability for colocalization as p_12_ = 5 × 10^−6^ for all tested regions and restricted the analysis to variants that had *N*_eff_ ± 10% of the *N*_eff_ of the lead variant of the region. Results are presented in Supplementary Table 11b.

### Fine-mapping of causal gene sets

To prioritize genes for the migraine loci, we applied a gene-based fine-mapping approach using fine-mapping of causal gene sets (FOCUS) v.0.7 (ref. ^41^). FOCUS is a Bayesian approach that models predicted expression correlations among TWAS signals to estimate posterior probabilities for all genes within a tested region.

We used the European 1000 Genomes v.3 LD reference panel and same GTEx v.8 predicted expression weights for the 49 tissues as with S-PrediXcan. First, we mapped the migraine summary statistics from hg37 to hg38 with UCSC liftOver^104^. Next, we followed the suggested QC protocol and applied the modified munge-tool to obtain cleaned summary statistics. After the QC steps, we had 6,237,177 variants left for the analysis. We performed tissue-prioritized fine-mapping of gene-sets for the 49 tissues with otherwise default settings except that we increased the *P* value threshold to 1 × 10^−4^ so that the fine-mapping would cover most of the same regions that contained at least one significant gene-tissue pair by S-PrediXcan. Posterior inclusion probability (PIP) from FOCUS is reported for all available significant S-PrediXcan gene-tissue pairs in Supplementary Table 11b, and all prioritized genes by FOCUS with PIP >0.9 are reported in Supplementary Table 11a.

### Reporting Summary

Further information on research design is available in the Nature Research Reporting Summary linked to this article.

## Online content

Any methods, additional references, Nature Research reporting summaries, source data, extended data, supplementary information, acknowledgements, peer review information; details of author contributions and competing interests; and statements of data and code availability are available at 10.1038/s41588-021-00990-0.

## Supplementary information

Supplementary Information Supplementary Note and Figs. 1–8.

Reporting Summary.

Peer Review Information.

Supplementary Table 1 Supplementary Tables 1–20.

Supplementary Data 1 Regional LocusZoom plots of the 123 independent migraine risk loci identified from the meta-analysis.

Supplementary Data 2 Forest plots of the 123 lead migraine variants.

Supplementary Data 3 Forest plots of 10 variants that have been previously reported to associate with migraine but failed to replicate in our study.

Supplementary Data 4 Forest plots of the 123 lead migraine variants from the MO meta-analysis.

Supplementary Data 5 Forest plots of the 123 lead migraine variants from the MA meta-analysis.

Supplementary Data 6 Subtype-specific combined log-odds-ratio estimates and posterior probabilities from subtype-specificity analysis for the 123 lead migraine variants.

Supplementary Data 7 Pairwise EAF and MAF plots against the reference cohort (UKBB).

## Data Availability

Results for 8,117 genome-wide significant SNP associations (*P* < 5 × 10^−8^) from the meta-analysis including 23andMe data are available on the International Headache Genetics Consortium website (http://www.headachegenetics.org/content/datasets-and-cohorts). Genome-wide summary statistics for the other study collections except 23andMe are available for bona fide researchers (contact Dale Nyholt, d.nyholt@qut.edu.au) within 2 weeks from the request. The full GWAS summary statistics for the 23andMe discovery data set will be made available through 23andMe to qualified researchers under an agreement with 23andMe that protects the privacy of the 23andMe participants. Please visit https://research.23andme.com/collaborate/#publication for more information and to apply to access the data.

## Code availability

R code for the subtype specificity analysis: https://github.com/mjpirinen/migraine-meta.
