# Supplementary material for: Genome-wide analysis of 102,084 migraine cases identifies 123 risk loci and subtype-specific risk alleles
Source: Nat Genet. 2022 Feb 3;54(2):152–60. doi: 10.1038/s41588-021-00990-0 (PMC8837554; doi:10.1038/s41588-021-00990-0)
Supplement: Supplementary file 1 — Supplementary Note and Figs. 1–8. [file 41588_2021_990_MOESM1_ESM.pdf]

nature portfolio

https://doi.org/10.1038/s41588-021-00990-0

Supplementary information

Genome-wide analysis of 102,084 migraine cases identifies 123 risk loci and subtype-specific risk alleles

In the format provided by the authors and unedited

Supplementary Note

Table of Contents

Study descriptions **1**
23andMe 2
GeneRISK 2
HUNT All-in Headache 3
IHGC2016 5
UKBB 5
DBDS 6
deCODE 7
LUMINA 8
Study-specific acknowledgements **11**
Ethics statement **12**
Derivation of effective sample size N_{eff} **12**
Enrichment of migraine associated variants for the GTEx v8 tissues **13**
Consortia **14**
International Headache Genetics Consortium 14
HUNT All-in Headache 16
Danish Blood Donor Study Genomic Cohort 17
Supplementary References **18**
Supplementary Figures **20**

Study descriptions

Our primary genome-wide meta-analysis on migraine included five study collections: IHGC2016, 23andMe, UKBB, GeneRISK and HUNT All-in Headache. In addition, we performed subtype-specific analyses for migraine with aura (MA) and migraine without aura (MO) for the 123 lead variants of the migraine risk loci identified in the primary meta-analysis by using data from five study collections: IHGC2016, UKBB, deCODE, DBDS, and LUMINA. All these study collections are described below.

23andMe

23andMe cohort includes 220,876 controls and 53,109 migraine cases, of which 30,465 were included in the previous meta-analysis of Gormley et al. (2016) and 22,644 are new cases. 23andMe migraine GWAS was performed by a personal genomics company 23andMe, Inc. (https://www.23andme.com/) and detailed description of the migraine GWAS is provided elsewhere[1]. All participants have provided informed consent and filled an online survey according to 23andMe's human subjects protocol, which was reviewed and approved by Ethical & Independent Review Services, a private institutional review board. Briefly, migraine cases were assessed from the participants that had reported migraine or answered "yes" to any of the questions related to migraine, and controls from participants that did not report having migraine or answered "No" to all of the questions related to migraine, excluding participants with discordant answers. The GWAS was conducted for a set of unrelated individuals with European ancestry by logistic regression assuming additive allelic effects and controlled for age, sex and five principal components of genetic ancestry. The relatedness exclusion was based on an analysis of a segmental identity-by-descent algorithm, and the cut-off was chosen to be the minimal expected amount of sharing between first cousins. Besides the variant QC reported in[1], we excluded also duplicated variants and variants with MAF ≤ 0.01 or average Minimac2 $r^2 \leq 0.6$.

GeneRISK

GeneRISK cohort includes 1,084 migraine cases and 4,857 controls from the GeneRISK Study. The GeneRISK Study is a prospective observational study that focuses on genetic risk factors of cardiovascular diseases. Participants for the recruitment were randomly selected from 45 to 65 years old individuals from Southern Finland, and the data were collected during 2015-2018. Exclusion criteria for the participation were diagnosed stroke, myocardial infarction, cerebral hemorrhage, cerebral infarction, coronary heart disease, or if the participant had undergone coronary artery bypass surgery or coronary angioplasty, or if the participant was pregnant, or had a guardian of interest. All study participants attended to a clinical health check-up and filled an electronic questionnaire that included several topics related to their health status and lifestyle factors. Migraine cases were assessed from participants who answered "Yes" to the question of "Have you been diagnosed migraine by doctor? Yes/No", and controls from participants who answered "No". The samples were genotyped by using Illumina HumanCoreExome arrays (HumanCoreExome24 v1.0 or HumanCoreExome24 v1.1) and ancestry were inferred by principal component analysis using PLINK v2.0. Genotype data were imputed using a population specific

reference panel SISu v3 with Beagle 4.1 version 08Jun17.d8b[2] as described here: dx.doi.org/10.17504/protocols.io.nmndc5e. Related individuals were removed using a threshold of IBD > 0.1 such that individuals who had many relatives were preferred for exclusion. Genetic analysis was run by PLINK v2.0 by applying logistic regression with firth-fallback -mode and controlling for sex, age and first 10 principal components of genetic ancestry. For the meta-analysis, we included only biallelic variants with MAF > 0.01, INFO > 0.6, HWE P-value > 1×10^{-6} and missingness < 0.05

All study participants have given their informed consent, and the study protocol has been approved by the Ethical Committee of the Helsinki and Uusimaa Hospital district.

HUNT All-in Headache

Sample ascertainment and phenotype definition. The Trøndelag Health Study (HUNT) consists of three different population-based health surveys conducted in the county of Nord-Trøndelag, Norway over approximately 20 years (HUNT1 [1984-1986], HUNT2 [1995-1997] and HUNT3 [2006-2008])[3]. At each survey, the entire adult population (≥ 20 years) was invited to participate by completing questionnaires, attending clinical examinations and interviews. Participation rates in HUNT1, HUNT2 and HUNT3 were 89.4% (n=77,212), 69.5% (n=65,237) and 54.1% (n=50,807), respectively[3]. Taken together, the study included more than 120,000 different individuals from Nord-Trøndelag County. Biological samples including DNA have been collected for approximately 70,000 participants. The HUNT Study has been described in more detail elsewhere[3]. For the present study, we included participants from HUNT2 and HUNT3. Migraine was assessed using questionnaires, and based on a modified version of the International Classification of Headache Disorders (ICHD II)[4]. Subjects who answered “yes” to the question “Have you suffered from headache during the last 12 months?” were classified as headache sufferers. Those who answered “no” in one or both studies, and “yes” in neither, constitute the control group. Based on the subsequent headache questions[5,6] headache sufferers were classified as having migraine if they fulfilled the following 3 criteria: (1) headache attacks lasting 4 to 72 hours, (< 4 hours was accepted for those who reported commonly occurring visual disturbances before headache); (2) headache with at least one of the following characteristics: pulsating quality, unilateral location, or aggravation by physical activity; (3) during headache, at least one of the following occurred: nausea, photophobia and phonophobia. In addition, the participants were asked if they suffered from migraine; those who responded positively to this question were also included in the migraine

group. For the present study, subjects were classified as having migraine if they fulfilled the criteria for migraine in either HUNT2 or HUNT3. The migraine diagnoses have been validated by clinical interviews performed by neurologists. In HUNT2, the sensitivity was 69% and specificity 89% (κ= 0.59, 95% CI 0.47–0.71)[5]. In HUNT3 the sensitivity was 49% and specificity 96% (κ = 0.51, 95% CI 0.34–0.68)[6] .

Genotyping, quality control and imputation. In total, DNA from 71,860 HUNT samples was genotyped using one of three different Illumina HumanCoreExome arrays (HumanCoreExome12 v1.0, HumanCoreExome12 v1.1 and UM HUNT Biobank v1.0). Samples that failed to reach a 99% call rate, had contamination > 2.5% as estimated with BAF Regress[7], large chromosomal copy number variants, lower call rate of a technical duplicate pair and twins, gonosomal constellations other than XX and XY, or whose inferred sex contradicted the reported gender, were excluded. Samples that passed quality control were analyzed in a second round of genotype calling following the Genome Studio quality control protocol described elsewhere[8]. Genomic position, strand orientation and the reference allele of genotyped variants were determined by aligning their probe sequences against the human genome (Genome Reference Consortium Human genome build 37 and revised Cambridge Reference Sequence of the human mitochondrial DNA; http://genome.ucsc.edu) using BLAT[9]. PLINK v1.90[10] was then used to exclude variants if their probe sequences could not be perfectly mapped, cluster separation was < 0.3, Gentrain score < 0.15, showed deviations from Hardy Weinberg equilibrium in unrelated samples of European ancestry with *P*-value < 0.0001), had a call rate < 99%, or another assay with higher call rate genotyped the same variant. Ancestry of all samples was inferred by projecting all genotyped samples into the space of the principal components of the Human Genome Diversity Project (HGDP) reference panel (938 unrelated individuals; downloaded from http://csg.sph.umich.edu/chaolong/LASER/)[11,12] using PLINK. Recent European ancestry was defined as samples that fell into an ellipsoid spanning exclusively European populations of the HGDP panel. The different arrays were harmonized by reducing to a set of overlapping variants and excluding variants that showed frequency differences > 15% between data sets, or that were monomorphic in one and had MAF > 1% in another data set. The resulting genotype data were phased using Eagle2 v2.3 47.

Imputation was performed on the 69,716 samples of recent European ancestry using Minimac3 (v2.0.1, http://genome.sph.umich.edu/wiki/Minimac3)[13] with default settings (2.5 Mb reference based chunking with 500kb windows) and a customized Haplotype Reference consortium release

1.1 (HRC v1.1) for autosomal variants and HRC v1.1 for chromosome X variants[14]. The customized reference panel represented the merged panel of two reciprocally imputed reference panels: (1) 2,201 low-coverage whole-genome sequences samples from the HUNT study and (2) HRC v1.1 with 1,023 HUNT WGS samples removed before merging. We excluded imputed variants with $r^2 <$ 0.3 resulting in over 24.9 million well-imputed variants. After restricting to those with available phenotype information, and after excluding 2,697 HUNT participants that were genotyped and included in a previous GWAS meta-analysis of migraine[15], 40,224 individuals (7,801 cases and 32,423 controls) were included in the analysis.

Association analysis. Association analyses were conducted using SAIGE v0.20[16], a logistic mixed effects model approach, to account for cryptic population structure and relatedness when modelling the association between genotype probabilities (dosages) and binary migraine phenotype. Models were adjusted for sex, age, genotyping batch and four principal components (PCs). PCs were computed using PLINK. Additional filters applied to the analysis included minor allele count ≥ 1 and imputation $r^2 \geq 0.3$. For the meta-analysis, we included only biallelic variants with MAF > 0.01, imputation $r^2 > 0.6$ and HWE P-value > 1×10^{-6}.

Written informed consent was obtained from all participants, and The Regional Committee for Medical and Health Research Ethics approved the study (ref. 2015/576).

IHGC2016

IHGC2016 study collection includes 21 cohorts from[15] meta-analysis excluding 23andMe cohort. IHGC2016 MA includes 12 cohorts, and IHGC2016 MO includes 11 cohorts. A detailed cohort and phenotype description of the included 21 cohorts are provided in the supplementary material of[15].

UKBB

UKBB cohort includes 10,881 migraine cases and 330,170 controls from UK Biobank. UKBB MA cohort includes 1,333 MA cases and 320,139 controls, and UKBB MO cohort includes 187 MO cases and 320,139 controls from UK Biobank. The UK Biobank project is a population-based prospective cohort study that consist of over 500,000 participants aged 40-69 at recruitment collected from several regions across the United Kingdom. Participants completed questionnaires and attended interviews and clinal examinations by a trained staff member. Detailed description of UK Biobank is provided elsewhere[17], and all detailed genotyping, quality control and imputation

procedures are described at the UK Biobank website (https://www.ukbiobank.ac.uk/). Migraine cases were selected from the self-reported non-cancer illness code (Data field 20002, code 1265). This field includes participants who had self-reported migraine on the questionnaire and participants who had been uncertain about the illness they had had, but have been classified as having migraine by a trained nurse during the verbal interview after describing their symptoms. The control group was selected from the participants without self-reported migraine and without self-reported headache (Data field 20002, code 1436). MA and MO cases were compiled from the following data fields: GP clinical event records (42040, codes 14740, F260., .F351, X007J, X007K, X007N, XaXkv, F26y0, X007L, X007M for MA, and code F261.. for MO), ICD-10 main summary diagnoses (41270, code G431 for MA and code G430 for MO) and ICD-9 main summary diagnoses (41203, code 3460 for MA and code 3461 for MO). Shared control group for MA and MO were selected from the participants who did not have any of the migraine diagnoses listed above, other migraine diagnosis in the Data Field 42040 or self-reported migraine or headache in Data field 20002. We restricted the sample to include only individuals who were of European ancestry and were classified as 'White British'[17], and we excluded related individuals using a kinship value of 0.0442 corresponding to excluding 3rd degree relatives. The kinship values were obtained from UK Biobank and had been computed using KING [18]. We ran the GWAS by PLINK v2.0 by applying logistic regression with firth-fallback -mode and controlling for sex, age and first 10 principal components of genetic ancestry. We included only biallelic variants with MAF > 0.01, INFO > 0.6, HWE P-value > 1×10^{-6} and missingness < 0.05.

UK Biobank received ethical approval from the North West Multi-centre Research Ethics Committee (MREC) and informed consent has been obtained from participants.

DBDS

The DBDS Genomic Cohort contains 7,694 migraine cases (3,756 migraine without aura and 3,938 migraine with aura) and 28,045 controls from the Danish Blood Donor Study. The DBDS is a population-based study that consists of over 100K blood donors comprising extensive phenotype data based on questionnaires and whole genome genotyping data[19]. Migraine diagnosis was based on a self-reported migraine-questionnaire enabling diagnosis of migraine with and without aura in accordance with the ICHD 3 criteria. Genotyping was performed at deCODE genetics using the Global Screening Array by Illumina and imputed using a pan-Scandinavian whole genome sequencing sample of size > 8, 000 as reference including > 6,000 samples of Danish descent. Non-

European participants were excluded based on principal component analysis comparing to 1000G data. Related individuals were excluded based on proportion IBD of 0.125. Genetic variants were removed using PLINK v2.0 based on MAF < 0.05, HWE *P*-value < $1x10^{-5}$, missingness per variant > 0.05 and per individual > 0.1. Of the 123 independent risk loci, 104 were present in the DBDS data. The 104 loci were tested for association with MO and MA using PLINK v2.0 by applying logistic regression (MO vs controls, and MA vs controls) and controlling for sex, age and the first ten principal components.

Written informed consent was obtained from all participants in DBDS. DBDS has secured necessary permissions and approval from the Danish Data Protection Agency (2007-58-0015) and the Scientific Ethical Committee system (M-20090237).

deCODE

The deCODE cohorts of migraine without aura (MO, N = 1,736) and migraine with aura (MA, N = 3,376) were established from participants in an ongoing study on the genetics of migraine in Iceland who were (A) diagnosed by neurologists (with ICD10 G43.0 (MO) or ICD10 G43.1 (MA), respectively), (B) or identified through a survey of the larger deCODE migraine cohort that also includes unsubtyped migraine cases (ICD10 G43), migraineurs diagnosed by primary care physicians (ICD10 G43 or ICPC-2 N89), cases identified through prescription data from the National Drug Database (with at least two prescriptions for anti-migraine drugs, Triptans) and cases defined by answers to the third edition of the deCODE Migraine Questionnaire (DMQ3) designed for use in genetic studies[20]. From these, we included in the MO/MA case groups individuals who responded "yes" to either of recent (2020) survey questions "Has a physician diagnosed you with migraine without aura? " or "Has a physician diagnosed you with migraine with aura?". For both MO and MA, we compared cases to population controls, excluding from controls all other migraineurs as defined above (in B).

The MO/MA GWAS were performed using 37.6 million high-quality sequence variants by whole-genome sequencing of Icelanders by methods that have been described in detail elsewhere[21,22]. In short, the 37.6 million variants were identified by sequencing 49,962 Icelanders using GAIIx, HiSeq, HiSeqX, and NovaSeq Illumina technology to a mean depth of at least 17.8×. SNPs and indels were identified and their genotypes called using joint calling with Graphtyper[23]. Additionally, over 165,000 Icelanders (including all sequenced Icelanders) have been genotyped using various Illumina SNP chips and phased using long-range phasing[24], which allows for improving genotype

calls using the information about haplotype sharing. The genotypes of the high-quality sequence variants were imputed into the chip-typed Icelanders[25]. To increase the sample size and power to detect associations, the sequence variants were also imputed into relatives of the chip-typed using genealogic information. All tested variants had imputation information over 0.8. Association analyses were performed using LD score regression to account for distribution inflation due to cryptic relatedness and population stratification. They were also adjusted for gender, age, county of origin, current age or age at death (first and second order term included), blood sample availability for the individual, and an indicator function for the overlap of the lifetime of the individual with the time span of phenotype collection.
Approval for these studies was provided by the National Bioethics Committee (approval no. VSN 19-158). All participants who donated blood signed informed consent. The personal identities of participants were encrypted using a third-party system approved and monitored by the Icelandic Data Protection Authority.

LUMINA

Selection of Dutch cases from LUMINA and controls from NEO.

For the follow-up association study to validate the 123 top SNPs of the meta-analysis, additional migraine cases, that is the Dutch MO and Dutch MA studies described below, were recruited and genotyped as part of the Leiden University Migraine Neuro Analysis (LUMINA) cohort[15]. The Dutch MO study contains 1,220 Dutch MO patients that were recruited through our specialized headache clinic. After genotyping and standard GWA study quality control procedures 1,116 cases remained for the analysis. Of the 1,116 Dutch MO patients, 136 (12.2%) were male and 980 (87.8%) were female. The Dutch MA study contains 766 Dutch MA patients that were recruited through our specialized headache clinic. After genotyping and standard GWA study quality control procedures, 724 cases remained for the analysis. Of the 724 MA cases, 130 (18.2%) were male and 594 (82.0%) were female.
Self-reported migraineurs were recruited via the project's website (www.lumc.nl/hoofdpijn). A set of screening questions validated previously in a population-based study was used[26]. Participants fulfilling the screening criteria then completed an extended questionnaire that focuses on signs and symptoms of migraine headache and aura (aura symptoms were absent in the selected MO patient group) as outlined in formerly ICHD-II, now ICHD-III[4, 27]. Individual diagnoses were made using an algorithm based on these criteria, validated by a semi-structured telephone interview performed by experienced physicians or by well-trained medical students, when necessary in consultation with

a neurologist specialized in headache (GMT)[28]. A subset of patients was asked to participate upon visiting our outpatient clinic. The LUMINA project was approved by the medical ethics committee of the Leiden University Medical Center. All respondents provided written informed consent.

For the association study to validate the 123 top SNPs of the meta-analysis, population-matched controls were obtained from the Netherlands Epidemiology of Obesity Study (NEO) (N = 5,644)[29]. A subset of this cohort (N = 1,671 before QC) not selected on any clinical parameter (except age that ranged from 45 to 65 years) living in a nearby municipality (Leiderdorp, The Netherlands) was used for the final analysis. Written informed consent was obtained from all participants, and the local ethics committee approved the study.

Genotyping, quality control, and imputation: Genomic DNA was extracted from peripheral blood leukocytes according to standard protocols and genotyping of both cases and controls was performed on InfiniumCoreExome-24v1-1_A beadchip. Cases were genotyped at FIMM Technology Centre (Helsinki, Finland) and controls at the Centre National de Génotypage (Paris, France). For the cases, variant calling was performed with Genome Studio v.2011.1 following a standard quality protocol. For quality control (QC), markers with high missingness rates (≥ 2%), and those failing the Hardy-Weinberg equilibrium were excluded. Individuals were excluded if they had a high proportion of missing genotype data (≥ 2%), inconsistent sex information, were related (PI-HAT ≥ 0.2), were ancestry outliers, or heterozygosity outliers. Principal component analysis (PCA) was performed on the pruned data set (with a 50-kb sliding window, $r^2 > 0.2$) using PLINK and population outliers were excluded. After combining the genotyped SNP information from cases and controls imputation was performed on the Michigan Imputation Server using Haplotype Reference Consortium (HRC v1.1 2016) as a reference panel after phasing by Eagle (v2.3)[13,,30] using the default parameters.

For the MO dataset a total of 239,606 SNPs from 6,794 individuals (1,150 cases and 5,644 controls) was available for imputation. All cases (N = 1,116) and the NEO Leiderdorp control subset (N = 1,445; 630 male and 815 female), whom had passed previous QC steps were extracted for analysis. The analysis was based on a total of 16,180,654 SNPs.

For the MA dataset a total of 316,010 SNPs from 6,394 individuals (741 cases and 5,653 controls) were available for imputation. All cases (N = 724) and the NEO Leiderdorp control subset (N = 1,447; 629 male and 818 female), whom had passed previous QC steps were extracted for analysis. The analysis was based on a total of 15,336,357 SNPs.

For both the MO and the MA sample an association analysis was performed using a logistic regression model implemented in SNPTEST (version 2.5.2) for autosomal variants[31] with case-

control status as outcome and assuming additive allelic effects. The model was adjusted for sex and the first ten principal components.

Study-specific acknowledgements

23andMe: We would like to thank the research participants and employees of 23andMe for making this work possible.

DeCODE: The financial support from the European Commission to the NeuroPain project (FP7#HEALTH-2013-602891-2) and painFACT project (H2020-2020-848099), and the National Institutes of Health (R01DE022905) is acknowledged.

GeneRISK: We thank the study participants for their contribution to this research. The GeneRISK study was funded by Business Finland through the Personalized Diagnostics and Care program coordinated by SalWe Ltd (Grant No 3986/31/2013).

HUNT: The Trøndelag Health Study (HUNT) is a collaboration between the HUNT Research Center (Faculty of Medicine and Health Sciences, NTNU, Norwegian University of Science and Technology), Trøndelag County Council, Central Norway Regional Health Authority, and the Norwegian Institute of Public Health. The genotyping was financed by the National Institute of health (NIH), University of Michigan, The Norwegian Research council, and Central Norway Regional Health Authority and the Faculty of Medicine and Health Sciences, Norwegian University of Science and Technology (NTNU). The genotype quality control and imputation has been conducted by the K.G. Jebsen center for genetic epidemiology, Department of public health and nursing, Faculty of medicine and health sciences, Norwegian University of Science and Technology (NTNU).

IHGC2016: Study-specific acknowledgements for the 21 cohorts included in the IHGC2016 can be found from[15].

NEO: We express our gratitude to all individuals who participate in the Netherlands Epidemiology in Obesity study. We are grateful to all participating general practitioners for inviting eligible participants. We also thank P. van Beelen and all research nurses for collecting the data and P. Noordijk and her team for sample handling and storage and I. de Jonge, MSc for data management of the NEO study. The NEO study is supported by the participating Departments, the Division and

the Board of Directors of the Leiden University Medical Centre, and by the Leiden University, Research Profile Area 'Vascular and Regenerative Medicine'.

UKBB: We thank the study participants for their contribution to this research. This research has been conducted using the UK Biobank Resource under Application Number 22627.

Ethics statement

All participating studies were approved by local research ethics committees and written informed consent was obtained from all study participants. For all the participating studies, an approval was received to use the data in the present work.

Derivation of effective sample size N_{eff}

We define $N_{eff} = 2N\phi(1 - \phi)I_i$, where N = total sample size (cases + controls), $\phi = \frac{N_{cases}}{N}$, and I_i is an imputation info for a variant i. The standard error (se) from logistic regression for a variant i is approximately: $se_i = \frac{1}{\sqrt{2N\phi(1-\phi)I_i f_i(1-f_i)}}$, where f_i is a frequency of variant i. Then

$$se_i^2 \approx \frac{1}{2N\phi(1-\phi)I_i f_i(1-f_i)} = \frac{1}{N_{eff(i)} f_i(1-f_i)}$$

and

$$N_{eff(i)} \approx \frac{1}{f_i(1-f_i)\, se_i^2}.$$

We note that our definition of N_{eff} matches exactly with the definition of effective sample size given by[32] for perfectly observed variants with info value of $I_i = 1$: They write the formula as $N_{eff} = 2/\left({}^{1}/_{N\phi} + {}^{1}/_{N(1-\phi)}\right)$ without an extension to imputed genotypes where $I_i < 1$.

Enrichment of migraine associated variants for the GTEx v8 tissues

GTEx v8 data[33] downloaded from (gtexportal.org) contain 49 tissues with sample size over 70 and 23,268 genes that have at least one significant *cis*-eQTL at FDR 5%. The number of genes that have at least one significant *cis*-eQTL found per tissue in GTEx v8 and the tissue sample size are highly correlated, and GWAS associated variants are enriched for *cis*-eQTLs compared to all variants tested (1.46-fold)[33].

To study whether migraine associated variants are enriched in any of the 49 tissues from GTEx v8, we first fitted a linear model where the number of migraine lead variants that overlap with significant *cis*-eQTLs for a specific tissue were used as an outcome, and the number of genes with at least one significant *cis*-eQTL for a specific tissue as a predictor (Supplementary Figure 8) using R software. The adjusted R^2 of the model was 0.90, and the predictor was statistically significant (P-value $< 2\times10^{-16}$). Next, for each tissue type we did a separate regression model by leaving the tissue of interest out from the model, and then predicted the outcome by using the model fitted on the other tissues together with its 95% prediction intervals (Supplementary Figure 4). A Z-score was formed by dividing the difference between the observed and the predicted value by an estimate of its standard error that was estimated as one quarter of the length of the 95% prediction interval. P-values for the squared Z-scores were estimated from the χ^2-distribution with one degree of freedom, and we applied Bonferroni correction ($\alpha = 0.05/49$) to identify tissues that were enriched for migraine associated variants.

Consortia

International Headache Genetics Consortium

Verneri Anttila[1,2,3], Ville Artto[4], Andrea C Belin[5], Anna Bjornsdottir[6], Gyda Bjornsdottir[7], Dorret I Boomsma[8], Sigrid Børte[9,10,11], Mona A Chalmer[12], Daniel I Chasman[13,14], Bru Cormand[15], Ester Cuenca-Leon[16], George Davey-Smith[17], Irene de Boer[18], Martin Dichgans[19,20], Tonu Esko[21], Tobias Freilinger[22,23], Padhraig Gormley[24], Lyn R Griffiths[25], Eija Hämäläinen[26], Thomas F Hansen[12,27], Aster VE Harder[18,28], Heidi Hautakangas[26], Marjo Hiekkala[29], Maria G Hrafnsdottir[30], M. Arfan Ikram[31], Marjo-Riitta Järvelin[32,33,34,35], Risto Kajanne[26], Mikko Kallela[4], Jaakko Kaprio[26], Mari Kaunisto[29], Lisette JA Kogelman[12], Espen S Kristoffersen[36,37,38], Christian Kubisch[39], Mitja Kurki[40], Tobias Kurth[41], Lenore Launer[42], Terho Lehtimäki[43], Davor Lessel[39], Lannie Ligthart[8], Sigurdur H Magnusson[7], Rainer Malik[19], Bertram Müller-Myhsok[44], Carrie Northover[45], Dale R Nyholt[46], Jes Olesen[12], Aarno Palotie[26,47], Priit Palta[26], Linda M Pedersen[48], Nancy Pedersen[49], Matti Pirinen[26,50,51], Danielle Posthuma[52], Patricia Pozo-Rosich[53], Alice Pressman[54], Olli Raitakari[55,56,57], Caroline Ran[5], Gudrun R Sigurdardottir[6], Hreinn Stefansson[7], Kari Stefansson[7], Olafur A Sveinsson[30], Gisela M Terwindt[18], Thorgeir E Thorgeirsson[7], Arn MJM van den Maagdenberg[18,28], Cornelia van Duijn[58], Maija Wessman[26,29], Bendik S Winsvold[9,48,59], John-Anker Zwart[9,10,48]

[1]Analytical and Translational Genetics Unit, Department of Medicine, Massachusetts General Hospital and Harvard Medical School, Boston, Massachusetts, USA; [2]Program in Medical and Population Genetics, Broad Institute of MIT and Harvard, Cambridge, Massachusetts, USA; [3]Stanley Center for Psychiatric Research, Broad Institute of MIT and Harvard, Cambridge, Massachusetts, USA; [4]Department of Neurology, Helsinki University Central Hospital, Helsinki, Finland; [5]Department of Neuroscience, Karolinska Institutet, Stockholm, Sweden; [6]Neurology private practice, Laeknasetrid, Reykjavik, Iceland; [7]deCODE genetics/Amgen Inc., Reykjavik, Iceland; [8]Netherlands Twin Register, Department of Biological Psychology, Vrije Universiteit, Amsterdam, the Netherlands; [9]K.G. Jebsen Center for Genetic Epidemiology, Department of Public Health and Nursing, Faculty of Medicine and Health Sciences, Norwegian University of Science and Technology, Trondheim, Norway; [10]Institute of Clinical Medicine, Faculty of Medicine, University of Oslo, Oslo, Norway; [11]Research and Communication Unit for Musculoskeletal Health, Department of Research, Innovation and Education, Division of Clinical Neuroscience, Oslo University Hospital, Oslo, Norway; [12]Danish Headache Center, Department of Neurology, Copenhagen University Hospital, Copenhagen, Denmark; [13]Department of Medicine, Division of Preventive Medicine, Brigham and Women's Hospital, Boston, Massachusetts, USA; [14]Harvard Medical School, Boston, Massachusetts, USA; [15]Department of Genetics, Spain Centre for Biomedical Network Research on Rare Diseases, University of Barcelona, Barcelona, Spain; [16]Pediatric Neurology Research Group, Vall d'Hebron Research Institute, Barcelona, Spain; [17]University of Bristol/Medical Research Council Integrative Epidemiology Unit, University of Bristol, Bristol, UK; [18]Department of Neurology, Leiden University Medical Centre, Leiden, the Netherlands; [19]Institute for Stroke and Dementia Research, University Hospital, LMU Munich, Munich, Germany; [20]Munich Cluster for Systems Neurology, Munich, Germany; [21]Estonian Biobank Registry,

the Estonian Genome Center, University of Tartu, Tartu, Estonia; [22]Department of Neurology, Klinikum Passau, Passau, Germany; [23]Department of Neurology and Epileptology, Hertie Institute for Clinical Brain Research, University of Tuebingen, Tuebingen, Germany; [24]GSK Inc., Cambridge, Massachusetts, USA; [25]Centre for Genomics and Personalised Health, Queensland University of Technology, Brisbane, Queensland, Australia; [26]Institute for Molecular Medicine Finland, Helsinki Institute of Life Science, University of Helsinki, Helsinki, Finland; [27]Novo Nordic Foundation Center for Protein Research, Copenhagen University, Copenhagen, Denmark; [28]Department of Human Genetics, Leiden University Medical Centre, Leiden, the Netherlands; [29]Folkhälsan Research Center, Helsinki, Finland; [30]Landspitali University Hospital, Reykjavik, Iceland; [31]Department of Epidemiology, Erasmus University Medical Center, Rotterdam, the Netherlands; [32]Department of Epidemiology and Biostatistics, MRC-PHE Centre for Environment and Health, School of Public Health, Imperial College London, London, UK; [33]Center for Life Course Health Research, Faculty of Medicine, University of Oulu, Oulu, Finland; [34]Unit of Primary Health Care, Oulu University Hospital, OYS, Oulu, Finland; [35]Department of Life Sciences, College of Health and Life Sciences, Brunel University London, London, UK; [36]Research and Communication Unit for Musculoskeletal Health, Department of Research, Innovation and Education, Division of Clinical Neuroscience, Akershus University Hospital and University of Oslo, Oslo, Norway; [37]Department of General Practice, Institute of Health and Society, University of Oslo, Oslo, Norway; [38]Department of Neurology, Akershus University Hospital, Lørenskog, Norway; [39]Institute of Human Genetics, University Medical Center Hamburg-Eppendorf, Hamburg, Germany; [40]Psychiatric and Neurodevelopmental Genetics Unit, Department of Medicine, Massachusetts General Hospital, Boston, Massachusetts, USA; [41]Institute of Public Health, Charité – Universitätsmedizin, Berlin; [42]Laboratory of Epidemiology and Population Sciences, Intramural Research Program, National Institute on Aging, Bethesda, Maryland, USA; [43]Department of Clinical Chemistry, Fimlab Laboratories, and Finnish Cardiovascular Research Center - Tampere, Faculty of Medicine and Health Technology, Tampere University, Tampere, Finland; [44]Max Planck Institute of Psychiatry, Munich, Germany; [45]23&Me Inc., Mountain View, California, USA; [46]School of Biomedical Sciences, Faculty of Health, Centre for Genomics and Personalised Health, Centre for Data Science, Queensland University of Technology, Brisbane, Queensland, Australia; [47]University of Helsinki, Helsinki, Finland; [48]Department of Research, Innovation and Education, Division of Clinical Neuroscience, Oslo University Hospital, Oslo, Norway; [49]Department of Medical Epidemiology and Biostatistics, Karolinska Institutet, Stockholm, Sweden; [50]Department of Mathematics and Statistics, University of Helsinki, Helsinki, Finland; [51]Department of Public Health, University of Helsinki, Helsinki, Finland; [52]Department of Complex Trait Genetics, Center for Neurogenomics and Cognitive Research, Neuroscience Campus Amsterdam, VU University, Amsterdam, The Netherlands; [53]Headache Unit, Neurology Department, Vall d'Hebron University Hospital, Barcelona, Spain; [54]Sutter Health, Sacramento, California, USA; [55]Centre for Population Health Research, University of Turku, Turku University Hospital, Turku, Finland; [56]Research Centre of Applied and Preventive Cardiovascular Medicine, University of Turku, Turku, Finland; [57]Department of Clinical Physiology and Nuclear Medicine, Turku University Hospital, Turku, Finland; [58]Department of Epidemiology, Erasmus University Medical Centre, Rotterdam, the Netherlands; [59]Department of Neurology, Oslo University Hospital, Oslo, Norway

HUNT All-in Headache

Amy E Martinsen[1,2,3], Anne Heidi Skogholt[3], Ben M Brumpton[3], Bendik S Winsvold[4,5,6], Cristen J Willer[7], Erling Tronvik[8,9], Espen Saxhaug Kristoffersen[1,10,11], John-Anker Zwart[1,2,3], Jonas B Nielsen[3,7,12], Knut Hagen[8], Kristian Hveem[3,13,14], Kristian Bernhard Nilsen[8,15], Lars G Fritsche[16], Lars Jacob Stovner[8,17], Laurent F Thomas[3,18,19,20], Linda M Pedersen[1], Maiken E Gabrielsen[3], Marianne B Johnsen[3,21], Marie U Lie[2,21], Oddgeir L Holmen[13], Sigrid Børte[2,3,21], Wei Zhou[22,23]

[1]Department of Research and Innovation, Division of Clinical Neuroscience, Oslo University Hospital, Oslo, Norway, [2]Institute of Clinical Medicine, Faculty of Medicine, University of Oslo, Oslo, Norway, [3]K. G. Jebsen Center for Genetic Epidemiology, Department of Public Health and Nursing, Faculty of Medicine and Health Sciences, Norwegian University of Science and Technology (NTNU), Trondheim, Norway, [4]Department of Research and Innovation, Division of Clinical Neuroscience, Oslo University Hospital, Oslo, Norway, [5]K. G. Jebsen Center for Genetic Epidemiology, Department of Public Health and Nursing, Faculty of Medicine and Health Sciences, Norwegian University of Science and Technology (NTNU), Trondheim, Norway, [6]Department of Neurology, Oslo University Hospital, Oslo, Norway, [7]Department of Internal Medicine, Division of Cardiovascular Medicine, University of Michigan, Ann Arbor, MI, 48109, USA, [8]Department of Neuromedicine and Movement Science, Faculty of Medicine and Health Sciences, Norwegian University of Science and Technology (NTNU), Trondheim, Norway, [9]Department of Neurology and Clinical Neurophysiology, St. Olavs Hospital, Trondheim University Hospital, Trondheim, Norway, [10]Department of General Practice, University of Oslo, Oslo, Norway, [11]Department of Neurology, Akershus University Hospital, Lørenskog, Norway, [12]Department of Epidemiology Research, Statens Serum Institut, Copenhagen, Denmark, [13]HUNT Research Center, Department of Public Health and Nursing, Faculty of Medicine and Health Sciences, Norwegian University of Science and Technology (NTNU), Trondheim, Norway, [14]Department of Research, Innovation and Education, St. Olavs Hospital, Trondheim University Hospital, Trondheim, Norway, [15]Department of Neurology, Oslo University Hospital, Oslo, Norway, [16]Center for Statistical Genetics, Department of Biostatistics, University of Michigan, Ann Arbor, MI, 48109, USA, [17]Norwegian Advisory Unit on Headaches, St. Olavs Hospital, Trondheim University Hospital, Trondheim, Norway, [18]Department of Clinical and Molecular Medicine, Norwegian University of Science and Technology (NTNU), Trondheim, Norway, [19]BioCore - Bioinformatics Core Facility, Norwegian University of Science and Technology (NTNU), Trondheim, Norway, [20]Clinic of Laboratory Medicine, St. Olavs Hospital, Trondheim University Hospital, Trondheim, Norway, [21]Research and Communication Unit for Musculoskeletal Health (FORMI), Department of Research and Innovation, Division of Clinical Neuroscience, Oslo University Hospital, Oslo, Norway, [22]Department of Computational Medicine and Bioinformatics, University of Michigan, Ann Arbor, MI, 48109, USA, [23]Analytic and Translational Genetics Unit, Massachusetts General Hospital, Boston, MA, USA

Danish Blood Donor Study Genomic Cohort

Steffen Andersen[1], Karina Banasik[2], Søren Brunak[2], Kristoffer Burgdorf[3], Christian Erikstrup[4], Thomas Folkmann Hansen[5], Henrik Hjalgrim[6], Gregor Jemec[7], Poul Jennum[8], Per Ingemar Johansson[3], Kasper Rene Nielsen[9], Mette Nyegaard[10], Topholm Bruun Mie[11], Ole Birger Pedersen[12], Susan Mikkelsen[4], Khoa Manh Dinh[4], Erik Sørensen[3], Henrik Ullum[3], Sisse Ostrowski[3], Thomas Werge[13], Pär Ingemar Johansson[9], Daniel Gudbjartsson[14], Kari Stefansson[14], Hreinn Stefánsson[14], Unnur Þorsteinsdóttir[14], Margit Anita Hørup Larsen[3], Maria Didriksen[3], Susanne Sækmose[12]

[1]Department of Finance Copenhagen Business School, Copenhagen, Denmark. [2]Novo Nordisk Foundation Center for Protein Research Faculty of Health and Medical Sciences, University of Copenhagen, Copenhagen, Denmark. [3]Department of Clinical Immunology, Copenhagen University Hospital, Copenhagen, Denmark. [4]Department of Clinical Immunology, Aarhus University Hospital, Aarhus, Denmark. [5]Danish Headache Center department of Neurology, Rigshospitalet Glostrup, Denmark. [6]Department of Epidemiology, Research Statens Serum Institut, Copenhagen, Denmark. [7]Department of Clinical Medicine, Sealand University hospital, Roskilde, Denmark. [8]Department of clinical neurophysiology at University of Copenhagen, Copenhagen, Denmark. [9]Department of Clinical Immunology Copenhagen, University Hospital, Copenhagen, Denmark. [10]Department of Biomedicine, Aarhus University, Aarhus, Denmark. [11]Department of Clinical Immunology, Odense University Hospital, Odense, Denmark. [12]Department of Clinical Immunology, Naestved Hospital, Naestved, Denmark. [13]Institute of Biological Psychiatry, Mental Health Centre Sct. Hans, Copenhagen University Hospital, Roskilde, Denmark. [14]deCODE genetics, Reykjavik, Iceland

Supplementary References

1. Pickrell, J.K. *et al*. Detection and interpretation of shared genetic influences on 42 human traits. *Nature Genetics* **48**, 709-717 (2016).
2. Browning, S.R. & Browning, B.L. Rapid and Accurate Haplotype Phasing and Missing-Data Inference for Whole-Genome Association Studies By Use of Localized Haplotype Clustering. *American Journal of Human Genetics* **81**, 1084-1097 (2007).
3. Krokstad, S. *et al*. Cohort Profile: The HUNT Study, Norway. *International Journal of Epidemiology* **42**, 968-977 (2012).
4. Headache Classification Subcommittee of the International Headache, S. The International Classification of Headache Disorders: 2nd edition. *Cephalalgia* **24 Suppl 1**, 9-160 (2004).
5. Hagen, K., Zwart, J.A., Vatten, L., Stovner, L.J. & Bovim, G. Head-HUNT: Validity and Reliability of a Headache Questionnaire in a Large Population-Based Study in Norway. *Cephalalgia* **20**, 244-251 (2000).
6. Hagen, K. *et al*. The validity of questionnaire-based diagnoses: the third Nord-Trondelag Health Study 2006-2008. *The Journal of Headache and Pain* **11**, 67-73 (2010).
7. Jun, G. *et al*. Detecting and estimating contamination of human DNA samples in sequencing and array-based genotype data. *American Journal of Human Genetics* **91**, 839-848 (2012).
8. Guo, Y. *et al*. Illumina human exome genotyping array clustering and quality control. *Nature Protocols* **9**, 2643-2662 (2014).
9. Consortium, E.P. An integrated encyclopedia of DNA elements in the human genome. *Nature* **489**, 57-74 (2012).
10. Chang, C.C. *et al*. Second-generation PLINK: rising to the challenge of larger and richer datasets. *GigaScience* **4**, s13742-8 (2015).
11. Wang, C. *et al*. Ancestry estimation and control of population stratification for sequence-based association studies. *Nature Genetics* **46**, 409-415 (2014).
12. Li Jun, Z. *et al*. Worldwide Human Relationships Inferred from Genome-Wide Patterns of Variation. *Science* **319**, 1100-1104 (2008).
13. Das, S. *et al*. Next-generation genotype imputation service and methods. *Nature Genetics* **48**, 1284-1287 (2016).
14. McCarthy, S. *et al*. A reference panel of 64,976 haplotypes for genotype imputation. *Nature Genetics* **48**, 1279-1283 (2016).
15. Gormley, P. *et al*. Meta-analysis of 375,000 individuals identifies 38 susceptibility loci for migraine. *Nature Genetics* **48**, 856-866 (2016).
16. Zhou, W. *et al*. Efficiently controlling for case-control imbalance and sample relatedness in large-scale genetic association studies. *Nature Genetics* **50**, 1335-1341 (2018).
17. Bycroft, C. *et al*. The UK Biobank resource with deep phenotyping and genomic data. *Nature* **562**, 203-209 (2018).
18. Manichaikul, A. *et al*. Robust relationship inference in genome-wide association studies. *Bioinformatics* **26**, 2867-2873 (2010).
19. Hansen, T.F. *et al*. DBDS Genomic Cohort, a prospective and comprehensive resource for integrative and temporal analysis of genetic, environmental and lifestyle factors affecting health of blood donors. *BMJ Open* **9**, e028401 (2019).
20. Kirchmann, M. *et al*. Validation of the deCODE Migraine Questionnaire (DMQ3) for use in genetic studies. *European Journal of Neurology* **13**, 1239-1244 (2006).
21. Gudbjartsson, D.F. *et al*. Large-scale whole-genome sequencing of the Icelandic population. *Nature Genetics* **47**, 435-444 (2015).
22. Jónsson, H. *et al*. Whole genome characterization of sequence diversity of 15,220 Icelanders. *Scientific Data* **4**, 170115 (2017).

23. Eggertsson, H.P. *et al*. GraphTyper2 enables population-scale genotyping of structural variation using pangenome graphs. *Nature Communications* **10**, 5402 (2019).
24. Kong, A. *et al*. Detection of sharing by descent, long-range phasing and haplotype imputation. *Nature Genetics* **40**, 1068-1075 (2008).
25. Gudbjartsson, D.F. *et al*. Sequence variants from whole genome sequencing a large group of Icelanders. *Scientific Data* **2**, 150011 (2015).
26. Launer, L.J., Terwindt, G.M. & Ferrari, M.D. The prevalence and characteristics of migraine in a population-based cohort: the GEM study. *Neurology* **53**, 537-542 (1999).
27. Headache Classification Committee of the International Headache Society (IHS). Headache Classification Committee of the International Headache Society (IHS) The International Classification of Headache Disorders, 3rd edition. *Cephalalgia* **38**, 1-211 (2018).
28. van Oosterhout, W.P.J. *et al*. Validation of the web-based LUMINA questionnaire for recruiting large cohorts of migraineurs. *Cephalalgia* **31**, 1359-1367 (2011).
29. de Mutsert, R. *et al*. The Netherlands Epidemiology of Obesity (NEO) study: study design and data collection. *European Journal of Epidemiology* **28**, 513-523 (2013).
30. Loh, P.-R., Palamara, P.F. & Price, A.L. Fast and accurate long-range phasing in a UK Biobank cohort. *Nature Genetics* **48**, 811-816 (2016).
31. Marchini, J., Howie, B., Myers, S., McVean, G. & Donnelly, P. A new multipoint method for genome-wide association studies by imputation of genotypes. *Nature Genetics* **39**, 906-913 (2007).
32. Winkler, T.W. *et al*. Quality control and conduct of genome-wide association meta-analyses. *Nature Protocols* **9**, 1192-1212 (2014).
33. The GTEx Consortium. The GTEx Consortium atlas of genetic regulatory effects across human tissues. *Science* **369**, 1318-1330 (2020).

Supplementary Figures

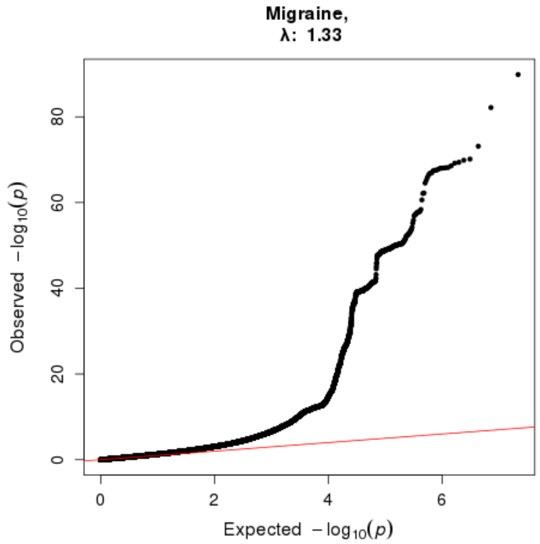

Supplementary Figure 1. **QQ-plot of the migraine inverse-variance weighted fixed-effect meta-analysis test statistics with 102,084 cases and 771,257 controls.** A genomic inflation factor (λ_{GC}) is 1.33. X-axis shows the expected -log10 *P*-value for each quantile, and Y-axis shows the observed -log10 *P*-value for the corresponding quantile.

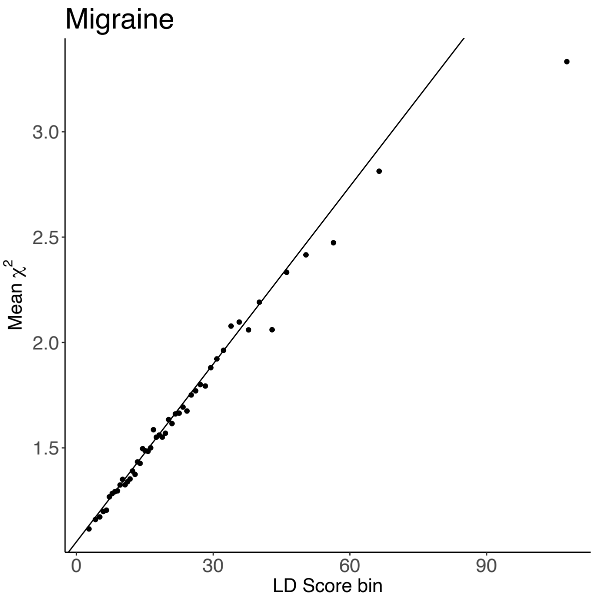

Supplementary Figure 2. **LD Score plot for migraine**. Plot shows linear trend between LD Score bins and mean migraine GWAS summary statistics of each bin. Heritability is estimated by

regressing GWAS summary statistics against LD Scores. A deviation of the intercept term from 1 would indicate confounding inflation of the association statistics, such as population stratification or model misspecification. The univariate LDSC intercept was 1.05 (s.e. 0.01) and the LDSC ratio between the intercept and mean χ^2-statistics was 0.078, suggesting that 92.2% of the observed inflation in χ^2-statistics is due to polygenicity of migraine.

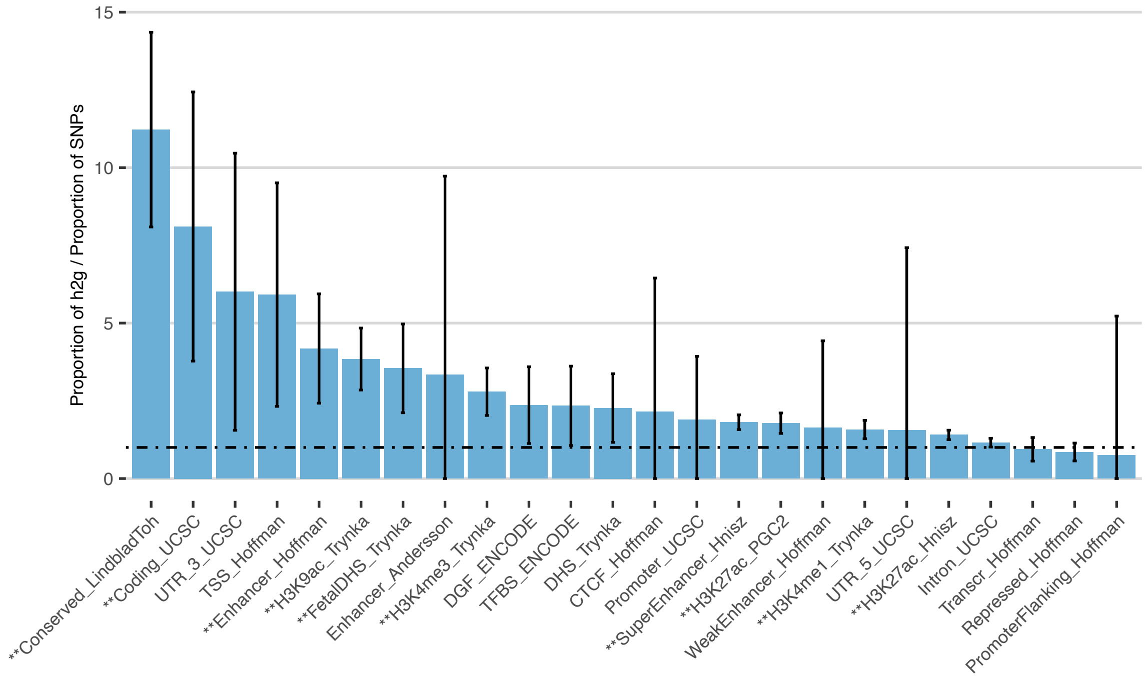

Supplementary Figure 3. Enrichment estimates for the 24 main functional annotations for migraine (N=873,341) with 95%-confidence intervals from the stratified LD Score regression. The black dash-dotted line at 1 denotes no enrichment, and values above 1 indicate enriched heritability for a given category and values below 1 depleted heritability for the given category. Two asterisks indicate significance at $P < 0.05/24$ (Bonferroni correction for the 24 hypotheses tested). P-values are derived a two-sided test for enrichment or depletion. Confidence intervals are truncated from below at 0.

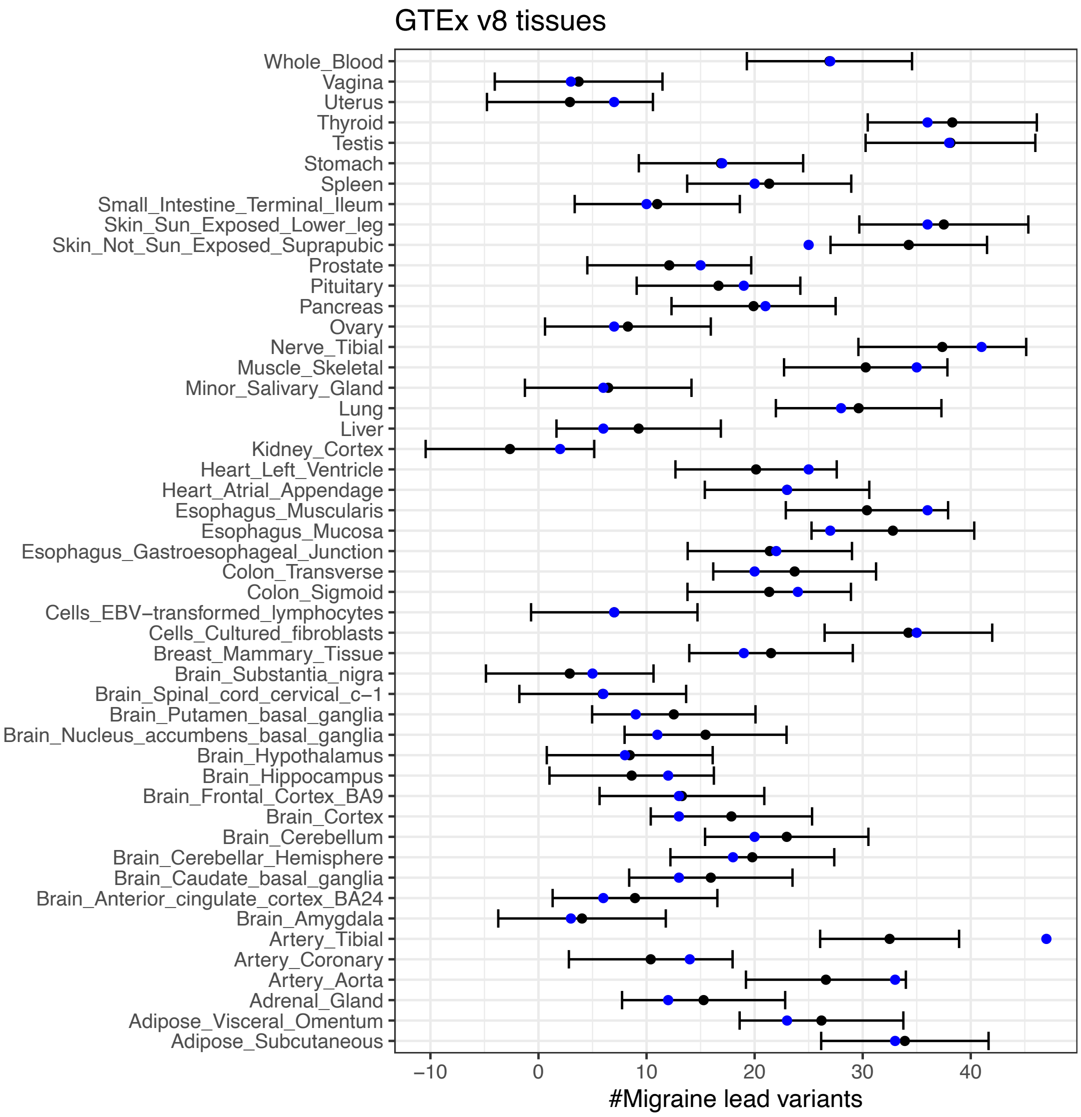

Supplementary Figure 4. **Results from a prediction model of enriched tissues in GTEx v8 data for migraine lead variants.** Y-axis shows each of the 49 tissues, and X-axis shows the number of migraine lead variants that are also significant *cis*-eQTLs for each tissue. Black dot is the predicted value from a linear regression trained on the other 48 tissues, where the number of migraine lead variants that are significant *cis*-eQTLs for each tissue was used as the outcome, and the overall number of genes with at least one significant *cis*-eQTL reported by GTEx for each tissue was the predictor. Black lines are the 95% prediction intervals. Blue dots are the true observed values. When the observed value is far from the prediction interval, the corresponding tissue has an exceptional enrichment or depletion of eQTLs among the migraine lead variants compared to the other tissues.

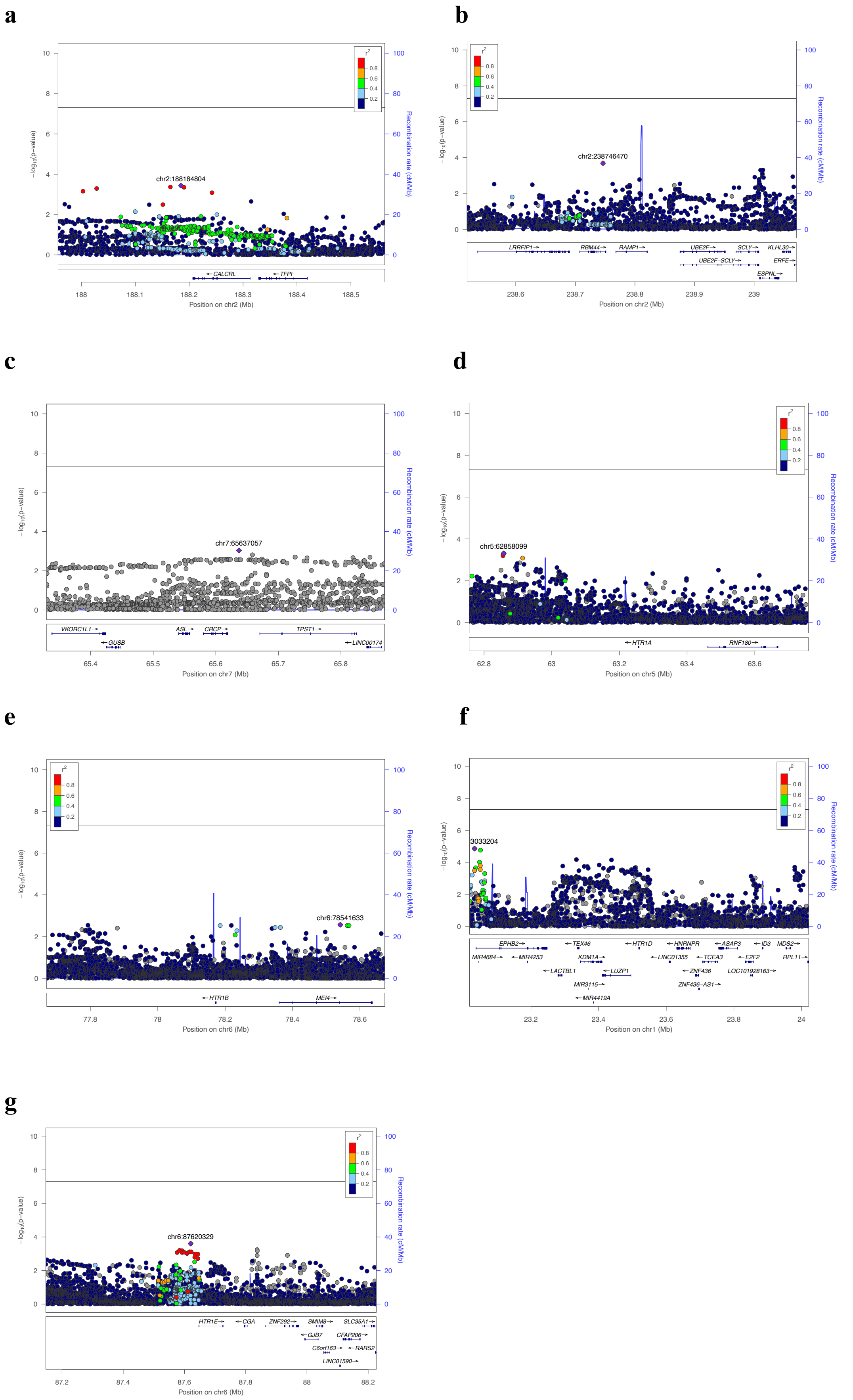

a
chr2:188184804
CALCRL
TFPI
Position on chr2 (Mb)
b
chr2:238746470
LRRFIP1
RBM44
RAMP1
UBE2F
SCLY
KLHL30
UBE2F−SCLY
ERFE
ESPNL
Position on chr2 (Mb)
c
chr7:65637057
VKORC1L1
GUSB
ASL
CRCP
TPST1
LINC00174
Position on chr7 (Mb)
d
chr5:62858099
HTR1A
RNF180
Position on chr5 (Mb)
e
chr6:78541633
HTR1B
MEI4
Position on chr6 (Mb)
f
3033204
EPHB2
MIR4684
MIR4253
TEX46
KDM1A
LACTBL1
MIR3115
MIR4419A
LUZP1
HTR1D
HNRNPR
LINC01355
ZNF436
ZNF436−AS1
TCEA3
ASAP3
E2F2
LOC101928163
ID3
MDS2
RPL11
Position on chr1 (Mb)
g
chr6:87620329
HTR1E
CGA
ZNF292
GJB7
SMIM8
C6orf163
LINC01590
SLC35A1
CFAP206
RARS2
Position on chr6 (Mb)
−log10(p−value)
Recombination rate (cM/Mb)
r2

Supplementary Figure 5. **Regional plots for genes related to receptors that are targets of migraine specific drugs.** Locuszoom-plots of three genes, a) *CALCRL*, b) *RAMP1* and c) *CRCP*, that encode central proteins of CGRP receptor complex, and four genes d) *HTR1A*, e) *HTR1B*, f) *HTR1D* and g) *HTR1E* that encode serotonin 5-HT_1 receptors. None of the seven regions show a clear association with migraine even though effective therapies targeting CGRP receptor and serotonin 5-$HT_{1B/1D}$ receptors exist. X-axis shows the chromosomal location, and Y-axis shows the strength of the association as uncorrected two-sided negative log10 *P*-value from the inverse-variance weighted fixed-effects meta-analysis (N = 873,341; 102,084 cases and 771,257 controls). The squared correlation to the lead variant is shown by colors based on the UK Biobank data. Black horizontal line corresponds to $P = 5 \times 10^{-8}$. Blue line shows the recombination rate.

a

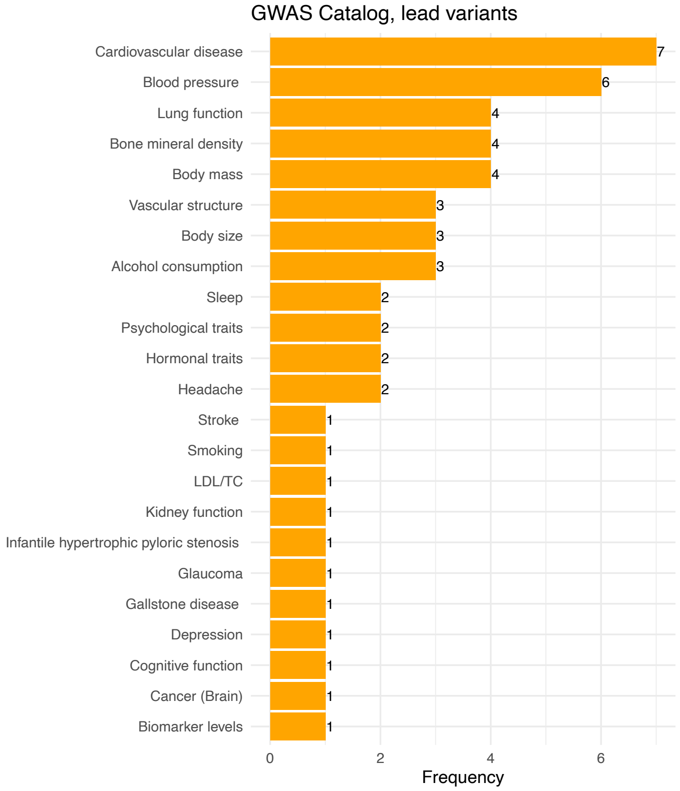
GWAS Catalog, lead variants
Cardiovascular disease 7
Blood pressure 6
Lung function 4
Bone mineral density 4
Body mass 4
Vascular structure 3
Body size 3
Alcohol consumption 3
Sleep 2
Psychological traits 2
Hormonal traits 2
Headache 2
Stroke 1
Smoking 1
LDL/TC 1
Kidney function 1
Infantile hypertrophic pyloric stenosis 1
Glaucoma 1
Gallstone disease 1
Depression 1
Cognitive function 1
Cancer (Brain) 1
Biomarker levels 1
0
2
4
6
Frequency

b

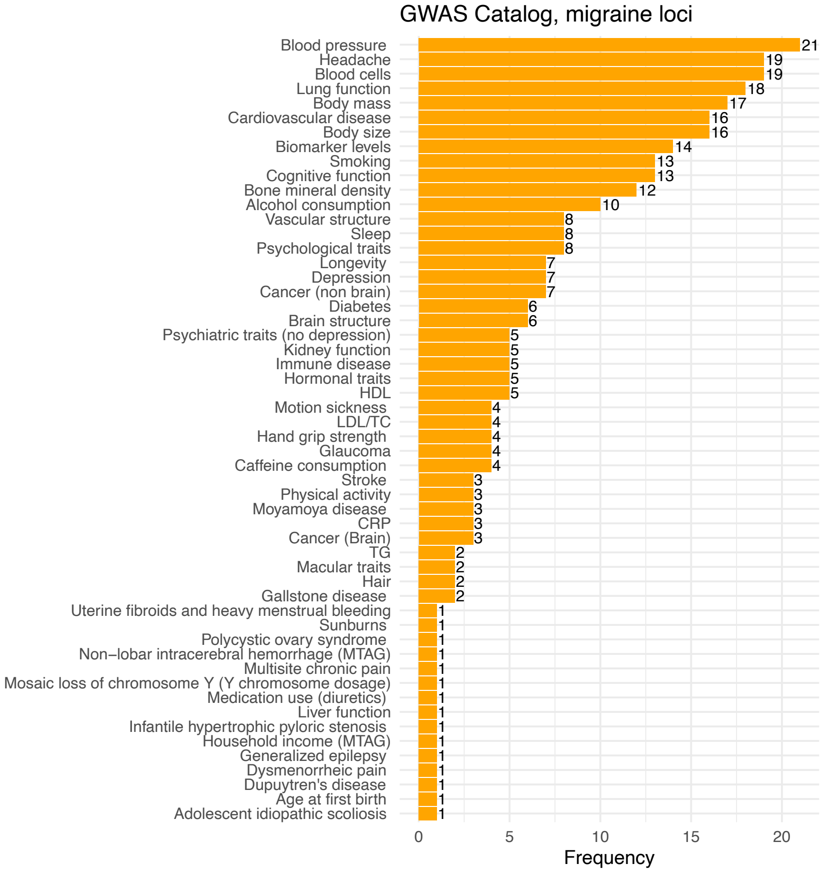
GWAS Catalog, migraine loci
Blood pressure 21
Headache 19
Blood cells 19
Lung function 18
Body mass 17
Cardiovascular disease 16
Body size 16
Biomarker levels 14
Smoking 13
Cognitive function 13
Bone mineral density 12
Alcohol consumption 10
Vascular structure 8
Sleep 8
Psychological traits 8
Longevity 7
Depression 7
Cancer (non brain) 7
Diabetes 6
Brain structure 6
Psychiatric traits (no depression) 5
Kidney function 5
Immune disease 5
Hormonal traits 5
HDL 5
Motion sickness 4
LDL/TC 4
Hand grip strength 4
Glaucoma 4
Caffeine consumption 4
Stroke 3
Physical activity 3
Moyamoya disease 3
CRP 3
Cancer (Brain) 3
TG 2
Macular traits 2
Hair 2
Gallstone disease 2
Uterine fibroids and heavy menstrual bleeding 1
Sunburns 1
Polycystic ovary syndrome 1
Non−lobar intracerebral hemorrhage (MTAG) 1
Multisite chronic pain 1
Mosaic loss of chromosome Y (Y chromosome dosage) 1
Medication use (diuretics) 1
Liver function 1
Infantile hypertrophic pyloric stenosis 1
Household income (MTAG) 1
Generalized epilepsy 1
Dysmenorrheic pain 1
Dupuytren's disease 1
Age at first birth 1
Adolescent idiopathic scoliosis 1
0
5
10
15
20
Frequency

c

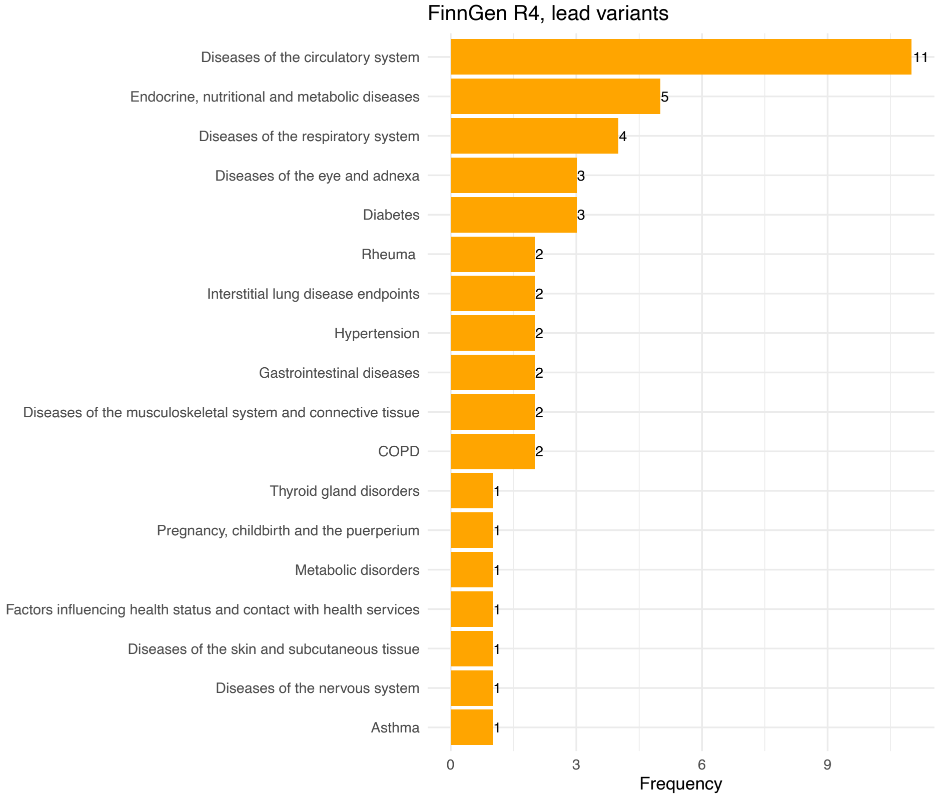
FinnGen R4, lead variants
Diseases of the circulatory system
Endocrine, nutritional and metabolic diseases
Diseases of the respiratory system
Diseases of the eye and adnexa
Diabetes
Rheuma
Interstitial lung disease endpoints
Hypertension
Gastrointestinal diseases
Diseases of the musculoskeletal system and connective tissue
COPD
Thyroid gland disorders
Pregnancy, childbirth and the puerperium
Metabolic disorders
Factors influencing health status and contact with health services
Diseases of the skin and subcutaneous tissue
Diseases of the nervous system
Asthma
11
5
4
3
3
2
2
2
2
2
2
1
1
1
1
1
1
1
0
3
6
9
Frequency

d

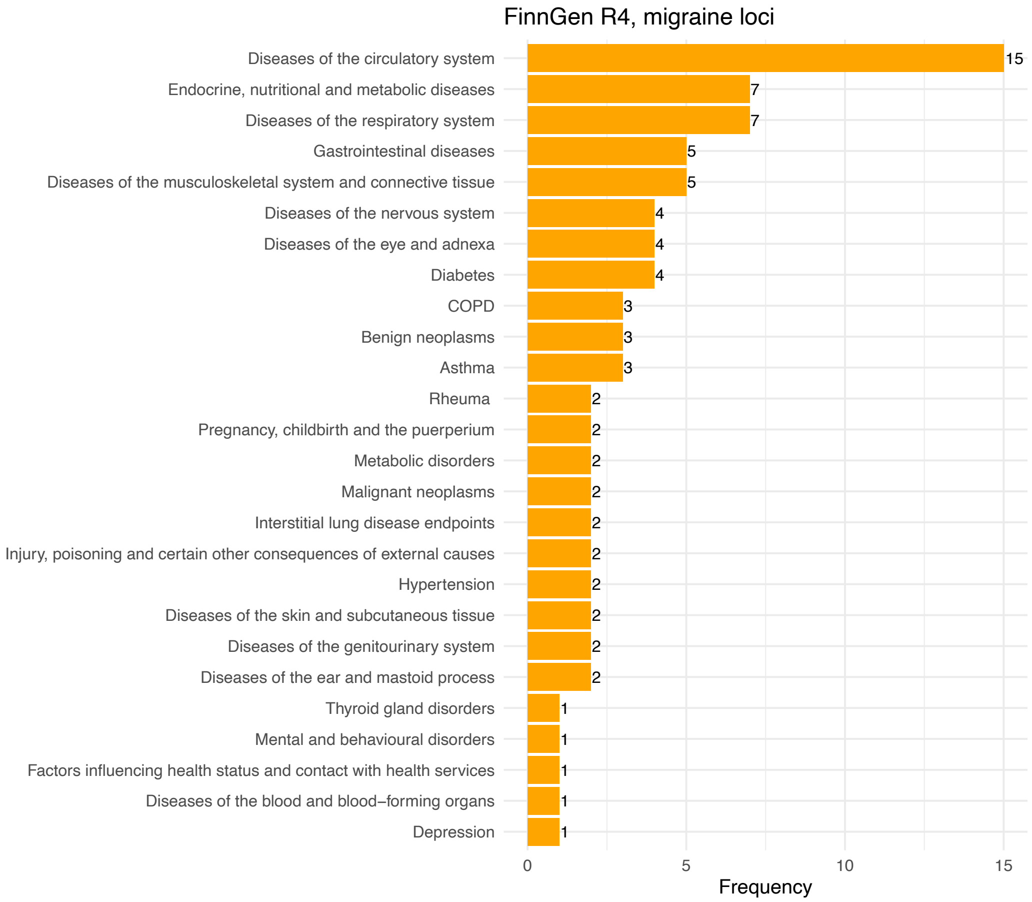
FinnGen R4, migraine loci
Diseases of the circulatory system
Endocrine, nutritional and metabolic diseases
Diseases of the respiratory system
Gastrointestinal diseases
Diseases of the musculoskeletal system and connective tissue
Diseases of the nervous system
Diseases of the eye and adnexa
Diabetes
COPD
Benign neoplasms
Asthma
Rheuma
Pregnancy, childbirth and the puerperium
Metabolic disorders
Malignant neoplasms
Interstitial lung disease endpoints
Injury, poisoning and certain other consequences of external causes
Hypertension
Diseases of the skin and subcutaneous tissue
Diseases of the genitourinary system
Diseases of the ear and mastoid process
Thyroid gland disorders
Mental and behavioural disorders
Factors influencing health status and contact with health services
Diseases of the blood and blood–forming organs
Depression
15
7
7
5
5
4
4
4
3
3
3
2
2
2
2
2
2
2
2
2
2
1
1
1
1
1
0
5
10
15
Frequency

Supplementary Figure 6. **PheWAS results for migraine associated variants.** Associations from the PheWAS of NHGRI GWAS Catalog and FinnGen R4 for 123 migraine risk loci grouped into broad phenotype categories (Methods). Associations are defined based on uncorrected two-sided *P*-value from GWAS at $P < 1 \times 10^{-5}$. X-axis shows the frequency of lead variants or loci for each category. a) Lead variants in GWAS Catalog; b) Lead variants and variants in high LD in GWAS Catalog; c) Lead variants in FinnGen R4; d) Lead variants and variants in high LD in FinnGen R4.

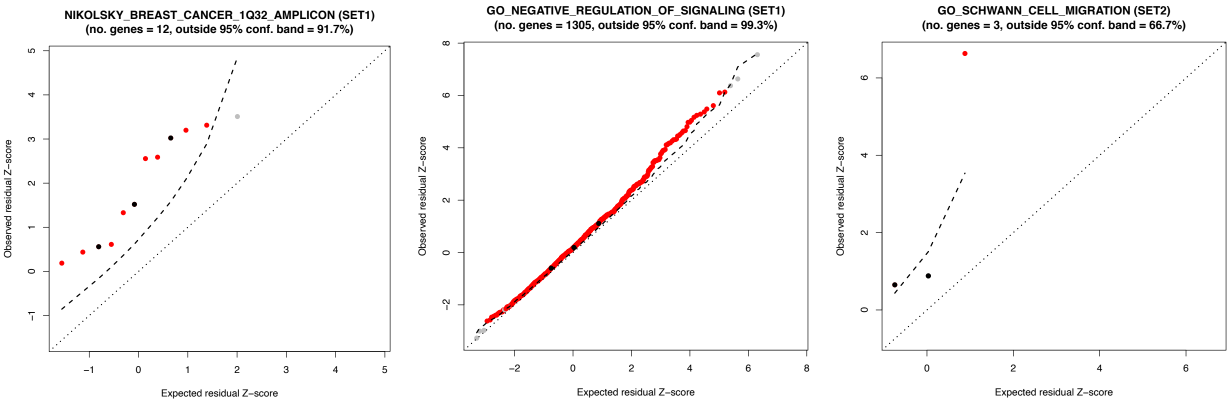

Supplementary Figure 7. **Gene-set specific QQ-plots from MAGMA analysis.**

Gene-set specific QQ-plots of the residual Z-scores from the MAGMA gene-set analysis for the gene sets that are significantly enriched for migraine associated genes after Bonferroni correction using either curated gene sets or GO gene sets. X-axis shows the excepted residual Z-scores from the null model based on the quantiles across all genes in the data and Y-axis shows the observed residual Z-scores. The black points indicate the 25th, 50th and 75th percentile. A one-sided 95% confidence band is the dashed black line. The red dots are quantiles that deviate more than would be expected by chance. One gene set (GO_SCHWANN_CELL_MIGRATION) was discarded based on the small size.

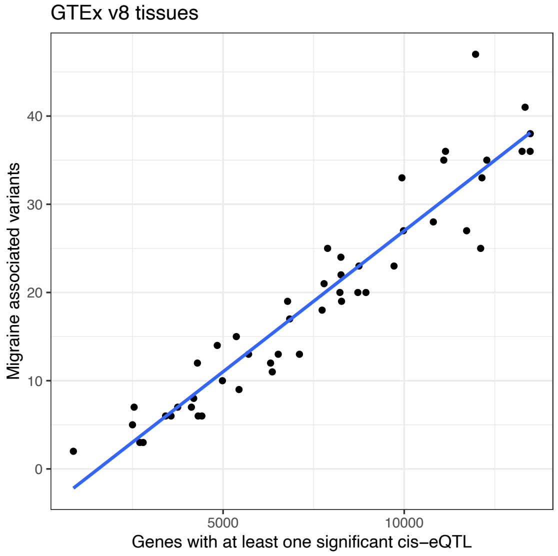

Supplementary Figure 8. **A Scatter plot of GTEx v8 tissues**. The X-axis shows the number of genes with at least one significant *cis*-eQTL for a given tissue and Y-axis shows the number of migraine lead variants that overlap with significant *cis*-eQTLs for that tissue. Blue line is the regression line from the fitted linear model.
