# Supplementary material for: Genome-wide analysis of 102,084 migraine cases identifies 123 risk loci and subtype-specific risk alleles
Source: Nat Genet. 2022 Feb 3;54(2):152–60. doi: 10.1038/s41588-021-00990-0 (PMC8837554; doi:10.1038/s41588-021-00990-0)
Supplement: Supplementary file 2 — Reporting Summary. [file 41588_2021_990_MOESM2_ESM.pdf]

nature portfolio

Corresponding author(s): Matti Pirinen

Last updated by author(s): Oct 8, 2021

Reporting Summary

Nature Portfolio wishes to improve the reproducibility of the work that we publish. This form provides structure for consistency and transparency in reporting. For further information on Nature Portfolio policies, see our Editorial Policies and the Editorial Policy Checklist.

Statistics

For all statistical analyses, confirm that the following items are present in the figure legend, table legend, main text, or Methods section.

n/a	Confirmed	
☐	☒	The exact sample size (*n*) for each experimental group/condition, given as a discrete number and unit of measurement
☒	☐	A statement on whether measurements were taken from distinct samples or whether the same sample was measured repeatedly
☐	☒	The statistical test(s) used AND whether they are one- or two-sided<br>*Only common tests should be described solely by name; describe more complex techniques in the Methods section.*
☐	☒	A description of all covariates tested
☐	☒	A description of any assumptions or corrections, such as tests of normality and adjustment for multiple comparisons
☐	☒	A full description of the statistical parameters including central tendency (e.g. means) or other basic estimates (e.g. regression coefficient) AND variation (e.g. standard deviation) or associated estimates of uncertainty (e.g. confidence intervals)
☐	☒	For null hypothesis testing, the test statistic (e.g. *F*, *t*, *r*) with confidence intervals, effect sizes, degrees of freedom and *P* value noted<br>*Give P values as exact values whenever suitable.*
☐	☒	For Bayesian analysis, information on the choice of priors and Markov chain Monte Carlo settings
☒	☐	For hierarchical and complex designs, identification of the appropriate level for tests and full reporting of outcomes
☐	☒	Estimates of effect sizes (e.g. Cohen's *d*, Pearson's *r*), indicating how they were calculated

Our web collection on statistics for biologists contains articles on many of the points above.

Software and code

Policy information about availability of computer code

Data collection	No software was used for data collection.
Data analysis	Individual GWAS were conducted using either PLINK v2.0 or SAIGE v0.20. Meta-analyses were conducted by GWAMA v2.1 and v.2.2.2. Conditional analysis was conducted by FINEMAP v1.4. Variants were mapped to gene transcripts by Ensembl Variant Effect Predictor (VEP version 99, GRCh37), and to genes and tissues by FUMA v1.3.6. LDSC v1.0.0 was used to estimate and partition heritability, to estimate genetic correlations, and for LDSC-SEG analyses. MAGMA v1.09 was used to identify genes and gene sets. DEPICT v1.194 was used for tissue and gene set enrichment analyses. R (versions R3.4.0, R3.5.0 and R3.5.1) were used for PheWAS, for tissue enrichment analysis, for gene expression analyses and for subtype-specificity analyses (https://github.com/mjpirinen/migraine-meta). FOCUS v0.7 was used for fine-mapping causal gene sets. S-PrediXcan (from MetaXcan v0.7.5) was used for transcriptome-wide association study. COLOC v4.0.4 was used for colocalization analyses.

For manuscripts utilizing custom algorithms or software that are central to the research but not yet described in published literature, software must be made available to editors and reviewers. We strongly encourage code deposition in a community repository (e.g. GitHub). See the Nature Portfolio guidelines for submitting code & software for further information.

Data

Policy information about availability of data

All manuscripts must include a data availability statement. This statement should provide the following information, where applicable:

- Accession codes, unique identifiers, or web links for publicly available datasets
- A description of any restrictions on data availability
- For clinical datasets or third party data, please ensure that the statement adheres to our policy

Results for 8,117 genome-wide significant SNP associations ($P < 5 \times 10{-}8$) from the meta-analysis including 23andMe data are available on the International Headache Genetics Consortium website (http://www.headachegenetics.org/content/datasets-and-cohorts). Genome-wide summary statistics for the other study collections except 23andMe are available for bona fide researchers (contact Dr. Dale Nyholt, d.nyholt@qut.edu.au) within two weeks from the request. The full GWAS summary statistics for the 23andMe discovery data set will be made available through 23andMe to qualified researchers under an agreement with 23andMe that protects the privacy of the 23andMe participants. Please visit research.23andme.com/collaborate/#publication for more information and to apply to access the data.

Field-specific reporting

Please select the one below that is the best fit for your research. If you are not sure, read the appropriate sections before making your selection.

☒ Life sciences ☐ Behavioural & social sciences ☐ Ecological, evolutionary & environmental sciences

For a reference copy of the document with all sections, see nature.com/documents/nr-reporting-summary-flat.pdf

Life sciences study design

All studies must disclose on these points even when the disclosure is negative.

Sample size	Our total sample size is 102,084 migraine cases and 771,257 controls, and sample sizes for subtypes are 14,624 MA cases and 703,852 controls, and 15,055 MO cases and 682,301 controls. These sample sizes resulted when we included all available samples to maximize statistical power for GWAS discovery. Exact sample size was not predetermined by any other criterion except the availability of samples.
Data exclusions	We followed standard quality control procedures of GWAS to exclude individuals and genetic variants. Further details are described in the Methods section and in Supplementary Note.
Replication	High genetic correlations showed that the genetic architecture of migraine phenotype in different study collections was highly similar. We assessed the consistency across the study cohorts in subtype-specific analyses by sign tests.<br>We do not report a separate replication because we included all available data in the analyses to maximize the statistical power.
Randomization	Our study is a case-control study, and randomization is not applicable.
Blinding	Our study is a case-control study, and blinding is not applicable.

Reporting for specific materials, systems and methods

We require information from authors about some types of materials, experimental systems and methods used in many studies. Here, indicate whether each material, system or method listed is relevant to your study. If you are not sure if a list item applies to your research, read the appropriate section before selecting a response.

Materials & experimental systems

n/a	Involved in the study	
☒	☐	Antibodies
☒	☐	Eukaryotic cell lines
☒	☐	Palaeontology and archaeology
☒	☐	Animals and other organisms
☐	☒	Human research participants
☒	☐	Clinical data
☒	☐	Dual use research of concern

Methods

n/a	Involved in the study	
☒	☐	ChIP-seq
☒	☐	Flow cytometry
☒	☐	MRI-based neuroimaging

Human research participants

Policy information about studies involving human research participants

Population characteristics	All study participants are adult females or males of European descent. The migraine sample prevalence was 11.7% for the

Population characteristics	main meta-analysis (14.4% (IHGC2016), 18.7% (23andMe), 3.2% (UK Biobank), 18.2% (GeneRISK), 19.4% (HUNT)). MA sample prevalence was 2.0% and MO sample prevalence was 2.2%. Age distribution varied between studies. More detailed description of each study collections are provided in Supplementary Note, and for the UK Biobank in (https://www.ukbiobank.ac.uk).
Recruitment	The migraine GWAS meta-analysis consists of 5 study collections, and the subtype analyses included also 3 other study collections. Participants were recruited through population-based cohort studies, case-control studies, a biobank, a direct-to-consumer study, and through hospitals and clinics. For a majority of the cases, migraine phenotype was self-reported, but a subset of the patients were phenotyped in specialized headache centers. Further details of each study's recruitment are provided in the Supplementary Note.<br>We note that a large proportion of migraine diagnoses is self-reported. Therefore, it is possible that there are some cases among the controls. The consequence of this is that the observed differences in frequencies of migraine risk alleles between cases and controls are smaller, and we would have less statistical power, compared to more accurate control definition. However, in this scenario, the bias would be towards zero at the migraine risk variants, but null variants would not be biased. Further, we cannot rule out misdiagnosis, such as, e.g., tension headache being reported as migraine. The consequence of this would be that some of the risk loci could overemphasize genetic factors related to some other migraine-associated traits such as general pain mechanisms rather than genetic factors of migraine itself.<br>However, the high genetic correlation that we observed supports a strong phenotypic concordance between the study collections that include also deeply phenotyped clinical cohorts from headache specialist centers.
Ethics oversight	All participating studies were approved by local research ethics committees and written informed consent was obtained from all study participants. Further details are described in Supplementary Note.

Note that full information on the approval of the study protocol must also be provided in the manuscript.
