# Supplementary material for: Genome-wide analysis of 102,084 migraine cases identifies 123 risk loci and subtype-specific risk alleles
Source: Nat Genet. 2022 Feb 3;54(2):152–60. doi: 10.1038/s41588-021-00990-0 (PMC8837554; doi:10.1038/s41588-021-00990-0)
Supplement: Supplementary file 7 — Forest plots of 10 variants that have been previously reported to associate with migraine but failed to replicate in our study. [file 41588_2021_990_MOESM7_ESM.pdf]

Supplementary Data 3. **Forest plots of 10 variants that have been previously reported to have association with migraine but have *P*-value above genome-wide significance threshold ($P > 5 \times 10^{-8}$) in our study (N = 873,341; 102,084 migraine cases and 771,257 controls).** For each variant, we plot the log-odds-ratio estimate (BETA) with its 95%-confidence intervals (green) from each of the five studies included in the meta-analysis and a combined beta estimate from the fixed-effect meta-analysis (blue diamond). Grey squares indicate the sample sizes of each study. We annotate each plot with the lead variant and effect allele, uncorrected two-sided *P*-value by the inverse-variance weighted fixed-effect meta-analysis and the heterogeneity index (I^2).

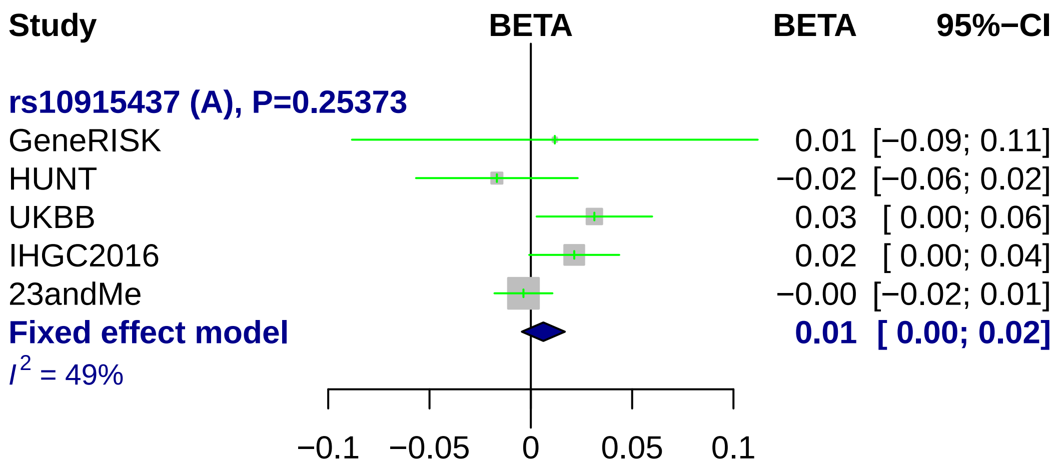
Study
BETA
BETA
95%−CI
rs10915437 (A), P=0.25373
GeneRISK
0.01 [−0.09; 0.11]
HUNT
−0.02 [−0.06; 0.02]
UKBB
0.03 [0.00; 0.06]
IHGC2016
0.02 [0.00; 0.04]
23andMe
−0.00 [−0.02; 0.01]
Fixed effect model
0.01 [0.00; 0.02]
I2 = 49%
−0.1
−0.05
0
0.05
0.1

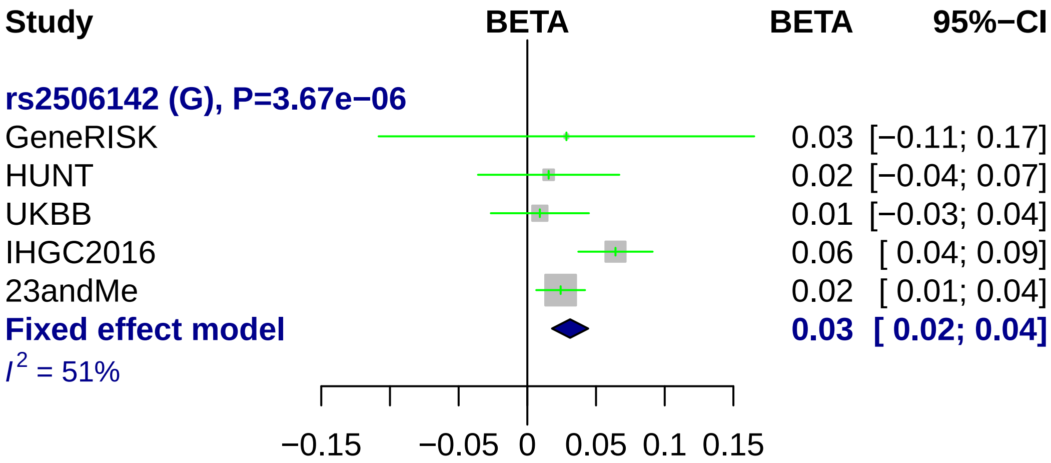
Study
BETA
BETA
95%−CI
rs2506142 (G), P=3.67e−06
GeneRISK
0.03 [−0.11; 0.17]
HUNT
0.02 [−0.04; 0.07]
UKBB
0.01 [−0.03; 0.04]
IHGC2016
0.06 [0.04; 0.09]
23andMe
0.02 [0.01; 0.04]
Fixed effect model
0.03 [0.02; 0.04]
I2 = 51%
−0.15
−0.05
0
0.05
0.1
0.15

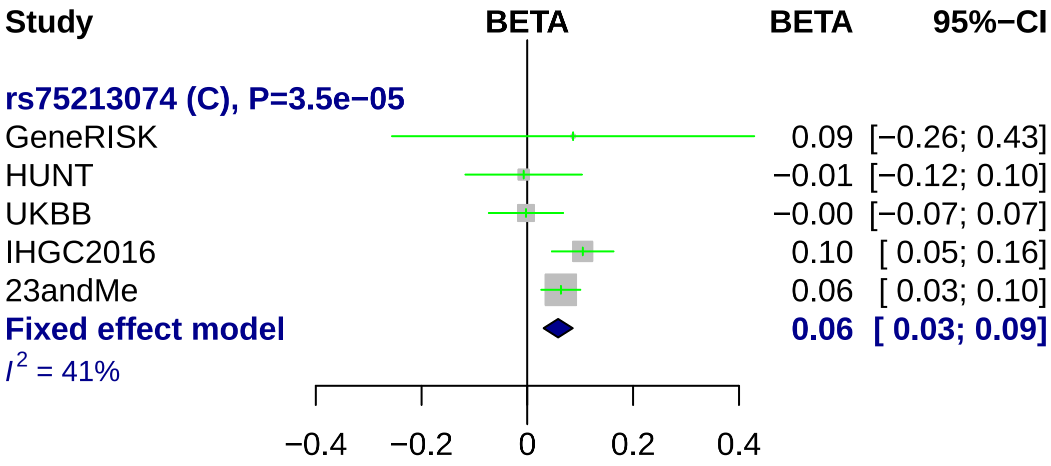
Study
BETA
BETA
95%−CI
rs75213074 (C), P=3.5e−05
GeneRISK 0.09 [−0.26; 0.43]
HUNT −0.01 [−0.12; 0.10]
UKBB −0.00 [−0.07; 0.07]
IHGC2016 0.10 [0.05; 0.16]
23andMe 0.06 [0.03; 0.10]
Fixed effect model 0.06 [0.03; 0.09]
I2 = 41%
−0.4
−0.2
0
0.2
0.4

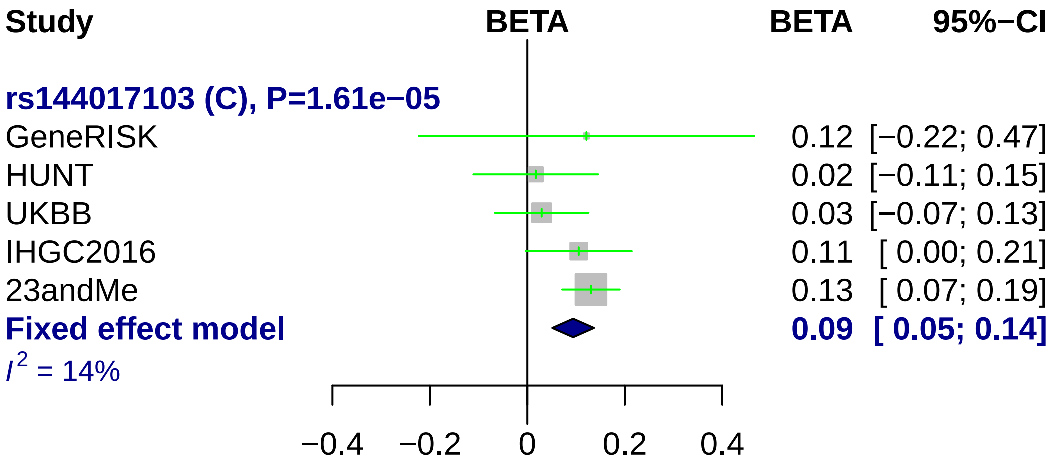
Study
BETA
BETA
95%−CI
rs144017103 (C), P=1.61e−05
GeneRISK
0.12 [−0.22; 0.47]
HUNT
0.02 [−0.11; 0.15]
UKBB
0.03 [−0.07; 0.13]
IHGC2016
0.11 [0.00; 0.21]
23andMe
0.13 [0.07; 0.19]
Fixed effect model
0.09 [0.05; 0.14]
I2 = 14%
−0.4
−0.2
0
0.2
0.4

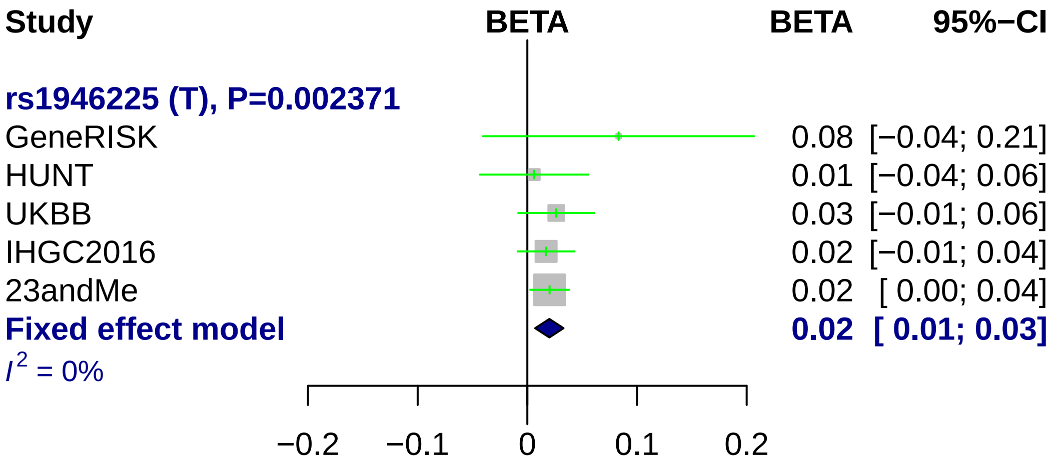

Study
BETA
BETA
95%−CI
rs1946225 (T), P=0.002371
GeneRISK 0.08 [−0.04; 0.21]
HUNT 0.01 [−0.04; 0.06]
UKBB 0.03 [−0.01; 0.06]
IHGC2016 0.02 [−0.01; 0.04]
23andMe 0.02 [0.00; 0.04]
Fixed effect model 0.02 [0.01; 0.03]
I2 = 0%
−0.2
−0.1
0
0.1
0.2

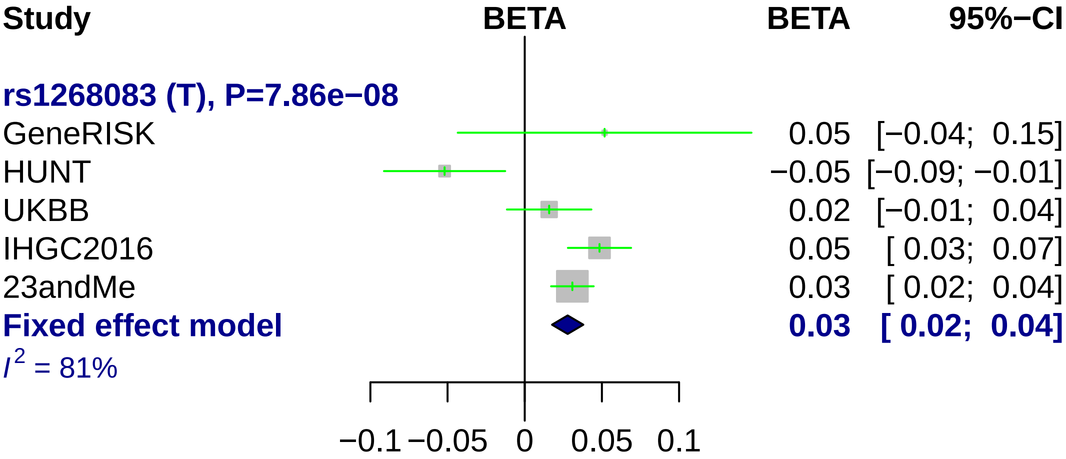
Study
BETA
BETA
95%−CI
rs1268083 (T), P=7.86e−08
GeneRISK 0.05 [−0.04; 0.15]
HUNT −0.05 [−0.09; −0.01]
UKBB 0.02 [−0.01; 0.04]
IHGC2016 0.05 [0.03; 0.07]
23andMe 0.03 [0.02; 0.04]
Fixed effect model 0.03 [0.02; 0.04]
I^2 = 81%
−0.1
−0.05
0
0.05
0.1

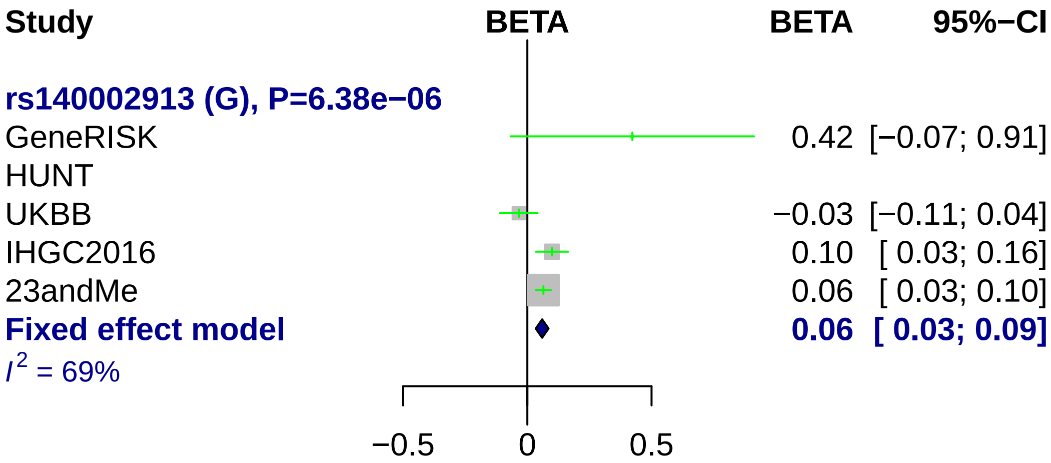
Study
BETA
BETA
95%−CI
rs140002913 (G), P=6.38e−06
GeneRISK
0.42 [−0.07; 0.91]
HUNT
UKBB
−0.03 [−0.11; 0.04]
IHGC2016
0.10 [0.03; 0.16]
23andMe
0.06 [0.03; 0.10]
Fixed effect model
0.06 [0.03; 0.09]
I2 = 69%
−0.5
0
0.5

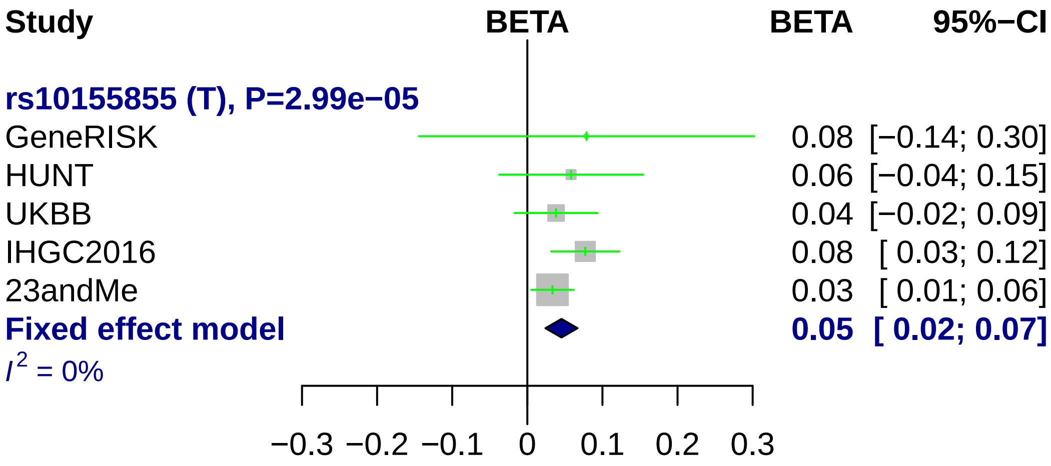
Study
BETA
BETA
95%−CI
rs10155855 (T), P=2.99e−05
GeneRISK
0.08 [−0.14; 0.30]
HUNT
0.06 [−0.04; 0.15]
UKBB
0.04 [−0.02; 0.09]
IHGC2016
0.08 [0.03; 0.12]
23andMe
0.03 [0.01; 0.06]
Fixed effect model
0.05 [0.02; 0.07]
I2 = 0%
−0.3
−0.2
−0.1
0
0.1
0.2
0.3

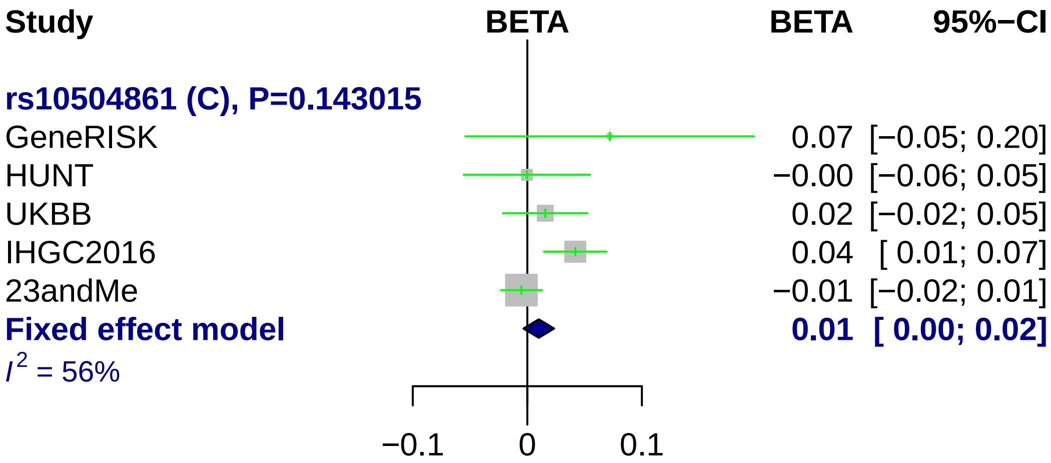
Study
BETA
BETA
95%−CI
rs10504861 (C), P=0.143015
GeneRISK
0.07 [−0.05; 0.20]
HUNT
−0.00 [−0.06; 0.05]
UKBB
0.02 [−0.02; 0.05]
IHGC2016
0.04 [0.01; 0.07]
23andMe
−0.01 [−0.02; 0.01]
Fixed effect model
0.01 [0.00; 0.02]
I^2 = 56%
−0.1
0
0.1

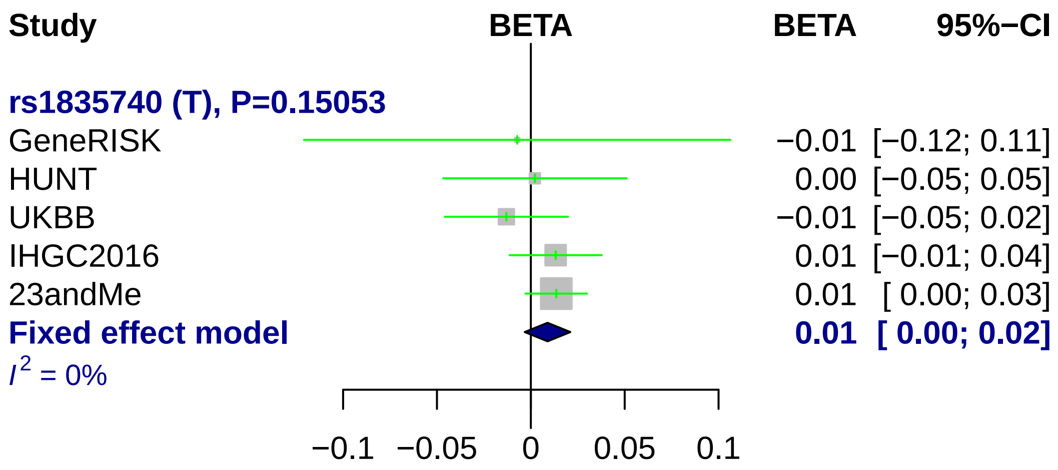

Study
BETA
BETA
95%−CI
rs1835740 (T), P=0.15053
GeneRISK −0.01 [−0.12; 0.11]
HUNT 0.00 [−0.05; 0.05]
UKBB −0.01 [−0.05; 0.02]
IHGC2016 0.01 [−0.01; 0.04]
23andMe 0.01 [0.00; 0.03]
Fixed effect model 0.01 [0.00; 0.02]
I2 = 0%
−0.1
−0.05
0
0.05
0.1
