# Supplementary material for: Genome-wide analysis of 102,084 migraine cases identifies 123 risk loci and subtype-specific risk alleles
Source: Nat Genet. 2022 Feb 3;54(2):152–60. doi: 10.1038/s41588-021-00990-0 (PMC8837554; doi:10.1038/s41588-021-00990-0)
Supplement: Supplementary file 3 — Peer Review Information. [file 41588_2021_990_MOESM3_ESM.pdf]

natureresearch

Peer Review Information

Journal: Nature Genetics
Manuscript Title: Genome-wide analysis of 102,084 migraine cases identifies 123 risk loci and subtype-specific risk alleles
Corresponding author name(s): Dr Matti Pirinen

Reviewer Comments & Decisions:

Decision Letter, initial version:

11th February 2021

Dear Matti,

Your Article "Genome-wide analysis of 102,084 migraine cases identifies 123 risk loci and subtype-specific risk alleles" has been seen by two referees. You will see from their comments below that, while they find your work of interest, they have raised some relevant points. We are interested in the possibility of publishing your study in Nature Genetics, but we would like to consider your response to these points in the form of a revised manuscript before we make a final decision on publication.

To guide the scope of the revisions, the editors discuss the referee reports in detail within the team, including with the chief editor, with a view to identifying key priorities that should be addressed in revision, and sometimes overruling referee requests that are deemed beyond the scope of the current study. In this case, we ask that you address all technical queries related to the primary association analyses and extend the fine-mapping and eQTL analyses as requested by Reviewer #1, and that you clarify the association evidence for all previously reported loci in the current analyses as requested by Reviewer #2. We hope that you will find this prioritized set of referee points to be useful when revising your study. Please do not hesitate to get in touch if you would like to discuss these issues further.

We therefore invite you to revise your manuscript taking into account all reviewer and editor comments. Please highlight all changes in the manuscript text file. At this stage we will need you to upload a copy of the manuscript in MS Word .docx or similar editable format.

We are committed to providing a fair and constructive peer-review process. Do not hesitate to contact us if there are specific requests from the reviewers that you believe are technically impossible or unlikely to yield a meaningful outcome.

When revising your manuscript:

*1) Include a “Response to referees” document detailing, point-by-point, how you addressed each referee comment. If no action was taken to address a point, you must provide a compelling argument. This response will be sent back to the referees along with the revised manuscript.

*2) If you have not done so already please begin to revise your manuscript so that it conforms to our Article format instructions, available
here.
Refer also to any guidelines provided in this letter.

*3) Include a revised version of any required Reporting Summary:
https://www.nature.com/documents/nr-reporting-summary.pdf
It will be available to referees (and, potentially, statisticians) to aid in their evaluation if the manuscript goes back for peer review.
A revised checklist is essential for re-review of the paper.

Please be aware of our guidelines on digital image standards.

Please use the link below to submit your revised manuscript and related files:

[REDACTED]

Note: This URL links to your confidential home page and associated information about manuscripts you may have submitted, or that you are reviewing for us. If you wish to forward this email to co-authors, please delete the link to your homepage.

We hope to receive your revised manuscript within 8-12 weeks. If you cannot send it within this time, please let us know.

Please do not hesitate to contact me if you have any questions or would like to discuss these revisions further.

Nature Genetics is committed to improving transparency in authorship. As part of our efforts in this direction, we are now requesting that all authors identified as ‘corresponding author’ on published papers create and link their Open Researcher and Contributor Identifier (ORCID) with their account on the Manuscript Tracking System (MTS), prior to acceptance. ORCID helps the scientific community achieve unambiguous attribution of all scholarly contributions. You can create and link your ORCID from the home page of the MTS by clicking on ‘Modify my Springer Nature account’. For more information please visit please visit www.springernature.com/orcid.

We look forward to seeing the revised manuscript and thank you for the opportunity to review your work.

Sincerely,

natureresearch

Kyle

Kyle Vogan, PhD
Senior Editor
Nature Genetics
https://orcid.org/0000-0001-9565-9665

Referee expertise:

Referee #1: Genetics, neurological diseases

Referee #2: Genetics, neurological diseases, vascular diseases

Reviewers' Comments:

Reviewer #1:
Remarks to the Author:

Hautakangas et al. have written an interesting manuscript on migraine, with the current largest cohort for migraine. They found novel genes and loci implicated in the phenotype. Although the question is important, there are many methodological concerns this reviewer has that the authors need to address.

1. Can the authors justify why they chose EAF of 0.30 and 0.20 for the UK biobank? Can they cite some papers?

2. Not excluding variants due to strand ambiguity may be too relaxed, despite assessing whether there is MAF concordance.

3. During meta-analysis, there was no mention of converting linear-mixed model results using SAIGE back to logistic. The authors wrote generalized "logistic" mixed model, but it is linear and the beta and standard error need to be transformed back in order to meta-analyze logistic addictive results from the other cohorts and the SAIGE datasets. This may have skewed the results.

4. Why did authors decide on Neff <2500? 2500 is a very low number for filtering on effective sample size. It should be 70% of the total N effective as previously described and used in other papers. For calculating effective sample size, they should use this formula instead: Winkler, T. W. et al. Quality control and conduct of genome-wide association meta-analyses. Nat. Protocols 9, 1192–1212 (2014).

5. GWS is not commonly used as an acronym for genome-wide significant. Easily mixed up with GWAS.

6. Can the authors provide a citation that demonstrates stepwise CA is more robust to mismatches for fine-mapping? And whether using the LD structure of the UK biobank is okay despite the UK Biobank constitution a large portion of the GWAS data?

7. For eQTL analyses, can the authors do a transcriptome-wide association study with FUSION or Predixcan? And fine-map those results for candidate genes with FOCUS.

8. An SMR approach for eQTLs would also improve the mapping section as opposed to arbitrarily assigning to GTEx genes.

9. For FUMA did they include +/- 1 kb of genes when doing mapping?

10. Why did authors pick INFO 0.6 for LDSC, and exclude strand ambiguous here but not in the results?

11. Can the authors provide more information on the intercept for LDSC and other metrics? Did they assess the percentage of stratification from the other LDSC metric?

12. For stratified LDSC, the 24 main functional categories baseline model is not the most recent / recommended to use.

13. Since the authors have UK Biobank data, can they do a PheWAS with that cohort instead of NHGRI since the latter is less consistent?

14. The authors did not talk about heterogeneity across the genome-wide significant loci. Did they calculate a Q statistic?

15. They should calculate polygenic risk and genetic correlation between cohorts to show there is meaningful genetic overlap and justify meta-analyzing. Especially with self-reported cohorts. The 2018 ADHD GWAS has detailed methodology on this.

16. Can the authors provide a scree plot to justify why they took only the first 4 principal components when adjusting for population structure?

Overall, the methodology is lacking and could be a lot more clear. There seems to be pertinent modern GWAS methodology that is missing from this paper. I'm unable to comment thoroughly on the results as they may potentially diverge due to lack of transformation for linear mixed model results.

Reviewer #2:
Remarks to the Author:

Current genome-wide association study (GWAS) with large sample size contained 102,084 migraine cases and 771,257 controls. The result identified 123 loci of which 86 are novel. The new risk loci include genes encoding recent migraine-specific drug targets, namely calcitonin gene-related peptide (CALCA/CALCB) and serotonin 1F receptor (HTR1F). The loci provide an opportunity to evaluate shared and distinct genetic components in the two main migraine subtypes (MA and MO). Overall, genomic annotations among migraine-associated variants were enriched in both vascular and central nervous system tissue/cell types supporting unequivocally that neurovascular mechanisms underlie migraine pathophysiology. Though there were so many novel loci identified, the mechanism of

migraine was still elusive.

1. Migraine is a common disease with a lifetime prevalence of 15-20%. So, how to differentiate the controls without migraine from general population? It's too difficult to screen all migraine individuals from the controls. I'm afraid that the results may be with bias by this mixture.

2. The authors conducted a GWAS meta-analysis of migraine by adding to the previous meta-analysis of Gormley et al. (2016) from four study collections: 23andMe, UKBB, GeneRISK and HUNT. However the cohort of 23andMe was also included in the previous meta-analysis of Gormley et al. (2016). It's confusing for readers.

3. The previous meta-analysis of Gormley et al. (2016) with 59,674 cases and 316,078 controls identified 38 loci. 19 of 38 loci were identified in this GWAS, how about the remaining loci in this study?

4. Previously, Gormley et al. (2016) conducted subtype-specific GWAS reported that 7 loci were GWS in MO but none was GWS in MA. In current GWAS, three SNPs were MA-specific, two SNPs were MO-specific and nine SNPs were shared. How about the previously reported 7 loci MO-specific in current study?

5. The authors stated that the new risk loci include genes encoding recent migraine-specific drug targets, namely calcitonin gene-related peptide (CALCA/CALCB) and serotonin 1F receptor (HTR1F). Since the drug targets were reported, the contribution of the identification of CALCA/CALCB and HTR1F was limited.

6. With no doubt, the group indeed identified many novel loci for migraine from the previous meta-analysis of Gormley et al. (2016) to current GWAS. One day, some of these loci may explain the mechanism of the development of migraine, however, till now, the mechanism was elusive based on so many identification. Using GWAS was unlikely to explore the mechanism of migraine. More work should be done.

Author Rebuttal to Initial comments

Dear Matti,

Your Article "Genome-wide analysis of 102,084 migraine cases identifies 123 risk loci and subtype-specific risk alleles" has been seen by two referees. You will see from their comments below that, while they find your work of interest, they have raised some relevant points. We are interested in the possibility of publishing your study in Nature Genetics, but we would like to consider your response to these points in the form of a revised manuscript before we make a final decision on publication.

To guide the scope of the revisions, the editors discuss the referee reports in detail within the team, including with the chief editor, with a view to identifying key priorities that should be addressed in revision, and sometimes overruling referee requests that are deemed beyond the scope of the current

study. In this case, we ask that you address all technical queries related to the primary association analyses and extend the fine-mapping and eQTL analyses as requested by Reviewer #1, and that you clarify the association evidence for all previously reported loci in the current analyses as requested by Reviewer #2. We hope that you will find this prioritized set of referee points to be useful when revising your study. Please do not hesitate to get in touch if you would like to discuss these issues further.

Dear Kyle,

Thank you and the editorial team for your guidance with the revision.

We have addressed all technical queries related to the primary GWAS and extended the fine-mapping and eQTL analyses as requested by Reviewer 1. In particular, we have included new results from two gene prioritization approaches suggested by Reviewer 1. We have also clarified the association evidence for previously reported loci as requested by Reviewer 2 both in the main manuscript and in Supplementary Table 4.

We hope that with these additions to our manuscript you will find the revised work suitable for publication in *Nature Genetics*.

With kind regards,

Heidi Hautakangas and Matti Pirinen on behalf of the International Headache Genetics Consortium

Response to reviewers:

Reviewer #1:

1. Can the authors justify why they chose EAF of 0.30 and 0.20 for the UK biobank? Can they cite some papers?

Response: We chose the thresholds for these variant discrepancy filters by visually inspecting the pairwise effect allele frequency (EAF) plots (Supplementary Figure 13) as recommended, for example, by Winkrel et al. (2014) to identify problems due to allele miscoding or strand flips between cohorts. Since mismatches in allele coding between cohorts are not expected to cause false-positive associations, we removed only the obviously problematic variants at this filtering step. By allowing EAF differences up to 0.3 for SNPs and up to 0.2 for indels we removed 0.02% of our SNPs and 0.6% of our indels. To further confirm that our thresholds are not unnecessarily removing good quality variants, we checked that in 1000 Genomes data (chromosome 10) less than 0.0002% had a discrepancy > 0.3 between CEU (Central and Northern European Ancestry) and FIN (Finnish) populations that represent the largest variation in allele frequencies among our study collections. We chose a stricter filter for the indel data since we expect more issues with indel calling compared to SNP calling.

Reference:
- Winkrel et al. (2014) Quality control and conduct of genome-wide association meta-analyses. *Nature Protocols* 9:1192–1212.

2. Not excluding variants due to strand ambiguity may be too relaxed, despite assessing whether there is MAF concordance.

Response: We agree that it is not possible to completely exclude the possibility of any strand flips for variants whose MAF is close to 50%. However, since strand flips between cohorts would not generate false-positive associations, and since our sensitivity analyses showed no evidence of problems with strand-ambiguous SNPs, we did not see the need to remove all C-G or A-T SNPs from the analyses.

We explain our reasoning on page 20 as follows: *"We conducted a sensitivity analysis on strand-ambiguous SNPs (with alleles A/T or G/C), by counting, for each pair of studies, how often the same allele of A/T or G/C SNP was coded as the minor allele in both cohorts, as a function of MAF threshold (Supplementary Table 17). Minor alleles were the same at least in 97.39% of the SNPs without MAF threshold and the corresponding proportions were 99.96% and 79.58% when MAF < 0.25 and when MAF > 0.4, respectively. The very high concordance for SNPs with MAF < 0.25 suggests that the strand-ambiguous SNPs were consistently labelled for almost every SNP. Therefore, we did not exclude any SNPs based on possible labelling mismatches due to strand ambiguity."*.

3. During meta-analysis, there was no mention of converting linear-mixed model results using SAIGE back to logistic. The authors wrote generalized "logistic" mixed model, but it is linear and the beta and

standard error need to be transformed back in order to meta-analyze logistic addictive results from the other cohorts and the SAIGE datasets. This may have skewed the results.
Response: SAIGE implements both linear mixed model and logistic mixed model (Zhou et al. 2018). The trait type is indicated by a flag –traitType that can take values 'binary' (logistic mixed model) or 'quantitative' (linear mixed model). We applied SAIGE only to the HUNT cohort. For HUNT, we used the binary option (--traitType=binary) and therefore our betas and standard errors for HUNT are on the log-odds scale just like they are for our other cohorts.

To make clearer to the reader what we did we added the following statement to the main text (page 21): *"For HUNT data, we used a logistic mixed model with the saddlepoint approximation as implemented in SAIGE v0.20 (Zhou et al., 2018) that accounts for the genetic relatedness.".*

In addition, we added a statement in Supplementary Note 1 (page 4): *"Association analyses were conducted using SAIGE v0.20 (Zhou et al., 2018), a logistic mixed effects model approach, to account for cryptic population structure and relatedness when modelling the association between genotype probabilities (dosages) and binary migraine phenotype.".*

Reference:
- Zhou et al. (2018) Efficiently controlling for case-control imbalance and sample relatedness in large-scale genetic association studies. *Nature Genetics* 50:1335–1341.

4. Why did authors decide on Neff <2500? 2500 is a very low number for filtering on effective sample size. It should be 70% of the total N effective as previously described and used in other papers. For calculating effective sample size, they should use this formula instead: Winkler, T. W. et al. Quality control and conduct of genome-wide association meta-analyses. Nat. Protocols 9, 1192–1212 (2014).
Response: Thank you for pointing out this issue. The effective sample size as defined in Winkler et al. is:

$$N_{eff} = \frac{2}{\left(\frac{1}{S} + \frac{1}{R}\right)} = 2\,\frac{S \times R}{S + R} = 2\,\frac{S \times R}{N} = 2\,N\,\frac{S}{N}\frac{R}{N} = 2N\varphi(1-\varphi) = 2M,$$

where S is the number of cases, R is the number of controls, $N = S + R$ is the total sample size, φ is the proportion of cases and M is the quantity that we used to call effective sample size in our first submission. In other words, the definition of Winkler et al. differs from ours by a constant multiplier of 2. We agree that the interpretation of the value defined by Winkler et al. is more intuitive than ours. According to Winkler et al., a case-control study with 5000 cases and 5000 controls has an effective sample size of 5000, while with our previous definition, such a study had an effective sample size of 2500. Hence, we now have revised the Methods section accordingly so our definition of effective sample size is the same as in Winkler et al.

We state on page 21: *"We estimated the effective sample size for variant i as* $N_{eff(i)} = \frac{1}{f_i(1-f_i)\,se_i^2}$*, where* f_i *is the effect allele frequency for variant i and* se_i *is the standard error estimated by the GWAS software.*

This quantity approximates the value 2 N t (1-t) I, where N is the total sample size (cases + controls), t is the proportion of cases and I is the imputation info (derivation in Supplementary Note 1).".

In addition, we have added to the Supplementary Note 1 (page 11): "*We note that our definition of N_{eff} matches exactly with the definition of effective sample size given by Winkler et al. (2014) for perfectly observed variants with info value of $I_i = 1$: They write the formula as $N_{eff} = 2/\left({}^{1}/_{N\phi} + {}^{1}/_{N(1-\phi)}\right)$ without an extension to imputed genotypes where $I_i < 1$.".*

Consequently, the minimum sample size for which we report results is now $N_{eff} = 5000$. Our threshold N_{eff} = 5000 filters out variants that are present only in the smallest cohort (GeneRISK with N_{eff} = 1772), or that otherwise were present in a very small number of individuals compared to our full meta-analysis data. We filter such results out because they are imprecise compared to the bulk of the meta-analysis results that came from cohorts with much larger sample sizes.

The effective sample sizes of our cohorts are:

GeneRISK	1772
HUNT	12,576
UKBB	21,068
IHGC2016	49,977
23andMe	86,354

We agree that many downstream analyses must use stricter filtering, and we have done strict filtering for the conditional and colocalization analyses for which we have included only variants with ± 10% of the N_{eff} of the lead variant (as explained at pages 24 and 32). However, we feel that it is most useful for the reader and the researchers who will want to use the data in the future that we provide a longer list of variants that each researcher themselves can filter down depending on their specific needs using the effective sample size value that we report. We added the effective sample size to Supplementary Table 3a.

5. GWS is not commonly used as an acronym for genome-wide significant. Easily mixed up with GWAS.
Response: We agree that GWS is not a very widely used acronym, but it is in current use also in *Nature Genetics* (Kurilshikov et al. 2021). We feel that the two acronyms can be used next to each other without causing confusion, as long as the acronyms are clearly defined; "GWAS" and "GWS" are defined on pages 5 and 9, respectively. The alternative, to use the non-abbreviated wordy 'genome-wide significant', we feel is less appealing, also because it is used no less than 25 times.

Reference:

- Kurilshikov et al. (2021). Large-scale association analyses identify host factors influencing human gut microbiome composition. *Nature Genetics* 53: 156–165.

6. Can the authors provide a citation that demonstrates stepwise CA is more robust to mismatches for fine-mapping? And whether using the LD structure of the UK biobank is okay despite the UK Biobank constitution a large portion of the GWAS data?

Response: The highest false-positive signals are observed at those variant combinations that have the largest mismatches between available LD and GWAS results (Benner et al. 2017). Chances of running into larger mismatches increase with the number of variant combinations that are evaluated during the analysis. Thus, our statement that CA is more robust to mismatches in LD than fine-mapping is based on the fact that fine-mapping evaluates many times more variant combinations than stepwise CA. For example, if a region contains M variants and we allow for K causal variants, CA goes through approximately MK variant combinations, whereas fine-mapping goes through approximately M^K variant combinations.

We have now explicitly stated why CA is more robust to mismatches in LD than fine-mapping in the Methods section (page 23): "*When the reference LD does not accurately match the GWAS data, full fine-mapping is prone to false-positives (Benner et al., 2017). A simpler stepwise CA is more robust to inaccuracy in reference LD because CA has a much smaller search space than full fine-mapping, and therefore CA is less likely to run into most problematic variant combinations where LD is very inaccurate. Since we did not have the full in-sample LD from our GWAS data, we only carried out the CA and not the full fine-mapping.*".

The fact that UKB is included in the meta-analysis is not a problem for using UKB as a reference LD panel for conditional analysis. Actually, in an ideal case, the LD for CA would be computed from the full GWAS data used in the meta-analysis (Benner et al. 2017) but since we do not have access to individual-level data from all study collections, we had to restrict our LD reference to UKB.

Reference:
- Benner et al. (2017) Prospects of Fine-Mapping Trait-Associated Genomic Regions by Using Summary Statistics from Genome-wide Association Studies. *American Journal of Human Genetics* 101: 539–551.

7. For eQTL analyses, can the authors do a transcriptome-wide association study with FUSION or Predixcan? And fine-map those results for candidate genes with FOCUS.

Response: We thank the Reviewer for the good suggestions. We have now performed a transcriptome-wide association study using S-PrediXcan (Barbeira et al. 2018), i.e. a summary statistics version of PrediXcan using expression weights from GTEx v8 data. Furthermore, as suggested by the authors of S-PrediXcan (Barbeira et al. 2018), we combined the S-PrediXcan analysis with a colocalization using COLOC (Gianbartolomei et al. 2014) to prioritize candidate genes. As suggested by the Reviewer, we additionally fine-mapped the candidate genes using FOCUS (Mancuso et al. 2019) with GTEx v8 data.

While the addition of the two approaches improved our manuscript, we are aware of the same problems here that complicate variant-level fine-mapping, namely, varying effective sample size between variants and variation in LD structure between GTEx data and our full meta-analysis. Furthermore, computational gene prioritization using summary statistics would still require further work to become robust (Wainberg

et al. 2019). Therefore, we have kept the discussion of the results brief in the main manuscript (page 11): "*To prioritize candidate genes for the risk loci, we applied two approaches based on GTEx v8 expression data: fine-mapping of causal gene-sets by FOCUS (Mancuso et al., 2019) (Supplementary Table 11a) and a transcriptome-wide association study (TWAS) by S-PrediXcan (Barbeira et al., 2018) combined with colocalization analysis using COLOC (Giambartolomei et al., 2014) (Supplementary Table 11b). With posterior probability (PP) > 0.5, FOCUS found candidate genes for 82 loci and S-PrediXcan + COLOC supported colocalization for 52 loci (Supplementary Table 11c). In total 73 genes in 46 loci were prioritized by both methods prioritized. MRC2 and PHACTR1 were the only genes that both methods prioritized with strong evidence (PP > 0.99 for same tissue) and without any other gene prioritized within their loci.*".

Details of how the analyses were performed are now included in the Methods section (new subsections *Transcriptome-wide association study (TWAS) and colocalization with S-PrediXcan and COLOC* on page 31 and *Fine-mapping of causal gene sets (FOCUS)* on page 32).

In addition, we have discussed the challenges in the Discussion section (page 18), as follows: "*Even though we observed links between our new risk loci and known target genes of effective migraine drugs, the accurate gene prioritization at risk loci remains challenging. First, robust fine-mapping would require accurate LD information (Benner et al. 2017), which is typically lacking in meta-analyses and further distorted from reference panels by variation in effective sample size across variants. Second, computational approaches to gene prioritization require further methodological work (Wainberg et al. 2019) and extension to additional sources of functional data in order to provide more robust and comprehensive gene prioritization results.*".

References:

- Barbeira et al. (2018) Exploring the phenotypic consequences of tissue specific gene expression variation inferred from GWAS summary statistics. *Nature Communications* 9: 1825.
- Gianbartolomei et al. (2014) Bayesian test for colocalisation between pairs of genetic association studies using summary statistics. *PLoS Genetics* 10: e1004383.
- Mancuso et al. (2019) Probabilistic fine-mapping of transcriptome-wide association studies. *Nature Genetics, 51*(4), 675-682.
- Wainberg et al. (2019) Opportunities and challenges for transcriptome-wide association studies. *Nature Genetics* 51: 592–599.

8. An SMR approach for eQTLs would also improve the mapping section as opposed to arbitrarily assigning to GTEx genes.

Response: We have now improved the mapping to candidate genes by two gene prioritization approaches (S-PrediXcan + COLOC and FOCUS) as suggested above by this Reviewer. SMR is a closely related method to S-PrediXcan in the sense that both estimate the association between intermediate gene expression levels and phenotypes (Zhu and Zhou, 2020) but recent work (Barbeira et al. 2018; Yuan et al. 2020) has reported that SMR is not calibrated as reliably as S-PrediXcan. Hence, we considered that the combination

of S-PrediXcan + COLOC and FOCUS brings the most up-to-date addition to the manuscript in terms of current computational methods that map GWAS results to candidate genes.

References:
- Barbeira et al. (2018) Exploring the phenotypic consequences of tissue specific gene expression variation inferred from GWAS summary statistics. *Nature Communications* 9:1825.
- Zhu, H and Zhou X. (2020). Transcriptome-wide association studies: a view from Mendelian randomization. Quantitative Biology *https://doi.org/10.1007/s40484-020-0207-4*
- Yuan et al. (2020) Testing and controlling for horizontal pleiotropy with probabilistic Mendelian randomization in transcriptome-wide association studies. *Nature Communications* 11:3861.

9. For FUMA did they include +/- 1 kb of genes when doing mapping?
Response: We did the physical mapping between variants and genes using VEP where we used both ±20 kb window of genes and also a larger window size of ±250 kb. The results are reported in Supplementary Table 3a.

We used FUMA v1.3.6. only to map the lead variants and variants in high LD ($r^2 > 0.6$) with the lead variants to eQTLs. As input to FUMA, we included only the variants to be mapped, not the full genome-wide summary statistics, and we chose the option 'Use only significant snp-gene pairs' that uses only eQTLs with FDR ≤ 0.05. In FUMA, this eQTL mapping is based solely on position, that is, whether the variant is a significant eQTL in a specific tissue. This approach is now explained on page 25 and reads: *"With FUMA v1.3.6 (Watanabe et al., 2017), we mapped the 123 lead variants, and the variants in high LD ($r^2 > 0.6$) with the lead variants, to the other eQTL data repositories provided by FUMA except GTEx."*.

10. Why did authors pick INFO 0.6 for LDSC, and exclude strand ambiguous here but not in the results?
Response: For our LDSC analyses, we used EUR LD Score reference panel merged with the HapMap3 variants as recommended by Bulik-Sullivan et al. (2016). The authors of LDSC (Bulik-Sullivan et al. 2016) also wrote that additional variant filtering based on INFO is not necessary when this merging step with HapMap3 variants is applied. However, our meta-analysis summary statistics are reported only for variants that satisfy INFO > 0.6 and MAF > 0.01. Hence, the criterion INFO > 0.6 was not a threshold specific to LDSC but a threshold specific for whole meta-analysis. To avoid confusion, we have now excluded the explicit criterion of INFO > 0.6 from LDSC section.

The software package LDSC removes the strand-ambiguous variants by default, and hence they were removed from our LDSC analysis.

As we explained in our answer to the second remark of this Reviewer, we did not exclude the strand-ambiguous variants from our meta-analysis results because there was a very high concordance between the study collections in allele labeling, and therefore we expect few problems with strand flips.

Reference: Bulik-Sullivan et al. (2016) LD Score regression distinguishes confounding from polygenicity in genome-wide association studies. *Nature Genetics, 47*(3), 291-295.

11. Can the authors provide more information on the intercept for LDSC and other metrics? Did they assess the percentage of stratification from the other LDSC metric?

Response: We have now added to the legend of Supplementary Figure 2 the following text: *"The univariate LDSC intercept was 1.05 (s.e. 0.01) and the LDSC ratio between the intercept and mean χ^2-statistics was 0.078, suggesting that 92.2% of the observed inflation in χ^2-statistics is due to polygenicity of migraine*.".

12. For stratified LDSC, the 24 main functional categories baseline model is not the most recent/recommended to use.

Response: We thank the Reviewer for pointing this out. We now reran S-LDSC using more recent baseline-LD model (Gazal et al., 2017). We added the following statement to the Methods section (page 26): *"We used the baseline-LD model (Gazal et al., 2017) that contains 75 annotations including conserved, coding and regulatory regions of the genome and different histone modifications. Baseline-LD model adjusts for MAF- and LD-related annotations, such as recombination rate and predicted allele age, which decreases the risk of model misspecification (Finucane et al., 2015), (Gazal et al., 2017), (Hujoel et al., 2019).".*

Even though our analysis now includes 75 annotations, following the example of Jiang et al. (2019), we still considered only 24 unique functional annotations without the flanking regions when we tested for significance.

We note that while there is even more recent version v2.2 of S-LDSC available (Hujoel et al. 2019), to our knowledge, it is not being used for assessing the enrichment across all annotations, possibly because many of its annotations are highly correlated with each other. Therefore, we chose to use a well-established version of S-LDSC for the purpose of evaluating a comprehensive set of independent annotations.

References:
- Gazal et al. (2017) Linkage disequilibrium–dependent architecture of human complex traits shows action of negative selection. *Nature Genetics, 49*(10): 1421-1427.
- Finucane et al. (2015) Partitioning heritability by functional annotation using genome-wide association summary statistics. *Nature Genetics, 47*(11), 1228-1235.
- Hujoel et al. (2019) Disease Heritability Enrichment of Regulatory Elements Is Concentrated in Elements with Ancient Sequence Age and Conserved Function across Species. *American Journal of Human Genetics* 104:611–624.
- Jiang et al. (2019) Shared heritability and functional enrichment across six solid cancers. *Nature Communications* 10:431.

13. Since the authors have UK Biobank data, can they do a PheWAS with that cohort instead of NHGRI since the latter is less consistent?

Response: We feel that a PheWAS using GWAS Catalog is still useful even though we agree with the Reviewer that there are a wide variety of studies with respect to quality and sample size there. In addition to the GWAS Catalog, we have done a PheWAS using a single biobank data set from the FinnGen project with 2,263 disease phenotypes. While we have access to the migraine phenotype data on UKB, we do not have available PheWAS results from UKB, and feel that running/gathering PheWAS results for the whole of UKB is beyond the scope of the current migraine GWAS.

14. The authors did not talk about heterogeneity across the genome-wide significant loci. Did they calculate a Q statistic?
Response: To complete the results, as suggested by the Reviewer, we have now added both the Cochran´s Q and I^2 to Supplementary Table 3a.

15. They should calculate polygenic risk and genetic correlation between cohorts to show there is meaningful genetic overlap and justify meta-analyzing. Especially with self-reported cohorts. The 2018 ADHD GWAS has detailed methodology on this.
Response: We agree with the Reviewer that it is important to assess the genetic overlap between the study collections. We did that using bivariate LDSC. These results are reported in Supplementary Table 2 and at the start of the Results section (page 8) that reads: "*In spite of different approaches to the ascertainment of migraine cases across the studies, the pairwise genetic correlations were all near 1 (Supplementary Table 2), as determined by LD Score (LDSC) regression (Bulik-Sullivan et al., 2015a), showing high genetic and phenotypic similarity across the studies justifying their meta-analysis. Pairwise LDSC intercepts were all near 0, indicating little or no sample overlap (Supplementary Table 2).".*

16. Can the authors provide a scree plot to justify why they took only the first 4 principal components when adjusting for population structure?
Response: When it comes to the number of PCs, HUNT was the only study where we used only 4 principal components, but there we applied SAIGE with logistic mixed model that additionally accounts for population structure and genetic relationships via the full genetic relatedness matrix. UKBB and GeneRISK studies both used 10 principal components and 23andMe used 5 principal components in their analyses.

As a confirmation that population stratification does not pose a problem, the LDSC intercept did not show an elevated value that would indicate stratification problems, as stated on page 8 as follows: *"The univariate LDSC (Bulik-Sullivan et al., 2015b) intercept was 1.05 (s.e. 0.01), which, being close to 1.0, suggests that most of the genome-wide elevation of the association statistics comes from true additive polygenic effects rather than from a confounding bias such as population stratification.".*

Individual GWAS have been performed by different groups over the time span of last decade or so, and, therefore, we are not able to generate scree plots of the individual cohorts.

Overall, the methodology is lacking and could be a lot more clear. There seems to be pertinent modern GWAS methodology that is missing from this paper. I'm unable to comment thoroughly on the results as they may potentially diverge due to lack of transformation for linear mixed model results.
Response: We hope that the additional analyses and rewriting of the Methods section have sufficiently addressed the concerns raised.

Reviewer #2:

Current genome-wide association study (GWAS) with large sample size contained 102,084 migraine cases and 771,257 controls. The result identified 123 loci of which 86 are novel. The new risk loci include genes encoding recent migraine-specific drug targets, namely calcitonin gene-related peptide (CALCA/CALCB) and serotonin 1F receptor (HTR1F). The loci provide an opportunity to evaluate shared and distinct genetic components in the two main migraine subtypes (MA and MO). Overall, genomic annotations among migraine-associated variants were enriched in both vascular and central nervous system tissue/cell types supporting unequivocally that neurovascular mechanisms underlie migraine pathophysiology. Though there were so many novel loci identified, the mechanism of migraine was still elusive.

1. Migraine is a common disease with a lifetime prevalence of 15-20%. So, how to differentiate the controls without migraine from general population? It's too difficult to screen all migraine individuals from the controls. I'm afraid that the results may be with bias by this mixture.
Response: We agree with the Reviewer that it is likely that our controls include some migraineurs since, for many cohorts, the phenotype is based on self-reported information about the migraine status. The consequence of such mixture is that observed differences in frequencies of migraine risk alleles between cases and controls are smaller than what they would have been with more accurate control definition. Thus, the possible bias would be towards zero at the migraine risk variants but, importantly, there would not be any bias at the null variants. In other words, by including some migraineurs among our set of controls, we would have less statistical power than in an ideal scenario, but we would not suffer from false positives.

2. The authors conducted a GWAS meta-analysis of migraine by adding to the previous meta-analysis of Gormley et al. (2016) from four study collections: 23andMe, UKBB, GeneRISK and HUNT. However the cohort of 23andMe was also included in the previous meta-analysis of Gormley et al. (2016). It's confusing for readers.
Response: We thank the Reviewer for pointing this out. Throughout our study, we defined the "IHGC2016" data set as being devoid of 23andMe data, whereas we have included all 23andMe data that was previously used in Gormley et al. (2016) in our "23andMe" data set. We have now added a note to describe this better in Table 1 and we have also explicitly mentioned this in our cohort description in Supplementary Note 1 (page 1) that reads: *"23andMe cohort includes 220,876 controls and 53,109*

migraine cases, of which 30,465 were included in the previous meta-analysis of Gormley et al. (2016) and 22,644 are new cases.".

3. The previous meta-analysis of Gormley et al. (2016) with 59,674 cases and 316,078 controls identified 38 loci. 19 of 38 loci were identified in this GWAS, how about the remaining loci in this study?
Response: We thank the Reviewer for asking for clarification about this issue. We would like to refer the Reviewer to Supplementary Table 4 that presents the results of 48 previously reported migraine risk variants. In that Table we assign only 19 of the previously reported migraine risk variants to the study of Gormley et al. (2016) because the remaining 19 (being 38 – 19) genome-wide significant loci reported by Gormley et al. (2016) had been already reported before in Chasman et al. (2011), Freilinger et al. (2012), Anttila et al. (2013), or Pickrell et al. (2016). Altogether, in our study we observed genome-wide significant (GWS) *P*-values for 32 of the 38 loci that were reported as genome-wide significant by Gormley et al. (2016).

We have now added to Supplementary Table 4 a column "Reported by Gormley et al." that indicates the 38 loci that were reported by Gormley et al. (2016). We describe the column as: *"Reported by Gormley et al.: Indicator identifying the 38 loci reported as genome-wide significant by Gormley et al. (2016) (1 = yes, 0 = no).".*

For the remaining 6 (out of 38) loci from Gormley et al. (2016), *P*-values in our study did not reach the genome-wide significance threshold of 5×10^{-8}. We have provided *P*-values for these variants in Supplementary Table 4 and we also report them in the table below.

CHR	RSID	Gormley et al. *P*-value	Our *P*-value
6	rs140002913	4.0×10^{-8}	6.4×10^{-6}
6	rs1268083	5.0×10^{-9}	7.9×10^{-8}
7	rs10155855	2.0×10^{-8}	3.0×10^{-5}
10	rs2506142	2.0×10^{-9}	3.7×10^{-6}
17	rs75213074	7.0×10^{-9}	3.5×10^{-5}
20	rs144017103	1.0×10^{-8}	1.6×10^{-5}

In addition, we have now provided in Supplementary Figure 5 the forest-plots for these 6 loci and as well as for 4 other loci that had been identified in earlier GWAS but failed to reach genome-wide significance in our study. To make this clearer in the main text, we now write on page 9: *"Of the 11 previously reported migraine risk loci that were not GWS in our study, six were GWS in Gormley et al. (2016) and had $P < 3.50 \times 10^{-5}$ in our data, one had $P = 2.37 \times 10^{-3}$, three had $P > 0.14$ and one was not available in our data (Supplementary Fig. 5).".*

References:
- Chasman et al. (2011) Genome-wide association study reveals three susceptibility loci for common migraine in the general population. *Nature Genetics, 43*, 695.
- Freilinger et al. (2012) Genome-wide association analysis identifies susceptibility loci for migraine without aura. *Nature Genetics, 44*(7), 777-782.
- Anttila et al. (2013) Genome-wide meta-analysis identifies new susceptibility loci for migraine. *Nature Genetics, 45*(8), 912-917.
- Pickrell et al. (2016) Detection and interpretation of shared genetic influences on 42 human traits. *Nature Genetics, 48*, 709.

4. Previously, Gormley et al. (2016) conducted subtype-specific GWAS reported that 7 loci were GWS in MO but none was GWS in MA. In current GWAS, three SNPs were MA-specific, two SNPs were MO-specific and nine SNPs were shared. How about the previously reported 7 loci MO-specific in current study?

Response: We thank the Reviewer for this important remark. Gormley et al. (2016) indeed reported 7 loci that had P-value < 5×10^{-8} in MO but did not have a P-value < 5×10^{-8} in MA. Importantly, this observation alone is not evidence that these loci are MO-specific, because they may very well also have non-zero effect in MA even if the effect did not quite reach a P-value < 5×10^{-8} in the data set of Gormley et al. Indeed, in our current study, 2 out of the 7 loci now have a P-value < 5×10^{-8} also in MA.

To properly distinguish subtype-specific variants from those that show effect in both subtypes, we have directly compared the effect size estimates of the subtypes and asked whether they are both non-zero (model BOTH) or whether only one of them is non-zero while the other is zero (models MO and MA). Of the 7 loci that were genome-wide significant in MO but not in MA in 2016 study, 4 had a high probability (> 0.98) to have effects on both subtypes and were among the 9 SNPs that we report being shared by both subtypes. None of the 7 loci had a particularly high probability for being MO-specific (all MO probabilities < 0.65). Thus, the two MO-specific variants we report here, were not among the 7 variants reported by Gormley et al. (2016) as being GWS only in MO but not in MA.

Probabilities for all lead variants across all models are reported in Supplementary Table 12a.

In the manuscript, we write about the 7 loci on page 15: *"We find no evidence that any of the seven loci that were earlier reported GWS in MO, but not in MA by Gormley et al. (2016), would be specific for MO, while four of them (LRP1, FHL5, near FGF6 and near TRPM8) are among the nine loci shared by both subtypes with a probability over 95%.".*

Additionally, we have added to the Methods section (page 28) a statement about the difference between genome-wide significance status and the proper model comparison approach that reads: *"We note that the amount of information in the data ("statistical power") is taken automatically into account in this model comparison, which we consider an advantage compared to a comparison of the raw P-values between the subtype analyses that does not automatically account for statistical power. In particular,*

observing a GWS P-value ($P < 5 \times 10^{-8}$) in one subtype but not in the other subtype is not yet evidence for a subtype specific locus, because the effect could still be non-zero also for the other subtype but simply lack power to reach the stringent GWS threshold."

5. The authors stated that the new risk loci include genes encoding recent migraine-specific drug targets, namely calcitonin gene-related peptide (CALCA/CALCB) and serotonin 1F receptor (HTR1F). Since the drug targets were reported, the contribution of the identification of CALCA/CALCB and HTR1F was limited.
Response: The Reviewer is correct that the two genes are already well-established drug targets. We only highlight these two novel risk loci because they work as a proof-of-principle showing that risk loci discovered from GWAS data like ours indeed surface genes that are targeted by effective migraine drugs. We feel that such discoveries give a well-founded motivation for the reader and the scientific community to study in detail also other loci highlighted by the data in search for candidates for new therapies against migraine.

6. With no doubt, the group indeed identified many novel loci for migraine from the previous meta-analysis of Gormley et al. (2016) to current GWAS. One day, some of these loci may explain the mechanism of the development of migraine, however, till now, the mechanism was elusive based on so many identification. Using GWAS was unlikely to explore the mechanism of migraine. More work should be done.
Response: We can only agree with the Reviewer that more work is needed before the GWAS risk loci have been firmly connected to the mechanisms involved in migraine pathophysiology. Here, we have collected, combined and reported to the scientific community the largest GWAS data set on migraine to date, so it can form a solid basis for much needed future work on migraine (functional) genetics.

Decision Letter, first revision:

Our ref: NG-A56688R

30th July 2021

Dear Matti,

Your revised manuscript "Genome-wide analysis of 102,084 migraine cases identifies 123 risk loci and subtype-specific risk alleles" (NG-A56688R) has been seen by the original referees. As you will see from their comments below, they find that the paper has improved in revision, and therefore we will be happy in principle to publish it in Nature Genetics as an Article pending final revisions to comply with our editorial and formatting guidelines.

We are now performing detailed checks on your paper and will send you a checklist detailing our

editorial and formatting requirements in about a week. Please do not upload the final materials and make any revisions until you receive this additional information from us.

Thank you again for your interest in Nature Genetics Please do not hesitate to contact me if you have any questions.

Sincerely,
Kyle

Kyle Vogan, PhD
Senior Editor
Nature Genetics
https://orcid.org/0000-0001-9565-9665

Reviewer #1 (Remarks to the Author):

The authors have greatly improved methodology, clarity and answered all relevant questions. Congratulations!

Reviewer #2 (Remarks to the Author):

The manuscript is improved from the prior version, providing more precise in analysis and result presentation. As we know, GWASs of migraine have found a large number of novel loci and suggested potential mechanisms. However the potential mechanisms lack evidence to be verified. The authors comment that the study can form a solid basis for much needed future work on migraine (functional) genetics. I think some future work on gene function should be added in this study now.

Author Rebuttal, first revision:

Reviewer #1:

Remarks to the Author:

The authors have greatly improved methodology, clarity and answered all relevant questions. Congratulations!

Response:

We thank the Reviewer for all the valuable and carefully written reviews that improved our manuscript.

Reviewer #2:

Remarks to the Author:

The manuscript is improved from the prior version, providing more precise in analysis and result presentation. As we know, GWASs of migraine have found a large number of novel loci and suggested potential mechanisms. However the potential mechanisms lack evidence to be verified. The authors comment that the study can form a solid basis for much needed

future work on migraine (functional) genetics. I think some future work on gene function should be added in this study now.
Response:
We thank the Reviewer for all the important and valuable comments that improved our manuscript. We agree that functional work is an important next step, but we think it is out of the scope of our current work.

Final Decision Letter:

In reply please quote: NG-A56688R1 Pirinen

22nd November 2021

Dear Matti,

I am delighted to say that your manuscript "Genome-wide analysis of 102,084 migraine cases identifies 123 risk loci and subtype-specific risk alleles" has been accepted for publication in an upcoming issue of Nature Genetics.

Over the next few weeks, your paper will be copyedited to ensure that it conforms to Nature Genetics style. Once your paper is typeset, you will receive an email with a link to choose the appropriate publishing options for your paper and our Author Services team will be in touch regarding any additional information that may be required.

After the grant of rights is completed, you will receive a link to your electronic proof via email with a request to make any corrections within 48 hours. If, when you receive your proof, you cannot meet this deadline, please inform us at rjsproduction@springernature.com immediately.

You will not receive your proofs until the publishing agreement has been received through our system.

Due to the importance of these deadlines, we ask you please us know now whether you will be difficult to contact over the next month. If this is the case, we ask you provide us with the contact information (email, phone and fax) of someone who will be able to check the proofs on your behalf, and who will be available to address any last-minute problems.

Your paper will be published online after we receive your corrections and will appear in print in the next available issue. You can find out your date of online publication by contacting the Nature Press Office (press@nature.com) after sending your e-proof corrections. Now is the time to inform your Public Relations or Press Office about your paper, as they might be interested in promoting its publication. This will allow them time to prepare an accurate and satisfactory press release. Include your manuscript tracking number (NG-A56688R1) and the name of the journal, which they will need when they contact our Press Office.

Before your paper is published online, we will be distributing a press release to news organizations worldwide, which may very well include details of your work. We are happy for your institution or funding agency to prepare its own press release, but it must mention the embargo date and Nature

Genetics. Our Press Office may contact you closer to the time of publication, but if you or your Press Office have any enquiries in the meantime, please contact press@nature.com.

Acceptance is conditional on the data in the manuscript not being published elsewhere, or announced in the print or electronic media, until the embargo/publication date. These restrictions are not intended to deter you from presenting your data at academic meetings and conferences, but any enquiries from the media about papers not yet scheduled for publication should be referred to us.

Please note that Nature Genetics is a Transformative Journal (TJ). Authors may publish their research with us through the traditional subscription access route or make their paper immediately open access through payment of an article-processing charge (APC). Authors will not be required to make a final decision about access to their article until it has been accepted. Find out more about Transformative Journals

<B>Authors may need to take specific actions to achieve compliance with funder and institutional open access mandates. For submissions from January 2021, if your research is supported by a funder that requires immediate open access (e.g. according to Plan S principles), then you should select the gold OA route, and we will direct you to the compliant route where possible. For authors selecting the subscription publication route, our standard licensing terms will need to be accepted, including our self-archiving policies. Those standard licensing terms will supersede any other terms that the author or any third party may assert apply to any version of the manuscript.

Please note that Nature Research offers an immediate open access option only for papers that were first submitted after 1 January 2021.

If you have any questions about our publishing options, costs, Open Access requirements, or our legal forms, please contact ASJournals@springernature.com

If you have posted a preprint on any preprint server, please ensure that the preprint details are updated with a publication reference, including the DOI and a URL to the published version of the article on the journal website.

To assist our authors in disseminating their research to the broader community, our SharedIt initiative provides you with a unique shareable link that will allow anyone (with or without a subscription) to read the published article. Recipients of the link with a subscription will also be able to download and print the PDF.

As soon as your article is published, you will receive an automated email with your shareable link.

You can now use a single sign-on for all your accounts, view the status of all your manuscript submissions and reviews, access usage statistics for your published articles and download a record of your refereeing activity for the Nature journals.

An online order form for reprints of your paper is available at https://www.nature.com/reprints/author-reprints.html. Please let your coauthors and your institutions' public affairs office know that they are also welcome to order reprints by this

natureresearch

method.

If you have not already done so, we invite you to upload the step-by-step protocols used in this manuscript to the Protocols Exchange, part of our on-line web resource, natureprotocols.com. If you complete the upload by the time you receive your manuscript proofs, we can insert links in your article that lead directly to the protocol details. Your protocol will be made freely available upon publication of your paper. By participating in natureprotocols.com, you are enabling researchers to more readily reproduce or adapt the methodology you use. Natureprotocols.com is fully searchable, providing your protocols and paper with increased utility and visibility. Please submit your protocol to https://protocolexchange.researchsquare.com/. After entering your nature.com username and password you will need to enter your manuscript number (NG-A56688R1). Further information can be found at https://www.nature.com/nprot/.

Sincerely,
Kyle

Kyle Vogan, PhD
Senior Editor
Nature Genetics
https://orcid.org/0000-0001-9565-9665
